# Discovery of *tert*-Butyl Ester Based 6-Diazo-5-oxo-l-norleucine Prodrugs for Enhanced Metabolic Stability and Tumor Delivery

**DOI:** 10.1021/acs.jmedchem.3c01681

**Published:** 2023-11-10

**Authors:** Kateřina Novotná, Lukáš Tenora, Eva Prchalová, James Paule, Jesse Alt, Vijay Veeravalli, Jenny Lam, Ying Wu, Ivan Šnajdr, Sadakatali Gori, Vijaya Saradhi Mettu, Takashi Tsukamoto, Pavel Majer, Barbara S. Slusher, Rana Rais

**Affiliations:** ^†^Johns Hopkins Drug Discovery, ^‡^Departments of Neurology, ^§^Psychiatry and Behavioral Sciences, ^∥^Pharmacology and Molecular Sciences, ^⊥^Neuroscience, ^#^Medicine, and ^∇^Oncology, Johns Hopkins School of Medicine, Baltimore, Maryland 21205, United States; ¶ Institute of Organic Chemistry and Biochemistry v.v.i., Academy of Sciences of the Czech Republic, Prague 160 00, Czech Republic; ◆ Department of Organic Chemistry, Faculty of Science, Charles University, Prague 128 00, Czech Republic

## Abstract

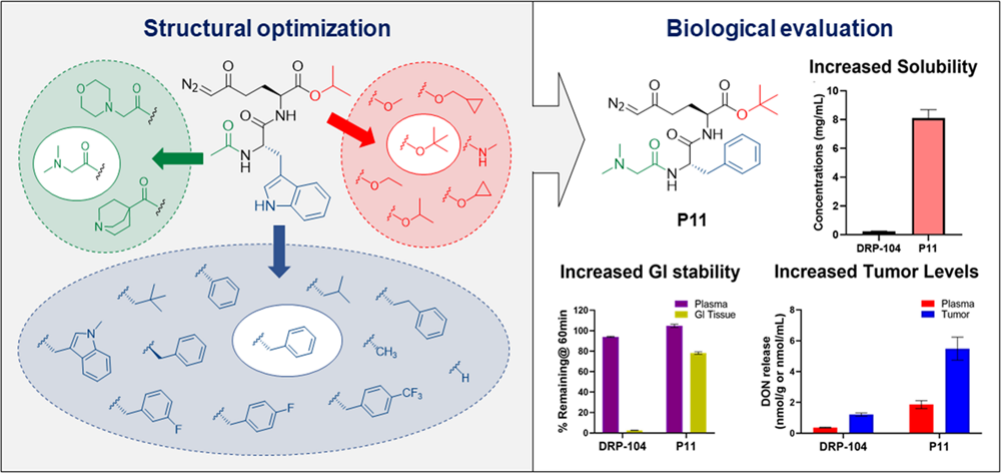

The glutamine antagonist 6-diazo-5-oxo-l-norleucine (DON) exhibits remarkable anticancer efficacy; however, its therapeutic potential is hindered by its toxicity to gastrointestinal (GI) tissues. We recently reported the discovery of DRP-104, a tumor-targeted DON prodrug with excellent efficacy and tolerability, which is currently in clinical trials. However, DRP-104 exhibits limited aqueous solubility, and the instability of its isopropyl ester promoiety leads to the formation of an inactive M1-metabolite, reducing overall systemic prodrug exposure. Herein, we aimed to synthesize DON prodrugs with various ester and amide promoieties with improved solubility, GI stability, and DON tumor delivery. Twenty-one prodrugs were synthesized and characterized in stability and pharmacokinetics studies. Of these, **P11**, *tert*-butyl-(*S*)-6-diazo-2-((*S*)-2-(2-(dimethylamino)acetamido)-3-phenylpropanamido)-5-oxo-hexanoate, showed excellent metabolic stability in plasma and intestinal homogenate, high aqueous solubility, and high tumor DON exposures and preserved the ideal tumor-targeting profile of DRP-104. In conclusion, we report a new generation of glutamine antagonist prodrugs with improved physicochemical and pharmacokinetic attributes.

## Introduction

Glutamine is the most abundant amino acid in the mammalian body. Its metabolism serves as a fundamental source of nitrogen and carbon, providing the essential building blocks for the biosynthesis of amino acids, nucleotides, fatty acids, and coenzymes.^1^ Glutamine uptake and utilization are greatly increased in cancer cells due to the increased energy demand required for rapid proliferation^2^ and can lead to an oncogene-dependent addiction to glutamine.^3^ Thus, blocking glutamine metabolism, particularly in cancer cells, serves as a rational therapeutic approach for cancer.

**Figure 1:**
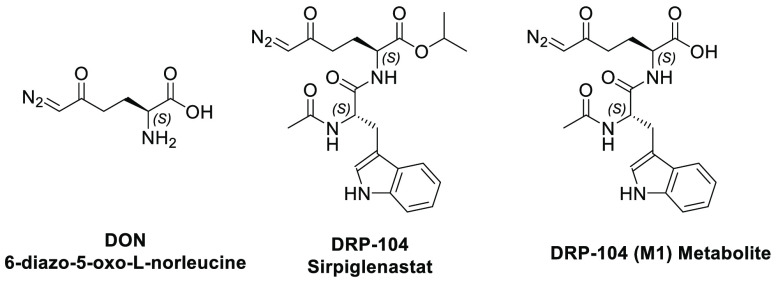
Chemical structures of DON (6-diazo-5-oxo-l-norleucine), DRP-104 (Sirpiglenastat), and DRP-104 (M1) metabolite.

6-Diazo-5-oxo-l-norleucine (DON; Figure 1) is a glutamine antagonist with antitumor efficacy demonstrated in multiple preclinical studies^4−7^ as well as in several clinical trials.^8−15^ In one of the earliest clinical studies, 66% of patients demonstrated disease stability or regression following 2 weeks or more of DON therapy.^16^ Further, in children with hematologic malignancies on standard 6-mercaptopurine (6-MP) therapy, DON combination led to complete bone marrow remissions in 42% of patients, showing remarkable superiority to 6-MP monotherapy.^17^ However, its further clinical evaluation was aborted due to dose-limiting gastrointestinal (GI) toxicity, as GI cells are highly glutamine-utilizing. To revamp DON’s clinical translation, prodrug strategies have been employed to develop GI-stable analogues that remain intact and inactive in the gut while preferentially bioactivating to DON within the cancer cells.^18,19^

For example, the previously reported DON prodrug termed JHU-083 was shown to cause significant tumor regression in several mouse models at doses that were well-tolerated and lacked GI toxicities.^20−22^ In addition, JHU-083 was shown to markedly increase endogenous antitumor immunity and provide robust and durable antitumor effects when combined with anti-PD-1 therapy.^21,23,24^ Recently, we reported the discovery of DRP-104 (Figure 1), a dipeptide prodrug consisting of an *N*-acetyl tryptophan moiety on the amino group of DON isopropyl ester.^25^ DRP-104 was shown to be preferentially transformed to DON in tumor cells resulting in an 11-fold greater delivery of DON to tumor versus GI tissues. DRP-104 caused robust inhibition of tumor growth in mice, similar to equimolar DON, but with markedly reduced GI side effects. Additionally, DRP-104 showed added benefits when combined with PD-1 therapy.^25^ Given this promising profile, DRP-104 was selected for clinical development as a single agent, as well as in combination with immunotherapy (identifier NCT04471415). While DRP-104 showed promising pharmacokinetics and robust efficacy in preclinical studies, it was metabolized to a charged, inactive metabolite, M1: (*S*)-2-((*S*)-2-acetamido-3-(1*H*-indol-3-yl)propanamido)-6-diazo-5-oxohexanoic acid.^25^ In addition, DRP-104 showed poor aqueous solubility (<1 mg/mL), necessitating formulation approaches for systemic administration.

In an attempt to discover prodrugs with improved stability, solubility, and DON tumor delivery, we designed and evaluated a series of tripeptide-based prodrugs of DON. We initially optimized moieties on DON’s carboxylate employing simple alkyl esters, cyclic esters, and amides. Next, using the GI-stable *t*ert-butyl ester, we explored various acyl moieties at the amino group of the tryptophan residue on DRP-104. Lastly, we replaced the tryptophan on DRP-104 with smaller aromatic and aliphatic amino acids. These systematic structural changes improved the prodrugs’ physicochemical and pharmacokinetic properties.

## Chemistry

**Scheme 1:**
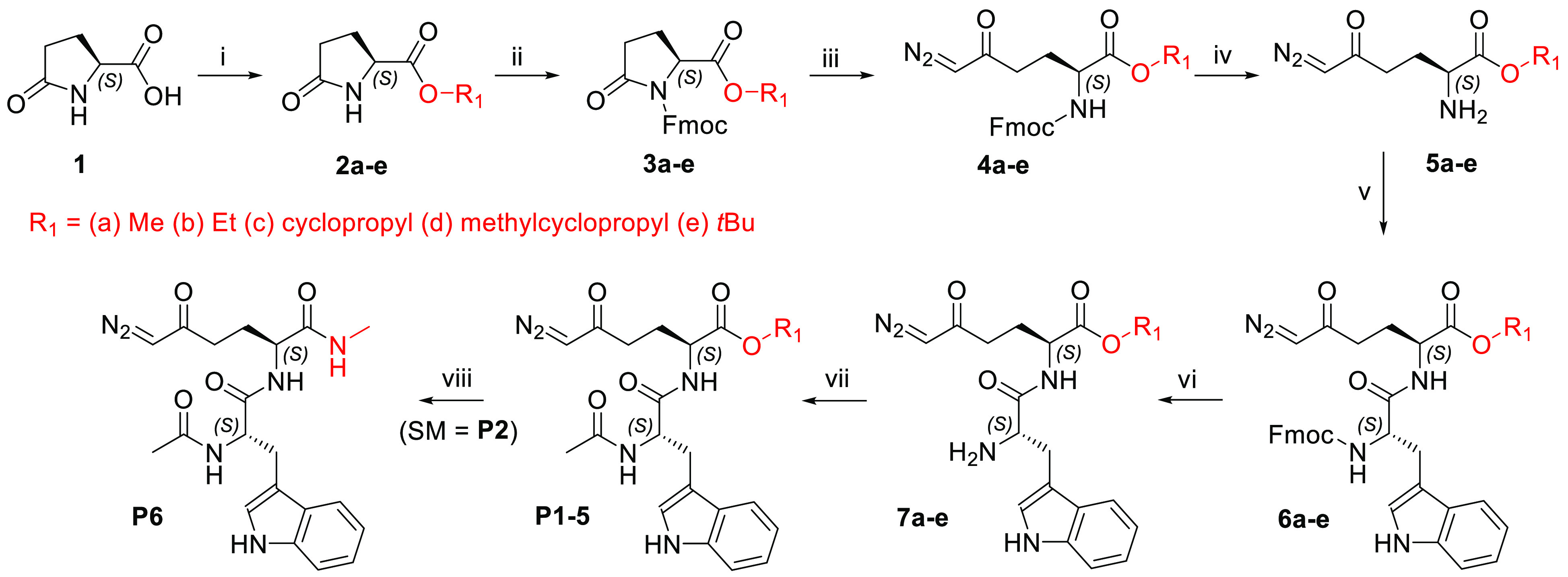
Synthesis of Prodrugs **P1**–**P6** Reagents and conditions: (i) for **2a** and **2b**, R_1_–OH (MeOH or EtOH), SOCl_2_, 0 °C to rt, 16 h, 93–98%; for **2c** and **2d**, R_1_–OH (cyclopropanol or cyclopropylmethanol), DCC, DMAP, DCM, rt, 16 h, 96–97%; for **2e**, *tert*-butyl acetate, perchloric acid, rt, 48 h, 90%; (ii) Fmoc-Cl, LiHMDS, THF, −78 °C to rt, 16 h, 63–95%; (iii) TMSCHN_2_, *n*-BuLi, THF, −78 °C, 3 h, 47–67%; (iv) piperidine, DCM, rt, 3 h, 49–67%; (v) Fmoc-l-Trp-OH, HATU, DIPEA, DCM, or DCM/DMF 4:1, 0 °C to rt, 1.5 h, 73–95%; (vi) diethylamine, DCM, rt, 3–6 h, 90–95%; (vii) Ac_2_O, py, DMF, rt, 3–15 h, 66–92%; (viii) (SM = **P2**), 2 M methylamine in MeOH, 60 °C, 20 h, 65%.

DRP-104 (isopropyl (*S*)-2-((*S*)-2-acetamido-3-(1*H*-indol-3-yl)-propanamido)-6-diazo-5-oxo-hexanoate; also known as Sirpiglenastat) was identified as a lead glutamine antagonist with efficacy in multiple murine cancer models, including enhancement of immunotherapy.^25−28^ Metabolite identification (MET ID) studies revealed formation of the charged M1 metabolite via metabolism of the isopropyl ester.^25^ This metabolite was shown to be inert and inactive presumably leading to reduced systemic intact prodrug exposure.^25^ In an attempt to increase the stability of the ester moiety, we systematically replaced the isopropyl ester with several simple alkyl esters, cyclic esters, and amide. We introduced methyl (**P1**) and ethyl (**P2**) esters, which are commonly found in FDA-approved prodrugs.^29−31^ To enhance the metabolic stability of the ester promoiety, we synthesized sterically hindered cyclic ester based prodrugs, including cyclopropyl (**P3**) and methyl cyclopropyl (**P4**), and branched *tert*-butyl ester (**P5**).^32,33^ Prodrugs **P1**–**P5** were synthesized by a seven-step procedure similar to our previously reported method (Scheme 1).^18^ Briefly, l-pyroglutamic acid **1** was converted to the respective pyroglutamate esters **2a**–**2e** by reaction with thionyl chloride in methanol (**2a**) or ethanol (**2b**), Steglich esterification (**2c** and **2d**), or acid catalyzed transesterification (**2e**). Ester intermediates **2a**–**2e** were protected as Fmoc carbamates (**3a**–**3e**) using Fmoc-Cl and LiHMDS. The reaction of diazo(trimethylsilyl)methyllithium salt with protected pyroglutamate esters **3a**–3**e** afforded the corresponding diazo ketones **4a**–**4e**. Piperidine-mediated deprotection of the Fmoc group in **4a**–**4e** gave free amines **5a**–**5e**, which were coupled to Fmoc-l-Trp-OH activated with HATU in the presence of DIPEA to yield the corresponding dipeptides **6a**–**6e**. The Fmoc protecting group was then removed by piperidine, resulting in amines **7a**–**7e**, which were subsequently acetylated with acetic anhydride to afford the final prodrugs **P1**–**P5**. Notably, as illustrated in Table 1, there was a consistent increase in lipophilicity, as measured by cLogP (calculated using ChemDraw Professional 16.0), with the extension of ester chain length. This increase is also supported by cLogD_7.4_ (Table S1). For the sake of simplicity, we will primarily discuss cLogP in this context. The lipophilicity values for **P1**–**P5** ranged from −0.05 to 1.19, with the *tert*-butyl variant exhibiting the highest cLogP of approximately 1.2. Notably, this value exceeded that of DRP-104, which measured 0.79. Next, the simple aliphatic methylamide prodrug **P6** was synthesized by modifying DON’s carboxylic acid portion to an amide, as amides are known to be typically more resistant to cleavage compared to esters.^34,35^ However, this modification reduced the cLogP to a negative value of −0.91, suggesting high polarity and poor penetration to cellular membranes including tumor cells. As outlined in Scheme 1, amide analogue **P6** was prepared from prodrug **P2** in a one-step procedure using a methanolic solution of methylamine. The aim was to maximize DON delivery to the tumor while maintaining stability at off-target sites.

**Table 1:**
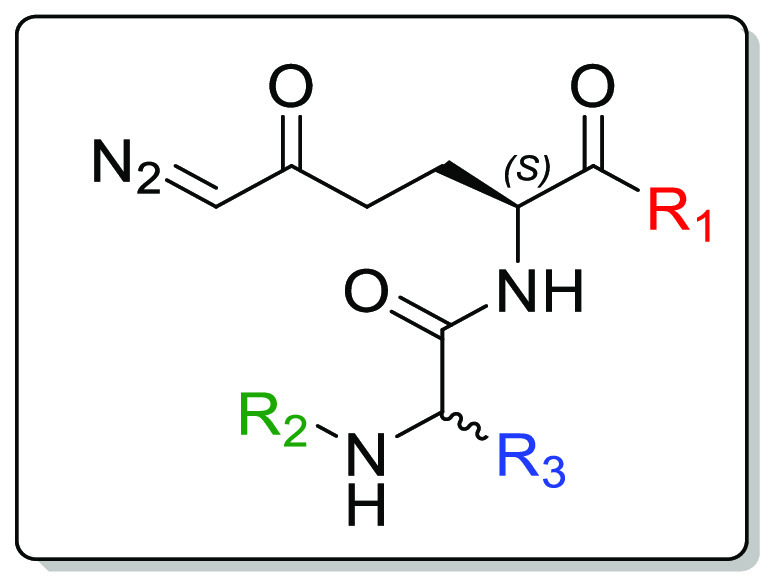

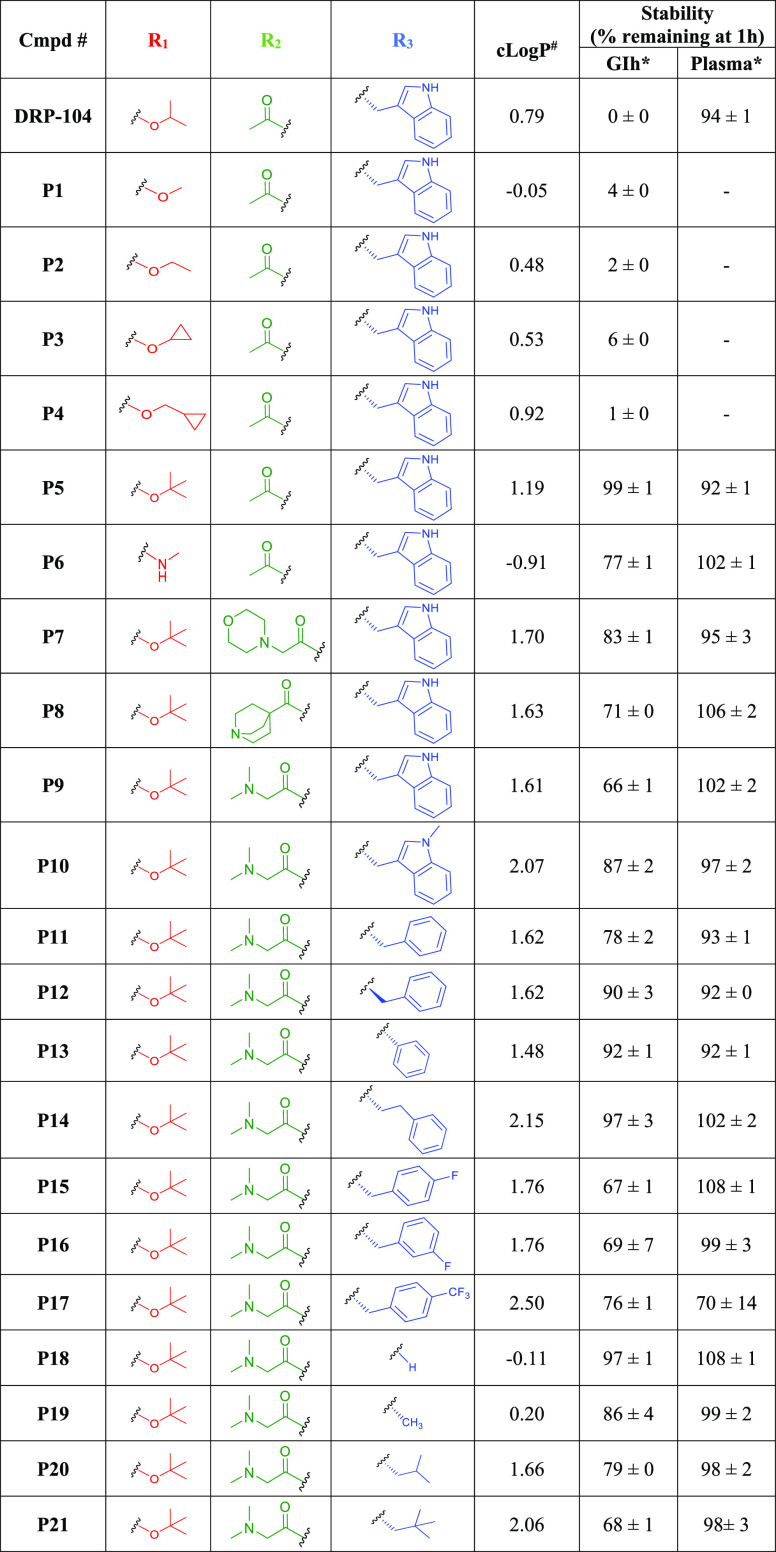
cLogP and Stability of Prodrugs **P1**–**P21** in Mouse Plasma and Intestinal Homogenate (GIh)^a^

a #, calculated using ChemDraw professional 16.0. ∗, CES1^–\–^ mice intestinal homogenate (GIh) and plasma were used for stability assay.

**Scheme 2:**
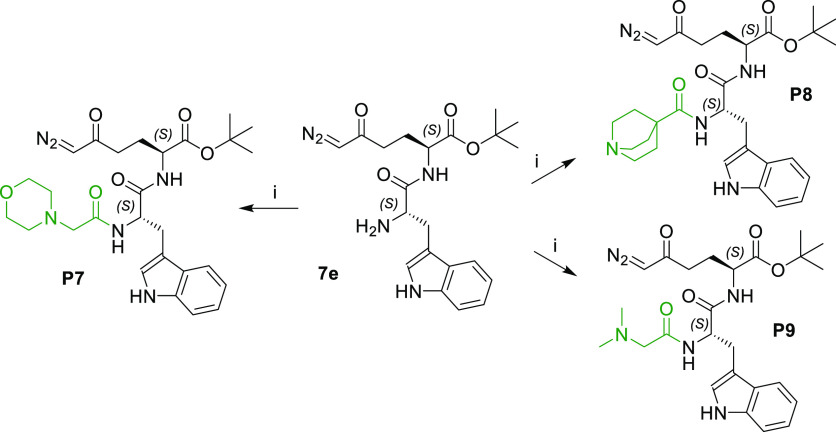
Synthesis of Prodrugs **P7**–**P9** Reagents and conditions: (i) R–COOH (for **P7**, morpholinoacetic acid hydrochloride; for **P8**, quinuclidine-4-carboxylic acid hydrochloride; for **P9**, dimethylglycine), HATU, DIPEA, DMF, 0 °C to rt, 2.5 h, 82–89%.

Following optimization of the carboxylate moiety for stability, modifications were made to the acyl moiety of the tryptophan residue to enhance tumor delivery and prodrug solubility. To achieve this, the acetyl group of DRP-104 was replaced with morpholinomethyl (**P7**), quinuclidinyl (**P8**), and dimethylglycinyl (**P9**) (Scheme 2) to enhance cLogP to 1.61–1.70. These prodrugs were synthesized in one step from intermediate **7e** using conditions for amide coupling in the presence of the appropriate carboxylic acid, i.e., morpholinoacetic acid, quinuclidine-4-carboxylic acid, or dimethylglycine, for **P7**, **P8**, and **P9**, respectively, in the presence of HATU and DIPEA.

**Scheme 3:**
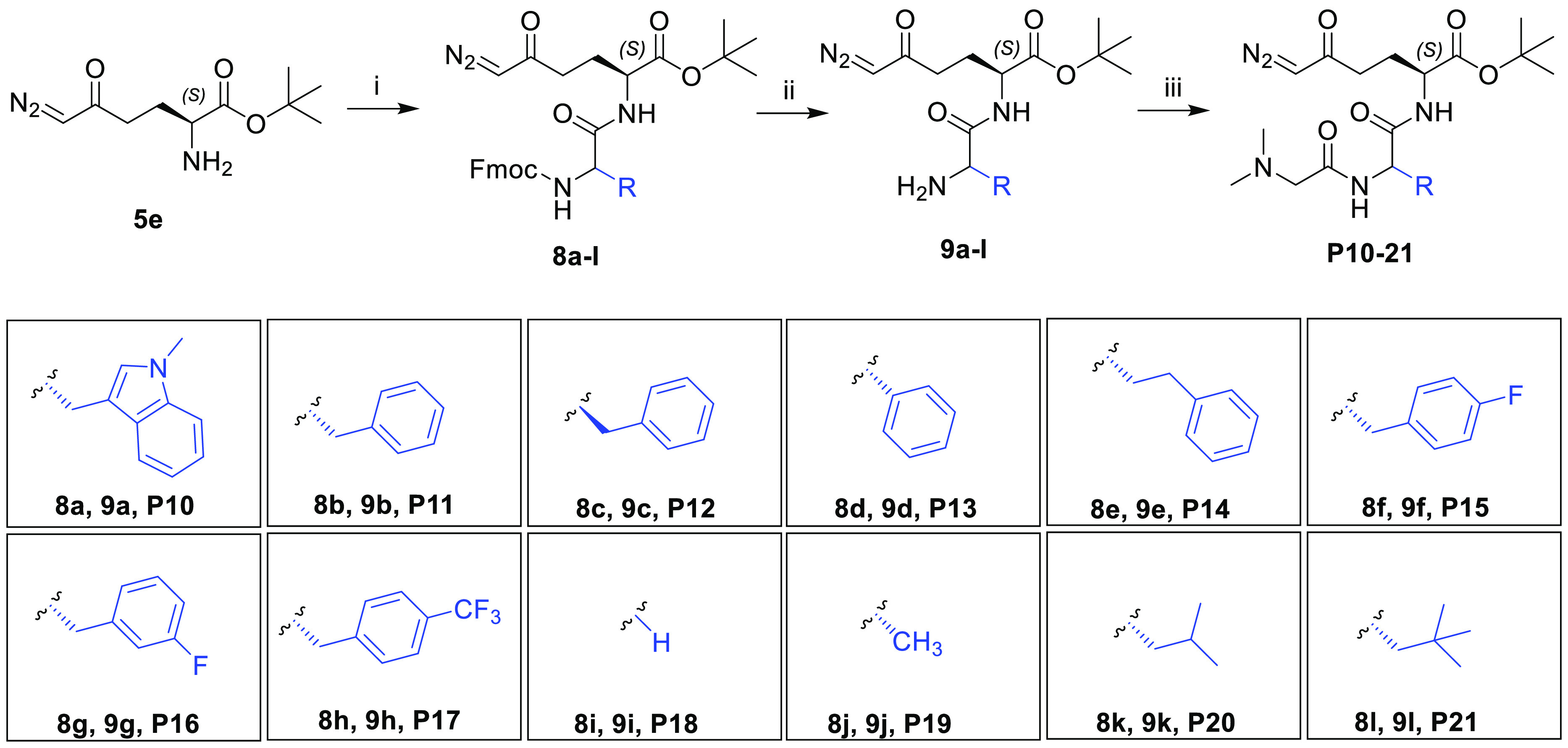
Synthesis of Prodrugs **P10**–**P21** Reagents and conditions: (i) Fmoc-AA-OH (**8a**, Fmoc-l-Trp(*N*-Me)-OH; **8b**, Fmoc-l-Phe-OH; **8c**, Fmoc-d-Phe-OH; **8d**, Fmoc-l-Phg-OH; **8e**, Fmoc-l-HomoPhe-OH; **8f**, Fmoc-l-Phe(4-F)-OH; **8g**, Fmoc-l-Phe(3-F)-OH; **8h**, Fmoc-l-Phe(4-CF_3_)-OH; **8i**, Fmoc-Gly-OH; **8j**, Fmoc-l-Ala-OH·H_2_O; **8k**, Fmoc-l-Leu-OH; **8l**, Fmoc-l-Ala(β-*t*Bu)-OH), HATU, DIPEA, DCM, 0 °C to rt, 1.5–16 h, 68–98%; (ii) diethylamine, DCM, rt, 1.5–7 h, 76–96%; (iii) for **P10**, **P11**, **P18**, and **P19**, dimethylglycine, HATU, DIPEA, DCM, or DMF, 0 °C to rt, 1.5–2.5 h, 64–73%; for **P12**–**P17**, **P20**, and **P21**, 2,5-dioxopyrrolidin-1-yl dimethylglycinate, DCM, rt, 2–20 h, 51–92%.

In the last part of our structure–property optimization study, we changed the structure by focusing on the amino acid of our tripeptide prodrugs. DRP-104 has low intrinsic aqueous solubility (<1 mg/mL). Thus, we aimed to identify the minimum structural requirements for tumor-targeted delivery with enhanced solubility, stability, and pharmacokinetic properties. As outlined in Scheme 3, we synthesized the prodrugs by replacing the tryptophan on DRP-104 with aromatic (**P10**–**P17**) and aliphatic amino acids (**P18**–**P21**), including standard (**P11**, **P18**–**P20**) and nonstandard amino acids (**P10**, **P13**–**P17**, and **P21**), fluorinated amino acids (**P15**–**P17**), and d-amino acid (**P12**). Prodrugs **P10**–**P21** were prepared in a three-step synthetic procedure starting with intermediate **5e**. Dipeptides **8a**–**8l** were synthesized by a standard HATU coupling reaction between the appropriate Fmoc-protected amino acids and compound **5e**. The Fmoc group was removed by diethylamine to afford intermediates **9a**–**9l** in good to excellent yields. Final prodrugs **P10**–**P21** were prepared by two different coupling conditions—with dimethylglycine activated with HATU in the presence of DIPEA (**P10**, **P11**, **P18**, **P19**) or with 2,5-dioxopyrrolidin-1-yl dimethylglycinate^36^ (**P12**–**P17**, **P20**, **P21**). Most of the synthesized prodrugs (**P10**–**P17**, **P20**, **P21**) retained a degree of lipophilicity (cLogP from 1.48 to 2.50) similar to that of DRP-104, except for prodrugs **P18** (0.20) and **P19** (−0.11) containing a smaller glycine or alanine moiety.

## Results and Discussion

### Screening Strategy

**Figure 2:**
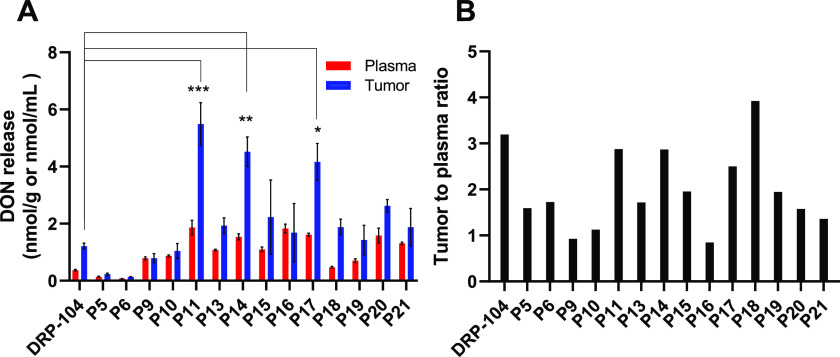
Single time point pharmacokinetic screening of selected prodrugs. Prodrugs (1 mg/kg DON equivalent) were administered subcutaneously (SC) to C57BL/6/CES1^–/–^ mice and (A) DON levels released in plasma (red) and tumor (blue) were measured 30 min post dose and (B) the tumor to plasma ratio of released DON was calculated. Data expressed as mean ± SEM, *n* = 3. ∗, *p* < 0.05; ∗∗, *p* < 0.01; and ∗∗∗, *p* < 0.001, versus DRP-104 tumor levels (one-way ANOVA with Dunnett’s *post hoc* test).

**Figure 3:**
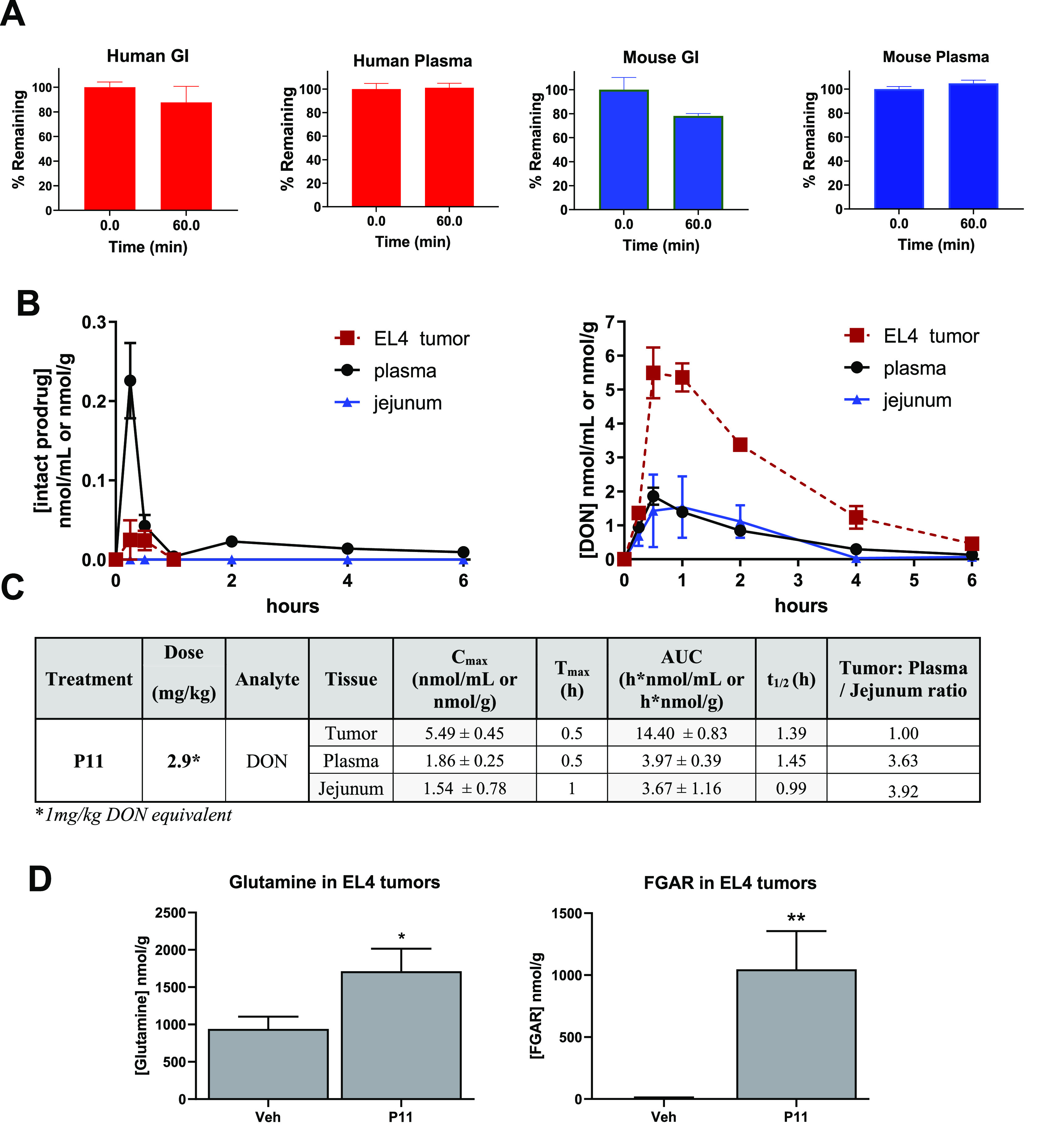
Stability, pharmacokinetic analysis, and tumor target engagement of **P11**. (A) Stability of **P11** in human GI microsomes and plasma and mouse GI homogenate and mouse plasma. (B, C) **P11** (2.9 mg/kg) was administered subcutaneously (SC) to C57BL/6/CES1^–/–^ mice bearing EL4 tumors, and tissues were harvested and analyzed for (B) intact (**P11**) and released DON in tumor, plasma, and jejunum. (C) PK parameters of released DON. Data expressed as mean ± SEM, *n* = 3. EL4 tumors collected from these mice at *T*_max_ were used for quantification of (D) tumor glutamine and FGAR quantification at 30 min post dose for target engagement evaluation (mean ± SD; ∗, *p* > 0.01; ∗∗, *p* > 0.001; unpaired two-tailed *t* test).

**Figure 4:**
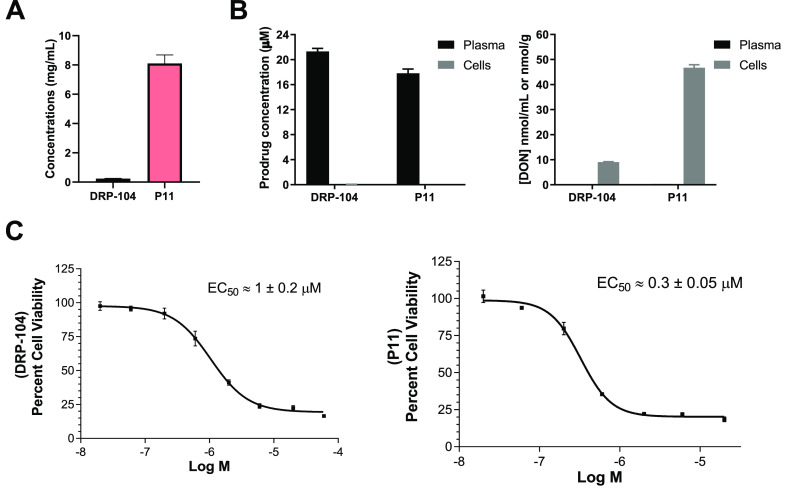
Comparison of solubility, human tumor cell to plasma partitioning of DRP-104 and **P11**, and their antiproliferative effect. (A) Aqueous solubility of DRP-104 and **P11** in buffer at pH 7.4. (B) Human tumor cell to plasma partitioning of DRP-104 and **P11** were conducted by incubating either compound for 1 h in P493B lymphoma cells suspended in human plasma. Both intact DRP-104 and **P11** levels were measured in human plasma and tumor cells, as well as prodrug-derived DON release in human plasma and tumor cells. (C) Cell viability assay was performed using P493B lymphoma cells incubated with DRP-104 and **P11** for 72 h. Nonlinear regression analysis of the log-transformed data gave EC_50_ values.

The goal herein was to obtain prodrugs that could be effectively delivered to tumor cells while retaining stability in both the GI tract and plasma. To accomplish this, all prodrugs were systematically tested using a predefined screening paradigm. Drugs that were found to be stable in mice intestinal homogenate (GIh; >50% remaining at 1 h) were evaluated for stability in mice plasma (Table 1). Prodrugs showing stability in both matrices (>50% remaining at 1 h) were next evaluated in a single time point pharmacokinetic study in mice where plasma and tumor levels of DON were quantified (Figure 2). The prodrug with the best tumor DON levels and tumor/plasma ratio was then characterized in a full pharmacokinetic study in mice with functional tumor target engagement assessment (Figure 3). Selected prodrugs were also assessed for solubility, human tumor cell partitioning in a human plasma/tumor cell suspension assay, and human tumor cell viability assay (Figure 4) as detailed below.

### Characterization of Metabolic Stability and Single Time Point Pharmacokinetics of Prodrugs **P1**–**P21**

Considering that the GI tract was the primary site of DON toxicity in clinical studies,^8,14,16^ minimizing DON release at this site was crucial. Thus, we sought to improve the GI stability of newly designed prodrugs. **P1** with methyl, **P2** with ethyl, **P3** with cyclopropyl, and **P4** with cyclopropylmethyl esters were all found to be unstable (<10% remaining at 1 h) in the GI homogenate as shown in Table 1. In contrast, **P5** (*tert*-butyl ester) and **P6** (methyl amide) were found to be stable (>50% remaining at 1 h). All subsequent prodrugs **P7**–**P21** synthesized with the *tert*-butyl esters at the DON carboxylate, irrespective of the moieties at positions R_2_ and R_3_, were stable in the GI homogenate. For the stability assay, CES^–/–^ mice^37^ were used, as these mice are generated by inactivating the CES1 gene such that there is undetectable CES activity in plasma but normal activity in tissues including the GI tissue. These data indicated that the primary site of metabolism for all prodrugs in the GI tract was the ester hydrolysis that occurred likely by the action of carboxylesterase enzyme CES1, as we have previously demonstrated.^25^ Interestingly, the *tert*-butyl ester was resistant to hydrolysis in GI tissue. Next, all the GI-stable prodrugs were evaluated in mouse plasma. Interestingly, all GI-stable prodrugs also exhibited stability in plasma with >50% remaining after a 1 h incubation. We next evaluated the plasma- and GI-stable prodrugs in a single time point pharmacokinetic (PK) study in CES1^–/–^ mice bearing EL4 tumor. The C57BL/6/CES1^–/–^ mice were generated by inactivating the CES1 gene such that there is undetectable CES activity in plasma but normal activity in tissues.^37^ CES1^–/–^ mice were used as they mimic the distribution of CES1 in humans.^38^ These mice are often used in preclinical prodrug studies, including prior studies with DRP-104.^25^ The prodrugs were dosed subcutaneously (SC) at a dose of 1 mg/kg DON equivalent (*n* = 3 mice/group). After 30 min, the mice were sacrificed, and plasma and tumor samples were collected to measure the levels of released DON. This 30 min time point was selected as it corresponded to the time resulting in the maximal concentration of DON release following DRP-104 administration.^25^ Because maintaining a high tumor-to-plasma ratio was important, we quantified the release of DON in both plasma and tumor. The results from the single time point analysis of the DON release from prodrugs are shown in Figure 2. Of the 15 prodrugs evaluated, administration of **P11** (5.49 ± 1.3 μM; *p* < 0.001), **P14** (4.52 ± 0.90 μM; *p* < 0.01), and **P17** (4.16 ± 1.13 μM; *p* < 0.05) led to significantly higher tumor concentrations of DON compared to administration of equimolar DRP-104 (1.21 ± 0.18 μM). Of these, **P11** showed the highest tumor DON delivery with >4.5 higher DON levels compared to equimolar DRP-104. Most prodrugs, except for **P5**, **P6**, **P18**, and **P19**, also showed higher DON plasma levels compared to DRP-104. Notably, **P11** and **P14** maintained the preferential DON tumor versus plasma delivery as was observed for DRP-104. Given that **P11** exhibited preferential tumor delivery and provided the highest DON tumor levels, it was selected to undergo a full-time-course pharmacokinetic evaluation as well as target engagement in EL4 tumor-bearing mice.

### Stability, Pharmacokinetics, and Tumor Target Engagement of **P11**

The stability of **P11** was confirmed in mouse and human plasma, as well as in GI matrices (Figure 3A). The results indicate similar stability between the two species, validating that the mouse model was suitable for PK studies. PK evaluation of **P11** was performed in CES1^–/–^ mice bearing flank murine EL4 lymphoma tumors. **P11** was dosed via a subcutaneous (SC) route at 2.9 mg/kg (1 mg/kg DON equivalent dose), and plasma, tumor, and GI tissues were collected 0–6 h post dose. Tissues were analyzed for both the intact prodrug and DON release from the prodrug, using liquid chromatography with tandem mass spectrometry as we have previously described, with minor modifications.^19,25^Figure 3B,C illustrate the pharmacokinetic profile of **P11** following subcutaneous dosing. **P11** exhibited excellent pharmacokinetics, delivering DON preferentially to tumor cells with a maximum concentration (*C*_max_) of 5.49 ± 0.75 nmol/g compared to plasma (1.86 ± 0.25 nmol/mL) and intestinal tissue (1.54 ± 0.91 nmol/g), which were approximately 3-fold lower. In terms of overall exposure, **P11** delivered approximately 3.6-fold higher tumor exposure of DON (area under curve, AUC_0–*t*_ = 13.7 ± 0.90 h·nmol/g) versus that of plasma (3.8 ± 0.37 h·nmol/mL) and 4.4-fold higher tumor exposure versus that of jejunum (AUC = 3.13 ± 0.87 h·nmol/g). Intact prodrug **P11** showed low levels in all matrices including plasma (AUC = 0.15 h·nmol/mL) and tumor (0.092 h·nmol/g). All intestinal tissue levels for the intact prodrug were below the limit of quantification (0.01 nmol/mL). These *in vivo* results confirmed preferential tumor distribution and efficient conversion of **P11** to DON. We further confirmed target engagement of **P11** by assessing the levels of glutamine and formylglycinamide ribonucleotide (FGAR) at the *T*_max_ in tumor (30 min) (Figure 3D). These biomarkers were previously demonstrated to be significantly affected by DON treatment serving as efficient target engagement tools.^39,40^ We observed a significant, nearly 2-fold, rise in glutamine (from 941 ± 95 to 1710 ± 173 nmol/g) in tumors treated with **P11** compared with tumor treated with vehicle. Similarly, there was a substantial 150-fold increase in FGAR (from 7.00 ± 3.00 to 1040 ± 179 nmol/g) in tumors treated with **P11**, as we have previously reported with other glutamine antagonist prodrugs.^39,40^ The increase in FGAR is observed due to DON’s inhibition of the enzyme FGAR amidotransferase (FGAR-AT) that catalyzes the ATP-dependent amidation of FGAR to formylglycinamidine ribonucleotide (FGAM) using glutamine as source of the amidic nitrogen.^25^ These data confirmed that **P11** was effective at delivering DON to tumor and inhibiting the relevant mechanistic pathways.

### Solubility, Human Tumor Cell Partitioning, and Antiproliferation Efficacy Assessment of **P11**

Next, **P11** and DRP-104 were evaluated for their solubility, their ability to permeate and be cleaved to DON in human P493B lymphoma cells incubated in human plasma, and their ability to inhibit proliferation of human P493B lymphoma cells. Figure 4A illustrates the aqueous solubility of **P11** (8.1 ± 1.1 mg/mL), which was 33 times greater than that of DRP-104 (0.24 ± 0.03 mg/mL). The chemical stability of prodrugs P**11** and DRP-104 was evaluated in tandem using high resolution mass spectrometry (HRMS), confirming both prodrugs remained intact without any degradation during the solubility assay (Figure S1A–D). Figure 4B shows the tumor cell partitioning results where both DRP-104 and **P11** were stable in human plasma with no DON release. In contrast, in the tumor cells, both DRP-104 and **P11** showed both partitioning into and biotransformation to DON with tumor cell to plasma partitioning ratios of 180 and 140, respectively. Similar to the *in vivo* mouse studies, **P11** provided a 5-fold increase in DON tumor cell levels when compared to DRP-104 (46.7 ± 1.2 μM versus 9.1 ± 0.15 μM). Moreover, consistent with their high cell partitioning, DRP-104 and **P11** both exhibited excellent antiproliferative activity in a P493B lymphoma cell viability assay. A dose-dependent decrease in cell proliferation was observed following 72 h of incubation (Figure 4C). **P11** caused a leftward shift in the viability curve where nonlinear regression analysis of the log-transformed data gave EC_50_ values for DRP-104 and **P11** at 1 ± 0.2 and 0.30 ± 0.05 μM, respectively.

## Conclusions

Over 20 prodrugs were systematically synthesized and characterized; among these, prodrug **P11** emerged as the most promising. **P11** showed metabolic stability in the GI tract and plasma and exhibited a >30-fold solubility improvement when compared to DRP-104. Additionally, in mice bearing flank EL4 lymphoma tumors, administration of **P11** led to enhanced tumor DON exposure as well as significant increases in glutamine and FGAR levels, confirming target engagement. Furthermore, we evaluated the prodrug **P11** in a human P493 lymphoma cell partitioning assay, where we confirmed the preferential tumor distribution and bioactivation of **P11** to DON, with minimal DON release in plasma. Lastly, **P11** exhibited excellent potency in a human tumor cell viability assay. In sum, we present the discovery of a new generation of DON prodrugs with improved biopharmaceutic and pharmacokinetic properties.

Importantly, it is crucial to highlight that even though we achieved significant progress in enhancing gastrointestinal (GI) stability, increasing overall DON tumor exposure, and successfully attaining a preferential tumor-targeting effect with **P11**, our study did not encompass *in vivo* assessments for dose-dependent toxicity or efficacy testing in tumor models. These investigations, which would substantiate that our findings translate into an enhanced therapeutic window, will be explored in our future research. In line with this, it should be noted that the DON AUC_tumor:plasma_ ratio with **P11** was similar to that with DRP-104. Furthermore, the AUC_tumor:GI tissue_ ratio of ∼4 achieved by **P11** is about 2–3-fold lower than those observed for other prodrugs, including DRP-104.^25,41^ Nonetheless, the systematic prodrug design strategies employed to identify **P11** in this study can serve as a valuable blueprint for enhancing the pharmacokinetic profile and stability of other prodrugs with suboptimal properties and enable their clinical development.

## Experimental Section

Commercially available reagents or HPLC grade solvents and materials were used for the synthesis of compounds described. All chemicals were reagent grade, purchased from Sigma-Aldrich, TCI, Combi-Blocks, AK Scientific, AstaTech. or Iris Biotech GmbH, and were used without further purification. TLC was performed on silica gel 60 F254 coated aluminum sheets (Merck), and spots were visualized with UV light and by the solution of Ce(SO_4_)_2_ × 4H_2_O (1%) and H_3_P(Mo_3_O_10_)_4_ (2%) in sulfuric acid (10%). Column chromatography was performed on silica gel 60 (0.040–0.063 mm, Fluka) or on a Biotage Isolera One Flash Chromatography System using SiliCycle SiliaSep cartridges with silica gel grade 40–63 μm. NMR spectra were measured on Bruker AVANCE 400 or Varian Oxford 500 instruments. ^1^H NMR were recorded at 401 or 500 MHz, and signals of TMS (δ 0.0, CDCl_3_), CDCl_3_ (δ 7.26), and *d*_6_-DMSO (δ 2.50, 3.33) were used for standardization. ^13^C NMR spectra were recorded at 101 or 125 MHz, and the signal of CDCl_3_ (δ 77.16) or *d*_6_-DMSO (δ 39.52) was used for standardization. The chemical shifts are given in δ scale; the coupling constants *J* are given in hertz. The low resolution ESI mass spectra were recorded using a ZQ micromass mass spectrometer (Waters) or an Agilent 1200 series HPLC system. High resolution ESI mass spectra were recorded using an LTQ Orbitrap XL spectrometer (Thermo Fisher Scientific). Preparative HPLC purification was performed on an Agilent 1200 series HPLC system with an Agilent G1315D DAD detector (methods). All compounds subjected to biological testing were >95% pure by HPLC analysis.

### Methyl (*S*)-5-Oxopyrrolidine-2-carboxylate (**2a**)

Compound **2a** was synthesized according to the published procedure,^42^ and the ^1^H NMR spectrum aligned with published data.

### Ethyl (*S*)-5-Oxopyrrolidine-2-carboxylate (**2b**)

Compound **2b** was synthesized according to the published procedure,^43^ and the ^1^H NMR spectrum aligned with published data.

### General Method for Synthesis of Esters **2c** and **2d**

l-Pyroglutamic acid **2** (2.00 g, 15.5 mmol, 1 equiv) was dissolved in anhydrous DCM (30 mL) and the corresponding alcohol (46.5 mmol, 3 equiv) was added, followed by DMAP (94.6 mg, 0.775 mmol, 0.05 equiv) and DCC (3.52 g, 17.0 mmol, 1.1 equiv). The resulting mixture was stirred at rt under nitrogen atmosphere for 20 h. The precipitate (DCU) was filtered off and volatiles were removed under reduced pressure. The residue was redissolved in a small amount of cold EtOAc (10 mL), and the remaining precipitate was removed by a second filtration. Solvent was evaporated and the crude material was purified by LC on silica gel (DCM/MeOH, 30:1) to afford desired products **2c** and **2d**.

#### Cyclopropyl (*S*)-5-Oxopyrrolidine-2-carboxylate (**2c**)

Cyclopropanol (2.70 g, 3.03 mL). Product **2c** was isolated as a colorless oil (2.40 g) in 92% yield. ^1^H NMR (500 MHz, CDCl_3_): δ 0.67–0.78 (m, 4H), 2.12–2.22 (m, 1H), 2.26–2.49 (m, 3H), 4.09–4.32 (m, 2H), 6.68 (bs, 1H). ESI MS: 170.1 ([M + H]^+^).

#### Cyclopropylmethyl (*S*)-5-Oxopyrrolidine-2-carboxylate (**2d**)

Cyclopropylmethanol (3.35 g, 3.76 mL). Product **2d** was isolated as a colorless oil (2.51 g) in 88% yield. ^1^H NMR (500 MHz, CDCl_3_): δ 0.21–0.37 (m, 2H), 0.49–0.65 (m, 2H), 1.06–1.21 (m, 1H), 2.20–2.28 (m, 1H), 2.30–2.46 (m, 2H), 2.46–2.54 (m, 1H), 3.99 (d, *J* = 7.4, 2H), 4.26 (ddd, *J* = 8.9, 5.1, 0.7, 1H), 6.26 (bs, 1H). ESI MS: 184.2 ([M + H]^+^).

### *tert*-Butyl (*S*)-5-Oxopyrrolidine-2-carboxylate (**2e**)

Compound **2e** was synthesized according to the published procedure,^44^ and the ^1^H NMR spectrum aligned with published data.

### General Method for Synthesis of Fmoc-Protected Compounds **3a**–**3e**

A solution of esters **2a**–**2e** (14.0 mmol, 1 equiv) in anhydrous THF (40 mL) was cooled to −78 °C under inert nitrogen atmosphere. LiHMDS (1 M in THF; 13.3 mL, 13.3 mmol, 0.95 equiv) was added dropwise during 10 min, and the mixture was stirred for an additional 15 min at the same temperature. Then it was transferred via cannula to a solution of Fmoc-Cl (4.34 g, 16.8 mmol, 1.2 equiv) in anhydrous THF (60 mL) cooled to −78 °C. The resulting mixture was stirred at −78 °C for 2 h and at rt for 16 h. The reaction was quenched with saturated NH_4_Cl (100 mL), and the aqueous phase was extracted with EtOAc (3 × 100 mL). Combined organic phases were washed with brine (2 × 100 mL) and dried over anhydrous MgSO_4_. Volatiles were evaporated *in vacuo*, and the residue was chromatographed on a Biotage Flash chromatography (CHCl_3_/0–100% EtOAc).

#### 1-((9*H*-Fluoren-9-yl)methyl) 2-Methyl (*S*)-5-Oxopyrrolidine-1,2-dicarboxylate (**3a**)

Starting material **2a** (2.00 g). Product **3a** was isolated as a colorless solid (4.85 g) in 95% yield. ^1^H NMR (500 MHz, CDCl_3_): δ 2.07–2.24 (m, 1H), 2.39 (ddt, *J* = 13.4, 10.7, 9.4, 1H), 2.57 (ddd, *J* = 17.6, 9.3, 3.1, 1H), 2.71 (ddd, *J* = 17.5, 10.8, 9.4, 1H), 3.74 (s, 3H), 4.30 (t, *J* = 7.2, 1H), 4.46 (dd, *J* = 10.6, 7.3, 1H), 4.58 (dd, *J* = 10.6, 7.2, 1H), 4.65 (dd, *J* = 9.5, 2.5, 1H), 7.28–7.38 (m, 2H), 7.41 (t, *J* = 7.4, 2H), 7.70 (d, *J* = 7.5, 1H), 7.75 (d, *J* = 7.5, 1H), 7.77 (d, *J* = 7.5, 2H). ESI MS: 366.1 ([M + H]^+^).

#### 1-((9*H*-Fluoren-9-yl)methyl) 2-Ethyl (*S*)-5-Oxopyrrolidine-1,2-dicarboxylate (**3b**)

Compound **3b** was synthesized according to the published procedure,^18^ and the ^1^H NMR spectrum aligned with published data.

#### 1-((9*H*-Fluoren-9-yl)methyl) 2-Cyclopropyl (*S*)-5-Oxopyrrolidine-1,2-dicarboxylate (**3c**)

Starting material **2c** (2.37 g). Product **3c** was isolated as a light-yellow solid (3.42 g) in 63% yield. ^1^H NMR (500 MHz, CDCl_3_): δ 0.66–0.79 (m, 4H), 2.09 (ddt, *J* = 13.4, 9.5, 3.0, 1H), 2.38 (ddt, *J* = 13.5, 10.6, 9.4, 1H), 2.57 (ddd, *J* = 17.6, 9.4, 3.2, 1H), 2.72 (ddd, *J* = 17.6, 10.6, 9.5, 1H), 4.17–4.26 (m, 1H), 4.31 (t, *J* = 7.4, 1H), 4.43 (dd, *J* = 10.6, 7.5, 1H), 4.49–4.68 (m, 2H), 7.33 (tdd, *J* = 7.5, 3.0, 1.2, 2H), 7.37–7.46 (m, 2H), 7.71 (dd, *J* = 7.5, 1.0, 1H), 7.74–7.79 (m, 3H). ESI MS: 392.2 ([M + H]^+^).

#### 1-((9*H*-Fluoren-9-yl)methyl) 2-Cyclopropylmethyl (*S*)-5-Oxopyrrolidine-1,2-dicarboxylate (**3d**)

Starting material **2d** (2.56 g). Product **3d** was isolated as a light-yellow solid (3.98 g) in 70% yield. ^1^H NMR (500 MHz, CDCl_3_): δ 0.27 (d, *J* = 4.8, 2H), 0.55 (d, *J* = 8.0, 2H), 1.03–1.21 (m, 1H), 1.81–1.90 (m, 1H), 2.08–2.24 (m, 1H), 2.35–2.52 (m, 1H), 2.58 (ddd, *J* = 17.6, 9.3, 2.9, 1H), 2.73 (dt, *J* = 16.8, 10.0, 1H), 3.65–3.83 (m, 1H), 3.91–4.09 (m, 2H), 4.31 (t, *J* = 7.4, 1H), 4.44 (dd, *J* = 10.7, 7.4, 1H), 4.55 (dd, *J* = 10.5, 7.4, 1H), 4.70 (dd, *J* = 9.5, 2.5, 1H), 7.33 (t, *J* = 7.5, 2H), 7.41 (t, *J* = 7.5, 2H), 7.61–7.84 (m, 4H). ESI MS: 406.2 ([M + H]^+^).

#### 1-((9*H*-Fluoren-9-yl)methyl) 2-*tert*-Butyl (*S*)-5-Oxopyrrolidine-1,2-dicarboxylate (**3e**)

Compound **3e** was synthesized according to the published procedure,^45^ and the ^1^H NMR spectrum was in agreement with published data.

### General Method for Synthesis of Compounds **4a**–**4e**

Trimethylsilyl diazomethane (2 M solution in hexanes; 6.0 mL, 12.0 mmol, 1.2 equiv) was dissolved in anhydrous THF (75 mL), the reaction mixture was cooled to −98 °C, *n*-BuLi (2.5 M in hexanes; 4.9 mL, 12.3 mmol, 1.23 equiv) was added dropwise during 15 min, and the resulting yellow mixture was stirred for 30 min at the same temperature. This solution was transferred via cannula to a solution of compounds **3a**–**3e** (10 mmol, 1 equiv) in anhydrous THF (100 mL) during 30 min under inert atmosphere at −98 °C. The resulting mixture was stirred for 30 min at the same temperature and then was allowed to heat to −78 °C and stirred for a further 2 h. The mixture was then quenched with saturated NH_4_Cl (50 mL) and water (50 mL). Phases were separated, the water phase was extracted with EtOAc (2 × 150 mL), and combined organic layers were washed with brine (100 mL) and dried over anhydrous MgSO_4_. Volatiles were removed under reduced pressure, and the solid residue was purified by LC on silica gel (cyclohexane/EtOAc, 2:1 to 1:1).

#### Methyl (*S*)-2-((((9*H*-Fluoren-9-yl)methoxy)carbonyl)amino)-6-diazo-5-oxohexanoate (**4a**)

Starting material **3a** (3.65 g). Product **4a** was isolated as a light-yellow solid (2.65 g) in 65% yield. ^1^H NMR (500 MHz, CDCl_3_): δ 1.92–2.01 (m, 1H), 2.16–2.32 (m, 1H), 2.31–2.54 (m, 2H), 3.76 (s, 3H), 4.23 (t, *J* = 7.1, 1H), 4.34–4.45 (m, 3H), 5.26 (bs, 1H), 5.53 (d, *J* = 8.1, 1H), 7.28–7.37 (m, 2H), 7.41 (dd, *J* = 8.4, 6.6, 2H), 7.60 (t, *J* = 7.2, 2H), 7.77 (d, *J* = 7.5, 2H). ESI MS: 430.1 ([M + Na]^+^).

#### Ethyl (*S*)-2-((((9*H*-Fluoren-9-yl)methoxy)carbonyl)amino)-6-diazo-5-oxohexanoate (**4b**)

Compound **4b** was synthesized according to the published procedure,^18^ and the ^1^H NMR spectrum was in agreement with published data.

#### Cyclopropyl (*S*)-2-((((9*H*-Fluoren-9-yl)methoxy)carbonyl)amino)-6-diazo-5-oxohexanoate (**4c**)

Starting material **3c** (3.91 g). Product **4c** was isolated as a light-yellow solid (2.05 g) in 47% yield. ^1^H NMR (500 MHz, CDCl_3_): δ 0.60–0.85 (m, 4H), 1.84–2.09 (m, 1H), 2.20 (s, 1H), 2.28–2.53 (m, 2H), 4.15–4.26 (m, 2H), 4.27–4.48 (m, 2H), 5.27 (bs, 1H), 5.51 (d, *J* = 8.2, 1H), 7.32 (t, *J* = 7.4, 2H), 7.41 (t, *J* = 7.5, 2H), 7.60 (t, *J* = 6.6, 2H), 7.77 (d, *J* = 7.6, 2H). ESI MS: 456.2 ([M + Na]^+^).

#### Cyclopropylmethyl (*S*)-2-((((9*H*-Fluoren-9-yl)methoxy)carbonyl)amino)-6-diazo-5-oxohexanoate (**4d**)

Starting material **3d** (4.05 g). Product **4d** was isolated as a light-yellow solid (2.31 g) in 52% yield. ^1^H NMR (500 MHz, CDCl_3_): δ 0.29 (d, *J* = 5.2, 2H), 0.58 (d, *J* = 7.8, 2H), 1.98–2.11 (m, 1H), 2.18–2.32 (m, 1H), 2.33–2.55 (m, 2H), 3.99 (dd, *J* = 7.4, 4.0, 2H), 4.23 (t, *J* = 7.0, 1H), 4.34–4.46 (m, 3H), 5.27 (bs, 1H), 5.53 (d, *J* = 8.1, 1H), 7.32 (t, *J* = 7.4, 2H), 7.41 (t, *J* = 7.5, 2H), 7.60 (t, *J* = 7.1, 2H), 7.77 (d, *J* = 7.5, 2H). ESI MS: 470.2 ([M + Na]^+^).

#### *tert*-Butyl (*S*)-2-((((9*H*-Fluoren-9-yl)methoxy)carbonyl)amino)-6-diazo-5-oxohexanoate (**4e**)

Starting material **3e** (4.07 g). Product **4e** was isolated as a light-yellow solid (3.03 g) in 67% yield. ^1^H NMR (401 MHz, CDCl_3_): δ 1.50 (s, 9H), 1.94–2.07 (m, 1H), 2.18–2.29 (m, 1H), 2.31–2.53 (m, 2H), 4.21–4.32 (m, 2H), 4.41 (d, *J* = 7.1, 2H), 5.29 (bs, 1H), 5.50 (d, *J* = 8.1, 1H), 7.34 (tt, *J* = 7.4, 1.4, 2H), 7.43 (t, *J* = 7.4, 2H), 7.63 (dd, *J* = 7.7, 4.0, 2H), 7.79 (d, *J* = 7.3, 2H). ESI MS: 472.2 ([M + Na]^+^).

### General Method for Synthesis of Compounds **5a**–**5e**

Compounds **4a**–**4e** (4.00 mmol, 1 equiv) were dissolved in anhydrous DCM (18 mL), and piperidine (1.70 g, 1.98 mL, 20.0 mmol, 5 equiv) was added. The reaction mixture was stirred at rt under an inert atmosphere for 2–3.5 h. Volatiles were evaporated, and the residue was purified by LC on silica gel (DCM/MeOH, 30:1).

#### Methyl (*S*)-2-Amino-6-diazo-5-oxohexanoate (**5a**)

Starting material **4a** (1.63 g); reaction time 2 h. Product **5a** was isolated as a light-yellow oil (532 mg) in 72% yield. ^1^H NMR (401 MHz, CDCl_3_): δ 1.53 (bs, 2H), 1.76–1.87 (m, 1H), 2.03–2.15 (m, 1H), 2.42–2.55 (m, 2H), 3.44 (dd, *J* = 8.3, 5.1, 1H), 3.64 (s, 3H), 5.26 (bs, 1H). ESI MS: 186.1 ([M + H]^+^).

#### Ethyl (*S*)-2-Amino-6-diazo-5-oxohexanoate (**5b**)

Compound **5b** was synthesized according to the published procedure,^18^ and the ^1^H NMR spectrum was in agreement with published data.

#### Cyclopropyl (*S*)-2-Amino-6-diazo-5-oxohexanoate (**5c**)

Starting material **4c** (1.73 g); reaction time 3 h. Product **5c** was isolated as a light-yellow oil (567 mg) in 67% yield. ^1^H NMR (401 MHz, CDCl_3_): δ 0.63–0.78 (m, 4H), 1.46–1.54 (m, 2H), 1.80 (dq, *J* = 14.5, 7.4, 1H), 2.02–2.14 (m, 1H), 2.41–2.53 (m, 2H), 3.39–3.42 (m, 1H), 4.14–4.18 (m, 1H), 5.27 (bs, 1H). ESI MS: 234.1 ([M + Na]^+^).

#### Cyclopropylmethyl (*S*)-2-Amino-6-diazo-5-oxohexanoate (**5d**)

Starting material **4d** (1.79 g); reaction time 3 h. Product **5d** was isolated as a light-yellow oil (443 mg) in a 49% yield. ^1^H NMR (401 MHz, CDCl_3_): δ 0.23–0.35 (m, 2H), 0.54–0.64 (m, 2H), 1.09–1.20 (m, 1H), 1. 52–1.61 (m, 2H), 2.10–2.21 (m, 1H), 2.30–2.40 (m, 1H), 2.55–2.77 (m, 2H), 3.95–4.08 (m, 3H), 5.54 (bs, 1H). ESI MS: 226.1 ([M + H]^+^).

#### *tert*-Butyl (*S*)-2-Amino-6-diazo-5-oxohexanoate (**5e**)

Starting material **4e** (1.80 g); reaction time 3.5 h. Product **5e** was isolated as a light-yellow oil (611 mg) in 67% yield. ^1^H NMR (401 MHz, CDCl_3_): δ 1.43 (s, 9H), 1.58 (bs, 2H), 1.72–1.81 (m, 1H), 1.99–2.09 (m, 1H), 2.38–2.50 (m, 2H), 3.30 (dd, *J* = 8.3, 5.0, 1H), 5.27 (bs, 1H). ESI MS: 228.1 ([M + H]^+^).

### General Method for Synthesis of Compounds **6a**–**6e**

Fmoc-l-Trp-OH (938 mg, 2.20 mmol, 1.1 equiv) and HATU (913 mg, 2.40 mmol, 1.2 equiv) were suspended in anhydrous DCM (20 mL) under inert atmosphere, and the reaction mixture was cooled to 0 °C. DIPEA (775 mg, 1.05 mL, 6.00 mmol, 3 equiv) was added, and the mixture was stirred for 5 min. Finally, a solution of amines **5a**, **5c**, and **5e** (2.00 mmol, 1 equiv) in anhydrous DCM (10 mL) was slowly added over 5 min. The resulting mixture was stirred for 30 min at 0 °C and then for 2 h at rt. DCM (50 mL) was added, and the organic phase was washed with saturated NaHCO_3_ (50 mL), distilled H_2_O (50 mL), 10% KHSO_4_ (50 mL), distilled H_2_O (50 mL), and saturated NaCl (50 mL) and dried over anhydrous MgSO_4_. DCM was evaporated *in vacuo*, and the residue was purified by LC on silica gel (DCM/EtOAc, 3:1).

#### Methyl (*S*)-2-((*S*)-2-((((9*H*-Fluoren-9-yl)methoxy)carbonyl)amino)-3-(1*H*-indol-3-yl)propanamido)-6-diazo-5-oxohexanoate (**6a**)

Starting material **5a** (370 mg). Product **6a** was isolated as a light-yellow solid (1.01 g) with 85% yield. ^1^H NMR (401 MHz, CDCl_3_): δ 1.85–1.97 (m, 1H), 2.08–2.18 (m, 2H), 2.18–2.28 (m, 1H), 3.19 (dd, *J* = 14.5, 7.4, 1H), 3.41 (d, *J* = 8.6, 1H), 3.67 (s, 3H), 4.24 (t, *J* = 7.1, 1H), 4.34–4.51 (m, 3H), 4.52–4.60 (m, 1H), 5.08 (bs, 1H), 5.50 (d, *J* = 7.8, 1H), 6.57 (d, *J* = 7.2, 1H), 7.10 (bs, 1H), 7.16 (t, *J* = 7.4, 1H), 7.23 (ddd, *J* = 8.2, 7.0, 1.2, 1H), 7.33 (tdd, *J* = 7.5, 2.4, 1.1, 2H), 7.37–7.40 (m, 1H), 7.41–7.46 (m, 2H), 7.59 (dd, *J* = 7.5, 5.0, 2H), 7.70 (d, *J* = 7.8, 1H), 7.79 (d, *J* = 7.5, 2H), 8.19 (bs, 1H). ESI MS: 616.2 ([M + Na]^+^).

#### Ethyl (*S*)-2-((*S*)-2-((((9*H*-Fluoren-9-yl)methoxy)carbonyl)amino)-3-(1*H*-indol-3-yl)propanamido)-6-diazo-5-oxohexanoate (**6b**)

Compound **6b** was synthesized according to the published procedure,^18^ and the ^1^H NMR spectrum was in agreement with published data.

#### Cyclopropyl (*S*)-2-((*S*)-2-((((9*H*-Fluoren-9-yl)methoxy)carbonyl)amino)-3-(1*H*-indol-3-yl)propanamido)-6-diazo-5-oxohexanoate (**6c**)

Starting material **5c** (422 mg). Product **6c** was isolated as a light-yellow solid (903 mg) in a 73% yield. ^1^H NMR (500 MHz, CDCl_3_): δ 0.58–0.76 (m, 4H), 1.75–1.95 (m, 1H), 1.96–2.27 (m, 3H), 3.17 (dd, *J* = 14.5, 7.3, 1H), 3.33–3.52 (m, 1H), 4.09 (s, 1H), 4.21 (t, *J* = 7.1, 1H), 4.29–4.63 (m, 4H), 5.05 (s, 1H), 5.46 (bs, 1H), 7.09 (bs, 1H), 7.15 (t, *J* = 7.5, 1H), 7.21 (t, *J* = 7.5, 1H), 7.28–7.34 (m, 2H), 7.35–7.46 (m, 3H), 7.57 (t, *J* = 7.0, 2H), 7.69 (d, *J* = 8.0, 1H), 7.77 (d, *J* = 7.6, 2H), 8.15 (bs, 1H). ESI MS: 642.2 ([M + Na]^+^).

#### Cyclopropylmethyl (*S*)-2-((*S*)-2-((((9*H*-Fluoren-9-yl)methoxy)carbonyl)amino)-3-(1*H*-indol-3-yl)propanamido)-6-diazo-5-oxohexanoate (**6d**)

Starting material **5d** (451 mg). Product **6d** was isolated as a light-yellow solid (1.20 g) in 95% yield. ^1^H NMR (500 MHz, CDCl_3_): δ 0.26 (s, 2H), 0.56 (d, *J* = 8.1, 2H), 1.08 (s, 1H), 1.83–2.02 (m, 1H), 2.02–2.37 (m, 3H), 3.17 (dd, *J* = 14.6, 7.3, 1H), 3.28–3.54 (m, 1H), 3.77–4.01 (m, 2H), 4.21 (t, *J* = 7.1, 1H), 4.27–4.42 (m, 1H), 4.41–4.50 (m, 2H), 4.50–4.61 (m, 1H), 5.07 (s, 1H), 5.46 (bs, 1H), 6.53 (bs, 1H), 7.08 (bs, 1H), 7.14 (t, *J* = 7.5, 1H), 7.20 (t, *J* = 7.5, 1H), 7.31 (td, *J* = 7.4, 3.2, 2H), 7.37 (d, *J* = 8.1, 1H), 7.40 (t, *J* = 7.4, 2H), 7.57 (t, *J* = 7.3, 2H), 7.68 (d, *J* = 7.9, 1H), 7.77 (d, *J* = 7.6, 2H), 8.15 (bs, 1H). ESI MS: 656.3 ([M + Na]^+^).

#### *tert*-Butyl (*S*)-2-((*S*)-2-((((9*H*-Fluoren-9-yl)methoxy)carbonyl)amino)-3-(1*H*-indol-3-yl)propanamido)-6-diazo-5-oxohexanoate (**6e**)

Starting material **5e** (455 mg). Product **6e** was isolated as a light-yellow solid (1.07 g) with 84% yield. ^1^H NMR (401 MHz, *d*_6_-DMSO): δ 1.41 (s, 9H), 1.78–1.88 (m, 1H), 2.04–2.14 (m, 1H), 2.32–2.44 (m, 2H), 2.95 (dd, *J* = 14.7, 10.5, 1H), 3.12 (dd, *J* = 14.6, 4.0, 1H), 4.11–4.19 (m, 4H), 4.34 (ddd, *J* = 10.0, 8.3, 3.9, 1H), 6.02 (bs, 1H), 6.99 (t, *J* = 7.3, 1H), 7.07 (t, *J* = 7.2, 1H), 7.21 (d, *J* = 2.3, 1H), 7.25 (td, *J* = 7.5, 1.1, 1H), 7.30–7.36 (m, 2H), 7.36–7.45 (m, 2H), 7.53 (d, *J* = 8.5, 1H), 7.62 (d, *J* = 7.5, 1H), 7.66 (d, *J* = 7.4, 1H), 7.70 (d, *J* = 7.8, 1H), 7.88 (d, *J* = 7.5, 2H), 8.38 (d, *J* = 7.5, 1H), 10.83 (bs, 1H). ESI MS: 658.3 ([M + Na]^+^).

### General Method for Synthesis of Compounds **7a**–**7e**

Compounds **6a**–**6e** (1.50 mmol, 1 equiv) were dissolved in anhydrous DCM (10 mL), diethylamine (1.10 g, 1.55 mL, 15.0 mmol, 10 equiv) was added, and the reaction mixture was stirred at rt under inert atmosphere for 3 h. Volatiles were evaporated, and the residue was purified by LC on silica gel (DCM/MeOH, 30:1 to 20:1).

#### Methyl (*S*)-2-((*S*)-2-Amino-3-(1*H*-indol-3-yl)propanamido)-6-diazo-5-oxohexanoate (**7a**)

Starting material **6a** (890 mg). Product **7a** was isolated as a light-yellow solid (514 mg) in 92% yield. ^1^H NMR (500 MHz, CDCl_3_): δ 1.91–2.01 (m, 1H), 2.05–2.29 (m, 1H), 2.55–2.67 (m, 2H), 3.06 (dd, *J* = 14.4, 8.1, 1H), 3.30 (dd, *J* = 14.4, 4.2, 1H), 3.73 (s, 3H), 3.74–3.78 (m, 1H), 4.57 (td, *J* = 8.2, 4.2, 1H), 5.11 (bs, 1H), 7.06–7.15 (m, 2H), 7.21 (t, *J* = 7.6, 1H), 7.37 (d, *J* = 8.1, 1H), 7.69 (d, *J* = 7.9, 1H), 7.86 (d, *J* = 8.3, 1H), 8.20 (bs, 1H). ESI MS: 394.2 ([M + Na]^+^).

#### Ethyl (*S*)-2-((*S*)-2-Amino-3-(1*H*-indol-3-yl)propanamido)-6-diazo-5-oxohexanoate (**7b**)

Compound **7b** was synthesized according to the published procedure,^18^ and the ^1^H NMR spectrum was in agreement with published data.

#### Cyclopropyl (*S*)-2-((*S*)-2-Amino-3-(1*H*-indol-3-yl)propanamido)-6-diazo-5-oxohexanoate (**7c**)

Starting material **6c** (930 mg). Product **7c** was isolated as a light-yellow solid (536 mg) in a 90% yield. ^1^H NMR (500 MHz, CDCl_3_): δ 0.57–0.89 (m, 4H), 1.32–1.50 (m, 2H), 1.86–1.99 (m, 1H), 2.03–2.28 (m, 3H), 3.04 (dd, *J* = 14.4, 8.1, 1H), 3.30 (dd, *J* = 14.4, 4.2, 1H), 3.75 (dd, *J* = 8.1, 4.2, 1H), 4.11–4.19 (m, 1H), 4.51 (td, *J* = 8.4, 4.2, 1H), 5.10 (bs, 1H), 6.99–7.17 (m, 2H), 7.21 (ddd, *J* = 8.2, 7.1, 1.2, 1H), 7.37 (d, *J* = 8.2, 1H), 7.69 (d, *J* = 7.9, 1H), 7.87 (d, *J* = 8.3, 1H), 8.15 (bs, 1H). ESI MS: 420.2 ([M + Na]^+^).

#### Cyclopropylmethyl (*S*)-2-((*S*)-2-Amino-3-(1*H*-indol-3-yl)propanamido)-6-diazo-5-oxohexanoate (**7d**)

Starting material **6d** (951 mg). Product **7d** was isolated as a light-yellow solid (586 mg) in 95% yield. ^1^H NMR (401 MHz, CDCl_3_): δ 0.21–0.35 (m, 2H), 0.50–0.64 (m, 2H), 0.95–1.21 (m, 1H), 1.85–2.06 (m, 1H), 2.06–2.36 (m, 3H), 3.05 (dd, *J* = 14.4, 8.1, 1H), 3.30 (dd, *J* = 14.4, 4.2, 1H), 3.74 (dd, *J* = 8.1, 4.1, 1H), 3.95 (dd, *J* = 7.4, 1.5, 2H), 4.58 (td, *J* = 8.2, 3.7, 1H), 5.12 (bs, 1H), 7.06–7.15 (m, 2H), 7.19 (t, *J* = 7.5, 1H), 7.37 (d, *J* = 8.0, 1H), 7.68 (d, *J* = 7.9, 1H), 7.91 (d, *J* = 8.4, 1H), 8.42 (bs, 1H). ESI MS: 412.2 ([M + H]^+^).

#### *tert*-Butyl (*S*)-2-((*S*)-2-Amino-3-(1*H*-indol-3-yl)propanamido)-6-diazo-5-oxohexanoate (**7e**)

Starting material **6e** (954 mg). Product **7e** was isolated as a light-yellow solid (579 mg) in a 93% yield. ^1^H NMR (401 MHz, CDCl_3_): δ 1.48 (s, 9H), 1.88–2.00 (m, 1H), 2.01–2.28 (m, 5H), 3.07 (dd, *J* = 14.5, 8.1, 1H), 3.33 (dd, *J* = 14.5, 3.9, 1H), 3.79 (dd, *J* = 8.2, 4.1, 1H), 4.47 (td, *J* = 8.3, 4.2, 1H), 5.16 (bs, 1H), 7.09–7.16 (m, 2H), 7.21 (ddd, *J* = 8.1, 7.0, 1.2, 1H), 7.38 (dt, *J* = 8.1, 0.9, 1H), 7.69 (dd, *J* = 7.9, 1.0, 1H), 7.92 (d, *J* = 8.2, 1H), 8.51 (bs, 1H). ESI MS: 414.2 ([M + H]^+^).

### General Method for Synthesis of Prodrugs **P1**–**P5**

To the solution of amines **7a**–**7e** (0.300 mmol, 1 equiv) in anhydrous DMF (2 mL), pyridine (48 mg, 48 μL, 0.600 mmol, 2 equiv) was added followed by acetic anhydride (34 mg, 31 μL, 0.330 mmol, 1.1 equiv) at rt under inert atmosphere. The mixture was stirred at the same temperature for 2 h. Then, volatiles were removed under reduced pressure, and the residue was purified by LC on silica gel (DCM/MeOH, 30:1).

#### Methyl (*S*)-2-((*S*)-2-Acetamido-3-(1*H*-indol-3-yl)propanamido)-6-diazo-5-oxohexanoate (**P1**)

Starting material **7a** (111 mg). Product **P1** was isolated as a light-yellow solid (93 mg) in 75% yield. ^1^H NMR (401 MHz, *d*_6_-DMSO): δ 1.76 (s, 3H), 1.77–1.91 (m, 1H), 1.92–2.07 (m, 1H), 2.37 (s, 2H), 2.81–2.95 (m, 1H), 3.00–3.15 (m, 1H), 3.61 (s, 3H), 4.19–4.34 (m, 1H), 4.47–4.62 (m, 1H), 6.03 (bs, 1H), 6.98 (t, *J* = 7.5, 1H), 7.06 (t, *J* = 7.5, 1H), 7.14 (s, 1H), 7.32 (d, *J* = 8.1, 1H), 7.62 (d, *J* = 7.9, 1H), 8.07 (d, *J* = 8.1, 1H), 8.46 (d, *J* = 7.6, 1H), 10.82 (bs, 1H). ^13^C NMR (101 MHz, *d*_6_-DMSO): δ 23.01, 26.24, 28.14, 51.71, 52.38, 53.59, 110.50, 111.72, 118.62, 118.94, 121.29, 124.09, 127.75, 136.48, 169.60, 172.56, 172.62, 194.63. ESI MS: 436.2 ([M + Na]^+^). HR ESI MS: calcd for C_20_H_23_N_5_NaO_5_ 436.1592; found 436.1591.

#### Ethyl (*S*)-2-((*S*)-2-Acetamido-3-(1*H*-indol-3-yl)propanamido)-6-diazo-5-oxohexanoate (**P2**)

Starting material **7b** (116 mg). Product **P2** was isolated as a light-yellow solid (85 mg) in 66% yield. ^1^H NMR (401 MHz, *d*_6_-DMSO): δ 1.17 (t, *J* = 7.1, 3H), 1.66–1.91 (m, 4H), 1.91–2.11 (m, 1H), 2.26–2.45 (m, 2H), 2.87 (dd, *J* = 14.7, 9.6, 1H), 3.08 (dd, *J* = 14.7, 4.5, 1H), 4.07 (td, *J* = 7.2, 6.2, 2H), 4.23 (ddd, *J* = 9.3, 7.4, 5.1, 1H), 4.46–4.65 (m, 1H), 6.04 (bs, 1H), 6.98 (ddd, *J* = 7.9, 7.0, 1.1, 1H), 7.06 (ddd, *J* = 8.1, 6.9, 1.2, 1H), 7.14 (d, *J* = 2.3, 1H), 7.31 (d, *J* = 7.8, 1H), 7.62 (d, *J* = 7.8, 1H), 8.06 (d, *J* = 8.1, 1H), 8.45 (d, *J* = 7.6, 1H), 10.82 (bs, 1H). ^13^C NMR (101 MHz, *d*_6_-DMSO): δ 14.49, 23.00, 26.26, 28.18, 51.84, 53.56, 61.01, 110.56, 111.73, 118.62, 118.92, 121.30, 124.07, 127.74, 136.49, 169.58, 172.05, 172.66. ESI MS: 450.2 ([M + Na]^+^). HR ESI MS: calcd for C_21_H_25_N_5_NaO_5_ 450.1748; found 450.1750.

#### Cyclopropyl (*S*)-2-((*S*)-2-Acetamido-3-(1*H*-indol-3-yl)propanamido)-6-diazo-5-oxohexanoate (**P3**)

Starting material **7c** (119 mg). Product **P3** was isolated as a light-yellow solid (108 mg) with 82% yield. ^1^H NMR (401 MHz, *d*_6_-DMSO): δ 0.45–0.94 (m, 4H), 1.58–1.90 (m, 4H), 1.86–2.09 (m, 1H), 2.15–2.44 (m, 2H), 2.85 (dd, *J* = 14.6, 9.7, 1H), 2.95–3.17 (m, 1H), 3.95–4.14 (m, 1H), 4.14–4.28 (m, 1H), 4.53 (s, 1H), 6.04 (bs, 1H), 6.98 (t, *J* = 7.5, 1H), 7.06 (t, *J* = 7.5, 1H), 7.14 (bs, 1H), 7.32 (d, *J* = 8.0, 1H), 7.62 (d, *J* = 7.8, 1H), 8.05 (d, *J* = 7.9, 1H), 8.47 (d, *J* = 7.4, 1H), 10.82 (bs, 1H). ^13^C NMR (101 MHz, *d*_6_-DMSO): δ 4.59, 4.62, 22.36, 25.37, 27.55, 48.98, 51.16, 52.89, 111.13, 118.01, 118.28, 120.70, 123.45, 168.97, 172.08, 172.29, 193.98. ESI MS: 462.2 ([M + Na]^+^). HR ESI MS: calcd for C_22_H_25_N_5_NaO_5_ 462.1748; found 462.1748.

#### Cyclopropylmethyl (*S*)-2-((*S*)-2-Acetamido-3-(1*H*-indol-3-yl)propanamido)-6-diazo-5-oxohexanoate (**P4**)

Starting material **7d** (123 mg). Product **P4** was isolated as a light-yellow solid (101 mg) in 74% yield. ^1^H NMR (500 MHz, CDCl_3_): δ 0.27 (s, 2H), 0.57 (d, *J* = 8.0, 2H), 1.04–1.16 (m, 1H), 1.88–1.98 (m, 1H), 2.00 (s, 3H), 2.06–2.22 (m, 2H), 2.28 (s, 1H), 3.16 (dd, *J* = 14.6, 7.5, 1H), 3.35 (dd, *J* = 14.5, 5.1, 1H), 3.83–3.95 (m, 2H), 4.44 (q, *J* = 7.6, 5.1, 1H), 4.74 (q, *J* = 7.0, 1H), 5.17 (bs, 1H), 6.16 (d, *J* = 7.6, 1H), 6.43–6.56 (m, 1H), 7.09–7.17 (m, 2H), 7.20 (t, *J* = 7.6, 1H), 7.36 (d, *J* = 8.1, 1H), 7.69 (d, *J* = 7.9, 1H), 8.15 (bs, 1H). ^13^C NMR (101 MHz, CDCl_3_): δ 3.45, 3.50, 9.76, 23.44, 27.06, 28.31, 36.26, 52.20, 54.06, 70.56, 110.41, 111.39, 118.84, 119.89, 122.33, 123.61, 127.72, 136.31, 170.24, 171.43, 171.50, 194.08. ESI MS: 476.2 ([M + Na]^+^). HR ESI MS: calcd for C_23_H_27_N_5_NaO_5_ 476.1905; found 476.1902.

#### *tert*-Butyl (*S*)-2-((*S*)-2-Acetamido-3-(1*H*-indol-3-yl)propanamido)-6-diazo-5-oxohexanoate (**P5**)

Starting material **7e** (124 mg). Product **P5** was isolated as a light-yellow solid (126 mg) in 92% yield. ^1^H NMR (401 MHz, CDCl_3_): δ 1.42 (s, 9H), 1.81–1.92 (m, 1H), 1.94 (s, 3H), 2.01–2.17 (m, 2H), 2.18–2.30 (m, 1H), 3.19 (dd, *J* = 14.7, 6.6, 1H), 3.29 (dd, *J* = 14.7, 5.7, 1H), 4.31 (td, *J* = 7.7, 4.5, 1H), 4.77 (q, *J* = 6.4, 1H), 5.16 (bs, 1H), 6.35 (d, *J* = 7.7, 1H), 6.82 (d, *J* = 7.4, 1H), 7.04–7.12 (m, 2H), 7.16 (ddd, *J* = 8.2, 7.0, 1.3, 1H), 7.33 (dt, *J* = 8.1, 1.0, 1H), 7.63 (d, *J* = 7.9, 1H), 8.56 (bs, 1H). ^13^C NMR (101 MHz, CDCl_3_): δ 23.33, 27.11, 28.04 (3C), 28.16, 36.29, 52.70, 53.99, 54.92, 82.49, 110.30, 111.45, 118.75, 119.76, 122.25, 123.57, 127.72, 136.35, 170.34, 170.48, 171.51, 194.22. ESI MS: 478.2 ([M + Na]^+^). HR ESI MS: calcd for C_23_H_29_O_5_NaN_5_ 478.20609; found 478.20567.

### Synthesis of Prodrug **P6**

Starting material **P2** (100 mg, 0.234 mmol, 1 equiv) was dissolved in a solution of 2 M methylamine in MeOH (6 mL), and the reaction mixture was heated to 60 °C for 20 h. Volatiles were evaporated, and the residue was purified by LC on silica gel (DCM/MeOH, 10:1 + 1% Et_3_N). Compound **P6** was obtained as a light-yellow solid (63 mg) in 65% yield.

#### (*S*)-2-((*S*)-2-Acetamido-3-(1*H*-indol-3-yl)propanamido)-6-diazo-*N*-methyl-5-oxohexanamide (**P6**)

^1^H NMR (401 MHz, *d*_6_-DMSO): δ 1.66–1.77 (m, 1H), 1.81 (s, 3H), 1.88–2.01 (m, 1H), 2.20–2.31 (m, 2H), 2.53 (d, *J* = 4.6, 3H), 2.92 (dd, *J* = 14.6, 8.7, 1H), 3.12 (dd, *J* = 14.6, 5.2, 1H), 4.15 (td, *J* = 8.6, 5.2, 1H), 4.48–4.52 (m, 1H), 5.97 (bs, 1H), 6.98 (t, *J* = 7.3, 1H), 7.06 (t, *J* = 7.1, 1H), 7.16 (d, *J* = 2.1, 1H), 7.33 (d, *J* = 8.0, 1H), 7.45 (d, *J* = 4.5, 1H), 7.59 (d, *J* = 7.7, 1H), 8.03–8.09 (m, 1H), 8.22 (d, *J* = 7.0, 1H), 10.83 (d, *J* = 2.7, 1H). ^13^C NMR (101 MHz, *d*_6_-DMSO): δ 22.59, 25.59, 27.04, 27.46, 36.40, 51.98, 53.70, 54.44, 110.05, 111.30, 118.23, 118.52, 120.89, 123.66, 127.31, 136.05, 169.50, 171.24, 171.75, 194.34. ESI MS: 435.2 ([M + Na]^+^). HR ESI MS: calcd for C_20_H_24_O_4_NaN_6_ 435.17512; found 435.17489.

### General Method for Synthesis of Prodrugs **P7**–**P9**

Appropriate carboxylic acid (0.266 mmol, 1.1 equiv) and HATU (106 mg, 0.278 mmol, 1.15 equiv) were dissolved in anhydrous DMF (4 mL), and the reaction mixture was cooled to 0 °C. DIPEA (125 mg, 168 μL, 0.967 mmol, 4 equiv) was added, and the mixture was stirred for 5 min. Finally, a solution of compound **7e** (100 mg, 0.242 mmol, 1 equiv) in anhydrous DMF (2 mL) was added over 5 min. The resulting mixture was stirred for 30 min at 0 °C and then at rt for 2 h. DMF was evaporated, EtOAc (100 mL) was added, and the organic phase was washed with saturated NaHCO_3_ (50 mL), distilled H_2_O (50 mL), and saturated NaCl (50 mL) and was dried over anhydrous MgSO_4_. The organic solvent was evaporated *in vacuo*. The residue was purified by LC on silica gel (various mobile phases) to afford the desired products.

#### *tert*-Butyl (*S*)-2-((*S*)-3-(1*H*-Indol-3-yl)-2-(2-morpholinoacetamido)propanamido)-6-diazo-5-oxohexanoate (**P7**)

Morpholinoacetic acid hydrochloride (48.3 mg); mobile phase: DCM/MeOH, 20:1. Product **P7** was isolated as a light-yellow solid (107 mg) in 82% yield. ^1^H NMR (401 MHz, CDCl_3_): δ 1.44 (s, 9H), 1.86–2.02 (m, 1H), 2.08–2.40 (m, 7H), 2.85 (d, *J* = 16.4, 1H), 2.98 (d, *J* = 16.4, 1H), 3.29 (t, *J* = 7.1, 2H), 3.38 (dtd, *J* = 14.0, 8.0, 6.6, 3.0, 4H), 4.38 (td, *J* = 7.5, 4.6, 1H), 4.77 (q, *J* = 6.8, 1H), 5.23 (bs, 1H), 6.84 (d, *J* = 7.4, 1H), 7.07–7.13 (m, 2H), 7.17 (ddd, *J* = 8.1, 7.0, 1.2, 1H), 7.34 (dt, *J* = 8.1, 1.0, 1H), 7.58 (d, *J* = 7.8, 1H), 7.63 (dd, *J* = 8.0, 1.1, 1H), 8.45 (bs, 1H). ^13^C NMR (101 MHz, CDCl_3_): δ 27.29, 27.65, 28.09 (3C), 36.33, 52.60, 53.44, 53.66 (2C), 54.90, 61.88, 66.80 (2C), 82.57, 110.35, 111.43, 118.76, 119.87, 122.41, 123.34, 127.71, 136.30, 170.50, 170.57, 171.39, 194.09. ESI MS: 541.3 ([M + H]^+^). HR ESI MS: calcd for C_27_H_37_O_6_N_6_ 541.27691; found 541.27637.

#### *tert*-Butyl (*S*)-2-((*S*)-3-(1*H*-Indol-3-yl)-2-(quinuclidine-4-carboxamido) propanamido)-6-diazo-5-oxohexanoate (**P8**)

Quinuclidine-4-carboxylic acid hydrochloride (51.0 mg); mobile phase: DCM/MeOH, 5:1 + 1% Et_3_N. Product **P8** was isolated as a light-yellow solid (119 mg) in 89% yield. ^1^H NMR (401 MHz, CDCl_3_): δ 1.42 (s, 9H), 1.55–1.67 (m, 6H), 1.82–1.94 (m, 1H), 2.00–2.17 (m, 2H), 2.17–2.32 (m, 1H), 2.82–2.89 (m, 6H), 3.17 (dd, *J* = 14.7, 6.8, 1H), 3.33 (dd, *J* = 14.7, 6.8, 1H), 4.29 (td, *J* = 7.5, 4.4, 1H), 4.74 (td, *J* = 7.0, 5.6, 1H), 5.17 (bs, 1H), 6.24 (d, *J* = 7.4, 1H), 6.72 (d, *J* = 7.2, 1H), 7.07–7.13 (m, 2H), 7.17 (ddd, *J* = 8.1, 6.9, 1.2, 1H), 7.35 (d, *J* = 8.1, 1H), 7.66 (d, *J* = 7.8, 1H), 8.69 (bs, 1H). ^13^C NMR (101 MHz, CDCl_3_): δ 27.03, 28.07 (3C), 28.12 (3C), 29.81, 36.30, 45.97, 47.29 (3C), 52.78, 53.64, 54.85, 82.49, 110.29, 111.47, 119.01, 119.75, 122.37, 123.61, 127.66, 136.40, 170.46, 171.43, 176.21, 194.13. ESI MS: 551.3 ([M + Na]^+^). HR ESI MS: calcd for C_29_H_39_O_5_N_6_ 551.29764; found 551.29730.

#### *tert*-Butyl (*S*)-6-Diazo-2-((*S*)-2-(2-(dimethylamino)acetamido)-3-(1*H*-indol-3-yl)propanamido)-5-oxohexanoate (**P9**)

Dimethylglycine (27.4 mg); mobile phase: DCM/MeOH, 15:1. Product **P9** was isolated as a light-yellow solid (106 mg) in 88% yield. ^1^H NMR (401 MHz, CDCl_3_): δ 1.40 (s, 9H), 1.84–1.94 (m, 1H), 2.02–2.12 (m, 1H), 2.11 (s, 6H), 2.17–2.30 (m, 2H), 2.87 (d, *J* = 16.1, 1H), 2.96 (d, *J* = 16.1, 1H), 3.26 (d, *J* = 6.9, 2H), 4.32 (q, *J* = 7.2, 1H), 4.76 (q, *J* = 6.9, 1H), 5.21 (bs, 1H), 7.01–7.17 (m, 4H), 7.32 (d, *J* = 8.1, 1H), 7.61 (d, *J* = 7.8, 1H), 7.85 (d, *J* = 8.0, 1H), 8.93 (bs, 1H). ^13^C NMR (101 MHz, CDCl_3_): δ 27.08, 27.98 (3C), 29.71, 36.32, 45.50 (2C), 52.64, 53.82, 54.83, 62.37, 82.31, 110.29, 111.43, 118.71, 119.43, 122.01, 123.45, 127.53, 136.34, 170.52, 171.53, 171.54, 194.26. ESI MS: 499.3 ([M + H]^+^). HR ESI MS: calcd for C_25_H_35_O_5_N_6_ 499.26634; found 499.26585.

### General Method for Synthesis of Compounds **8a**–**8l**

Fmoc-AA-OH (4.84 mmol, 1.1 equiv) and HATU (1.92 g, 5.06 mmol, 1.15 equiv) were suspended in anhydrous DCM (30 mL) under inert atmosphere, and the reaction mixture was cooled to 0 °C. DIPEA (1.71 g, 2.30 mL, 13.2 mmol, 3 equiv) was added, and the mixture was stirred for 5 min. Finally, a solution of compound **5e** (1.00 g, 4.40 mmol, 1 equiv) in anhydrous DCM (15 mL) was slowly added for 5 min. The resulting mixture was stirred for 30 min at 0 °C and then for 1–16.5 h at rt. DCM was evaporated, EtOAc (100 mL) was added, and the organic phase was washed with saturated NaHCO_3_ (50 mL), distilled H_2_O (50 mL), 10% KHSO_4_ (50 mL), distilled H_2_O (50 mL), and saturated NaCl (50 mL) and was dried over anhydrous MgSO_4_. EtOAc was evaporated, and the residue was purified by LC on silica gel (various mobile phases) to obtain desired products **8a**–**8l**.

#### *tert*-Butyl (*S*)-2-((*S*)-2-((((9*H*-Fluoren-9-yl)methoxy)carbonyl)amino)-3-(1-methyl-1*H*-indol-3-yl)propanamido)-6-diazo-5-oxohexanoate (**8a**)

Starting material Fmoc-l-Trp(N-Me)-OH (2.13 g), reaction time 3 h, mobile phase: DCM/EtOAc, 3:1. Product **8a** was isolated as a light-yellow solid (2.26 g) in 79% yield. ^1^H NMR (401 MHz, CDCl_3_): δ 1.43 (s, 9H), 1.81–1.93 (m, 1H), 2.05–2.25 (m, 3H), 3.16 (dd, *J* = 14.6, 7.1, 1H), 3.32–3.45 (m, 1H), 3.73 (s, 3H), 4.21 (t, *J* = 7.1, 1H), 4.33–4.40 (m, 2H), 4.44 (dd, *J* = 10.5, 7.3, 1H), 4.53 (d, *J* = 7.1, 1H), 5.04 (bs, 1H), 5.49 (d, *J* = 7.7, 1H), 6.55 (d, *J* = 7.6, 1H), 6.91 (bs, 1H), 7.13 (td, *J* = 7.4, 6.9, 1.2, 1H), 7.23 (ddd, *J* = 8.2, 6.8, 1.1, 1H), 7.27–7.33 (m, 3H), 7.40 (tdd, *J* = 7.6, 2.3, 1.3, 2H), 7.52–7.62 (m, 2H), 7.68 (d, *J* = 8.0, 1H), 7.74–7.79 (m, 2H). ^13^C NMR (101 MHz, CDCl_3_): δ 27.36, 28.05 (3C), 28.38, 32.80, 36.33, 47.26, 52.57, 54.68, 55.61, 67.20, 82.50, 108.63, 109.43, 119.13, 119.48, 120.08, 120.09, 122.00, 125.25, 125.30, 127.22 (2C), 127.84 (2C), 128.03, 128.34, 137.17, 141.39 (2C), 143.89, 143.98, 156.09, 170.48, 171.30, 193.79. ESI MS: 672.3 ([M + Na]^+^). HR ESI MS: calcd for C_37_H_39_O_6_N_5_Na 672.27926; found 672.27867.

#### *tert*-Butyl (*S*)-2-((*S*)-2-((((9*H*-Fluoren-9-yl)methoxy)carbonyl)amino)-3-phenylpropanamido)-6-diazo-5-oxohexanoate (**8b**)

Starting material Fmoc-l-Phe-OH (1.88 g); reaction time 16 h; mobile phase: DCM/EtOAc, 5:1. Product **8b** was isolated as a light-yellow solid (2.00 g) in 76% yield. ^1^H NMR (401 MHz, *d*_6_-DMSO): δ 1.40 (s, 9H), 1.76–1.87 (m, 1H), 1.93–2.06 (m, 1H), 2.35–2.43 (m, 2H), 2.79 (dd, *J* = 13.8, 10.9, 1H), 3.02 (dd, *J* = 13.8, 3.6, 1H), 4.05–4.21 (m, 4H), 4.29 (ddd, *J* = 10.9, 8.8, 3.6, 1H), 6.04 (bs, 1H), 7.15–7.22 (m, 1H), 7.22–7.44 (m, 8H), 7.63 (dd, *J* = 10.6, 7.5, 3H), 7.84–7.90 (m, 2H), 8.37 (d, *J* = 7.5, 1H). ^13^C NMR (101 MHz, *d*_6_-DMSO): δ 26.0, 27.6 (3C), 36.3, 37.4, 46.5, 52.1, 55.9, 56.6, 65.6, 80.7, 120.1 (2C), 125.3 (2C), 126.4, 127.0 (2C), 127.6 (2C), 128.0 (2C), 129.2 (2C), 138.1 (2C), 140.6, 143.7, 143.8, 155.8, 170.7, 171.8, 194.1. ESI MS: 619.3 ([M + Na]^+^). HR ESI MS: calcd for C_34_H_36_O_6_N_4_Na 619.25271; found 619.25162.

#### *tert*-Butyl (*S*)-2-((*R*)-2-((((9*H*-Fluoren-9-yl)methoxy)carbonyl)amino)-3-phenylpropanamido)-6-diazo-5-oxohexanoate (**8c**)

Starting material Fmoc-d-Phe-OH (1.88 g); reaction time 5 h; mobile phase: cyclohexane/EtOAc, 2:1. Product **8c** was isolated as a light-yellow solid (2.52 g) in 87% yield. ^1^H NMR (401 MHz, *d*_6_-DMSO): 1.42 (s, 9H), 1.78–1.90 (m, 1H), 2.05–2.22 (m, 3H), 3.02–3.17 (m, 2H), 4.18 (t, *J* = 7.0, 1H), 4.24–4.34 (m, 1H), 4.39 (dd, *J* = 7.2, 10.5, 2H), 4.44–4.56 (m, 1H), 5.17 (bs, 1H), 5.51 (d, *J* = 7.8, 1H), 6.76 (bs, 1H), 7.23 (t, *J* = 7.2, 3H), 7.29 (t, *J* = 7.2, 4H), 7.39 (t, *J* = 7.4, 2H), 7.52 (t, *J* = 6.8, 2H), 7.75 (d, *J* = 7.5, 2H). ^13^C NMR (101 MHz, *d*_6_-DMSO): δ 27.2, 28.0, 36.2, 38.7, 47.1, 52.4, 53.5, 54.7, 56.3, 67.2, 82.6, 120.0, 125.1, 125.2, 127.1, 127.1, 127.8, 128.8, 129.4, 136.5, 141.3, 141.3, 143.8, 143.8, 156.0, 170.6, 170.8, 193.7. ESI MS: 619.2 ([M + Na]^+^). HR ESI MS: calcd for C_34_H_36_O_6_N_4_Na 619.25271; found 619.25300.

#### *tert*-Butyl (*S*)-2-((*S*)-2-((((9*H*-Fluoren-9-yl)methoxy)carbonyl)amino)-2-phenylacetamido)-6-diazo-5-oxohexanoate (**8d**)

Starting material Fmoc-l-Phg-OH (1.81 g); reaction time 1.5 h; mobile phase: DCM/EtOAc, 10:1. Product **8d** was isolated as a yellow solid (1.74 g) in 68% yield. ^1^H NMR (401 MHz, *d*_6_-DMSO): δ 1.26 (s, 9H), 1.74–1.85 (m, 1H), 1.94 (dq, *J* = 14.2, 7.3, 1H), 2.30–2.42 (m, 2H), 4.08–4.16 (m, 1H), 4.23 (q, *J* = 5.7, 3H), 5.30 (d, *J* = 8.5, 1H), 6.02 (bs, 1H), 7.32 (ddd, *J* = 17.8, 8.0, 5.1, 5H), 7.38–7.49 (m, 4H), 7.77 (d, *J* = 7.5, 2H), 7.88 (d, *J* = 7.5, 2H), 8.07 (d, *J* = 8.5, 1H), 8.52 (d, *J* = 7.4, 1H). ^13^C NMR (101 MHz, CDCl_3_): δ 27.2, 27.9 (3C), 29.8, 36.5, 47.2, 53.1, 55.0, 59.0, 82.6, 120.0 (2C), 125.2, 125.2, 127.2 (2C), 127.4, 127.8 (2C), 128.7, 129.2 (2C), 129.3, 137.6, 141.4 (2C), 143.9, 144.0, 155.8, 169.8, 170.1, 194.0. ESI MS: 605.2 ([M + Na]^+^). HR ESI MS: calcd for C_33_H_34_O_6_N_4_Na 605.23706; found 605.23743.

#### *tert*-Butyl (*S*)-2-((*S*)-2-((((9*H*-Fluoren-9-yl)methoxy)carbonyl)amino)-4-phenylbutanamido)-6-diazo-5-oxohexanoate (**8e**)

Starting material Fmoc-l-HomoPhe-OH (1.94 g); reaction time 1.5 h; mobile phase: DCM/EtOAc, 10:1. Product **8e** was isolated as a yellow solid (2.31 g) in 86% yield. ^1^H NMR (401 MHz, CDCl_3_): δ 1.46 (s, 9H), 1.90–2.02 (m, 2H), 2.14–2.24 (m, 2H), 2.28–2.44 (m, 2H), 2.69 (d, *J* = 8.2, 2H), 4.21 (dt, *J* = 11.4, 6.8, 2H), 4.33–4.51 (m, 3H), 5.18 (bs, 1H), 5.37 (d, *J* = 8.1, 1H), 6.66 (d, *J* = 7.7, 1H), 7.16–7.23 (m, 3H), 7.27–7.35 (m, 4H), 7.40 (tdd, *J* = 7.5, 6.0, 2.6, 2H), 7.60 (d, *J* = 7.4, 2H), 7.73–7.79 (m, 2H). ^13^C NMR (101 MHz, CDCl_3_): δ 27.1, 28.1 (3C), 31.7, 34.5, 36.5, 47.2, 52.6, 54.7, 59.8, 67.2, 82.6, 120.1, 120.1, 125.2, 125.2, 126.3, 127.3 (2C), 127.9 (2C), 128.5 (2C), 128.6 (2C), 140.9, 141.4 (2C), 143.9, 143.9, 156.2, 170.6, 171.6, 194.0. ESI MS: 633.3 ([M + Na]^+^). HR ESI MS: calcd for C_35_H_38_O_6_N_4_Na 633.26836; found 633.26825.

#### *tert*-Butyl (*S*)-2-((*S*)-2-((((9*H*-Fluoren-9-yl)methoxy)carbonyl)amino)-3-(4-fluorophenyl)propanamido)-6-diazo-5-oxohexanoate (**8f**)

Starting material Fmoc-l-Phe(4-F)-OH (1.96 g); reaction time 1.5 h; mobile phase: DCM/EtOAc, 5:1. Product **8f** was isolated as a light-yellow solid (2.08 g) in 77% yield. ^1^H NMR (401 MHz, *d*_6_-DMSO): δ 1.39 (s, 9H), 1.81 (dtd, *J* = 14.7, 9.0, 6.1, 1H), 1.92–2.05 (m, 1H), 2.30–2.44 (m, 2H), 2.77 (dd, *J* = 13.8, 10.9, 1H), 3.00 (dd, *J* = 13.7, 3.7, 1H), 4.05–4.21 (m, 4H), 4.27 (ddt, *J* = 10.8, 8.7, 3.7, 1H), 6.04 (bs, 1H), 7.08 (dd, *J* = 10.1, 7.7, 2H), 7.23–7.45 (m, 6H), 7.62 (dd, *J* = 8.2, 4.3, 3H), 7.88 (d, *J* = 7.5, 2H), 8.36 (d, *J* = 7.5, 1H). ^13^C NMR (101 MHz, CDCl_3_): δ 26.0, 27.6 (3C), 33.7, 36.6, 46.5, 52.1, 55.9, 59.0, 65.6, 80.7, 114.6, 114.8, 120.0 (2C), 125.2, 125.3, 127.0 (2C), 127.6 (2C), 131.0, 131.0, 134.2, 140.6, 143.7, 143.8, 155.8, 159.8, 162.2, 170.7, 171.7, 194.1. ESI MS: 637.3 ([M + Na]^+^). HR ESI MS: calcd for C_34_H_35_O_6_N_4_FNa 637.24328; found 637.24402.

#### *tert*-Butyl (*S*)-2-((*S*)-2-((((9*H*-Fluoren-9-yl)methoxy)carbonyl)amino)-3-(3-fluorophenyl)propanamido)-6-diazo-5-oxohexanoate (**8g**)

Starting material Fmoc-l-Phe(3-F)-OH (1.96 g); reaction time 16 h; mobile phase: DCM/MeOH, 40:1. Product **8g** was isolated as a yellow solid (2.43 g) in 90% yield. ^1^H NMR (401 MHz, CDCl_3_): δ 1.44 (s, 9H), 1.72–2.01 (m, 2H), 2.09–2.42 (m, 2H), 3.01–3.15 (m, 2H), 4.19 (t, *J* = 6.8, 1H), 4.26–4.53 (m, 4H), 5.18 (s, 1H), 5.45 (bs, 1H), 6.67–7.04 (m, 4H), 7.19–7.28 (m, 1H), 7.30 (t, *J* = 7.4, 2H), 7.40 (dd, *J* = 8.3, 6.9, 2H), 7.50–7.60 (m, 2H), 7.70–7.83 (m, 2H). ^13^C NMR (101 MHz, CDCl_3_): δ 27.3, 28.0, 36.4, 38.3, 47.2, 52.6, 53.5, 54.9, 55.9, 67.2, 82.7, 114.0, 114.2, 116.4, 116.6, 120.1, 125.1, 125.2, 127.2, 127.9, 130.2, 130.2, 138.9, 141.4, 143.9, 155.9, 161.7, 164.2, 170.4, 170.5, 193.9. ESI MS: 637.4 ([M + Na]^+^). HR ESI MS: calcd for C_34_H_35_O_6_N_4_FNa 637.24328; found 637.24253.

#### *tert*-Butyl (*S*)-2-((*S*)-2-((((9*H*-Fluoren-9-yl)methoxy)carbonyl)amino)-3-(4-(trifluoromethyl)phenyl)propanamido)-6-diazo-5-oxohexanoate (**8h**)

Starting material Fmoc-l-Phe(4-CF_3_)-OH (2.20 g); reaction time 3.5 h; without purification to the following step (low solubility). Product **8h** was isolated as a light-yellow solid (2.92 g) in quantitative yield. ^1^H NMR (401 MHz, CDCl_3_): δ 1.40 (s, 9H), 1.77–1.89 (m, 1H), 1.93–2.06 (m, 1H), 2.36–2.45 (m, 2H), 2.84–2.94 (m, 2H), 4.10–4.22 (m, 4H), 4.30–4.39 (m, 1H), 6.03 (bs, 1H), 7.23–7.34 (m, 2H), 7.40 (dtd, *J* = 8.6, 4.6, 2.4, 3H), 7.56 (t, *J* = 8.7, 2H), 7.59–7.65 (m, 3H), 7.68 (d, *J* = 8.8, 1H), 7.85–7.91 (m, 2H), 8.40 (d, *J* = 7.5, 1H). ESI MS: 687.3 ([M + Na]^+^). HR ESI MS: calcd for C_35_H_35_O_6_N_4_F_3_Na 687.24009; found 687.23944.

#### *tert*-Butyl (*S*)-2-(2-((((9*H*-Fluoren-9-yl)methoxy)carbonyl)amino)acetamido)-6-diazo-5-oxohexanoate (**8i**)

Starting material Fmoc-Gly-OH (1.44 g); reaction time 2 h; mobile phase: EtOAc. Product **8i** was isolated as a light-yellow solid (2.05 g) in 92% yield. ^1^H NMR (401 MHz, CDCl_3_): δ 1.44 (s, 9H), 1.97 (dt, *J* = 14.5, 7.5, 1H), 2.14–2.25 (m, 1H), 2.25–2.45 (m, 2H), 3.91 (d, *J* = 5.7, 2H), 4.21 (t, *J* = 7.2, 1H), 4.38 (d, *J* = 7.0, 2H), 4.48 (td, *J* = 8.1, 4.6, 1H), 5.27 (bs, 1H), 5.84 (t, *J* = 5.7, 1H), 7.06 (d, *J* = 7.8, 1H), 7.28 (t, *J* = 7.6, 2H), 7.37 (t, *J* = 7.4, 2H), 7.58 (d, *J* = 7.5, 2H), 7.73 (d, *J* = 7.6, 2H). ^13^C NMR (101 MHz, CDCl_3_): δ 27.32, 27.97 (3C), 36.44, 44.43, 47.09, 52.41, 54.87, 59.71, 67.30, 82.57, 120.00 (2C), 125.13, 125.15, 127.12 (2C), 127.76 (2C), 141.28, 141.28, 143.81, 143.83, 156.68, 169.16, 170.78, 193.89. ESI MS: 529.2 ([M + Na]^+^). HR ESI MS: calcd for C_27_H_30_O_6_N_4_Na 529.20576; found 529.20604.

#### *tert*-Butyl (*S*)-2-((*S*)-2-((((9*H*-Fluoren-9-yl)methoxy)carbonyl)amino)propanamido)-6-diazo-5-oxohexanoate (**8j**)

Starting material Fmoc-l-Ala-OH monohydrate (1.59 g); reaction time 4 h; mobile phase: DCM/EtOAc, 3:1. Product **8j** was isolated as a light-yellow solid (2.24 g) in 98% yield. ^1^H NMR (401 MHz, CDCl_3_): δ 1.41 (d, *J* = 7.4, 3H), 1.44 (s, 9H), 1.97 (tt, *J* = 14.6, 7.2, 1H), 2.18 (ddd, *J* = 14.8, 7.1, 2.5, 1H), 2.25–2.48 (m, 2H), 4.21 (t, *J* = 7.1, 1H), 4.28 (t, *J* = 7.2, 1H), 4.37 (dd, *J* = 7.4, 3.1, 2H), 4.43 (td, *J* = 8.2, 4.6, 1H), 5.21 (bs, 1H), 5.59 (d, *J* = 7.5, 1H), 6.91 (d, *J* = 7.8, 1H), 7.30 (td, *J* = 7.5, 1.0, 2H), 7.39 (dd, *J* = 8.2, 6.7, 2H), 7.59 (d, *J* = 7.5, 2H), 7.75 (d, *J* = 7.5, 2H). ^13^C NMR (101 MHz, CDCl_3_): δ 18.93, 27.30, 28.04 (3C), 36.52, 47.18, 50.61, 52.56, 54.96, 67.19, 82.56, 120.07, 120.08, 125.19, 125.22, 127.19 (2C), 127.83 (2C), 141.36 (2C), 143.90 (2C), 155.99, 170.66, 172.37, 194.04. ESI MS: 543.2 ([M + Na]^+^). HR ESI MS: calcd for C_28_H_32_O_6_N_4_Na 543.22141; found 543.22096.

#### *tert*-Butyl (*S*)-2-((*S*)-2-((((9*H*-Fluoren-9-yl)methoxy)carbonyl)amino)-4-methylpentanamido)-6-diazo-5-oxohexanoate (**8k**)

Starting material Fmoc-l-Leu-OH (1.71 g); reaction time 2 h; mobile phase: cyclohexane/EtOAc, 1:1. Product **8k** was isolated as a light-yellow solid (1.88 g) in 76% yield. ^1^H NMR (401 MHz, CDCl_3_): δ 0.82–1.00 (m, 7H), 1.45 (s, 9H), 1.51–1.74 (m, 2H), 1.96 (dq, *J* = 14.8, 7.7, 1H), 2.12–2.26 (m, 1H), 2.24–2.45 (m, 2H), 4.14–4.26 (m, 2H), 4.31–4.47 (m, 3H), 5.18 (bs, 1H), 5.27 (d, *J* = 8.3, 1H), 6.68 (d, *J* = 7.8, 1H), 7.31 (tt, *J* = 7.4, 1.0, 2H), 7.40 (tt, *J* = 7.5, 1.5, 2H), 7.59 (d, *J* = 7.5, 2H), 7.71–7.81 (m, 2H). ^13^C NMR (101 MHz, CDCl_3_): δ 22.07, 23.11, 24.80, 27.46, 28.09 (3C), 36.51, 41.83, 47.27, 52.55, 53.71, 54.92, 67.20, 82.63, 120.11, 120.14, 125.18, 125.25, 127.24 (2C), 127.87, 127.88, 141.42 (2C), 143.90, 143.93, 156.27, 170.66, 172.24, 193.98. ESI MS: 585.2 ([M + Na]^+^). HR ESI MS: calcd for C_31_H_38_O_6_N_4_Na 585.26836; found 585.26795.

#### *tert*-Butyl (*S*)-2-((*S*)-2-((((9*H*-Fluoren-9-yl)methoxy)carbonyl)amino)-4,4-dimethylpentanamido)-6-diazo-5-oxohexanoate (**8l**)

Starting material Fmoc-l-Ala(*t*Bu)-OH (1.78 g); reaction time 16 h; mobile phase: DCM/MeOH, 50:1. Product **8l** was isolated as a yellow solid (2.28 g) in 90% yield. ^1^H NMR (401 MHz, CDCl_3_): δ 0.97 (s, 9H), 1.44 (s, 9H), 1.78–2.01 (m, 2H), 2.09–2.44 (m, 2H), 4.15–4.36 (m, 2H), 4.36–4.50 (m, 2H), 5.16 (bs, 1H), 5.34 (d, *J* = 8.5, 1H), 6.78 (d, *J* = 7.9, 1H), 7.29 (td, *J* = 7.5, 1.2, 2H), 7.34–7.42 (m, 2H), 7.54–7.62 (m, 2H), 7.71–7.77 (m, 2H). ^13^C NMR (101 MHz, CDCl_3_): δ 28.0, 29.8, 30.6, 36.5, 46.0, 47.2, 52.5, 53.0, 53.5, 54.8, 67.2, 82.5, 120.1, 120.1, 125.1, 125.2, 127.2, 127.2, 127.8, 141.3, 143.8, 143.9, 156.0, 170.6, 172.7, 193.9. ESI MS: 599.5 ([M + Na]^+^). HR ESI MS: calcd for C_32_H_41_O_6_N_4_ 577.30206; found 577.30234.

### General Method for Synthesis of Compounds **9a**–**9l**

Compounds **8a**–**8l** (3.00 mmol, 1 equiv) were dissolved in anhydrous DCM (27 mL), and diethylamine (2.19 g, 3.10 mL, 30.0 mmol, 10 equiv) was added. The reaction mixture was stirred at rt for 1.5–7 h under an inert atmosphere. Volatiles were evaporated, and the residue was purified by LC on silica gel (various mobile phases) to afford products **9a**–**9l**.

#### *tert*-Butyl (*S*)-2-((*S*)-2-Amino-3-(1-methyl-1*H*-indol-3-yl)propanamido)-6-diazo-5-oxohexanoate (**9a**)

Starting material **8a** (1.95 g); reaction time 7 h; mobile phase: DCM/MeOH, 30:1. Product **9a** was isolated as a yellow solid (1.15 g) in 90% yield. ^1^H NMR (401 MHz, CDCl_3_): δ 1.46 (s, 9H), 1.55 (bs, 2H), 1.86–2.00 (m, 1H), 2.07–2.32 (m, 3H), 2.98 (dd, *J* = 14.4, 8.5, 1H), 3.31 (dd, *J* = 14.0, 3.8, 1H), 3.72 (dd, *J* = 8.5, 4.1, 1H), 3.76 (s, 3H), 4.41–4.51 (m, 1H), 5.12 (bs, 1H), 6.94 (s, 1H), 7.12 (ddd, *J* = 8.0, 6.9, 1.1, 1H), 7.23 (ddd, *J* = 8.2, 6.9, 1.1, 1H), 7.29 (dt, *J* = 8.2, 1.0, 1H), 7.68 (dt, *J* = 8.0, 1.0, 1H), 7.87 (d, *J* = 8.4, 1H). ^13^C NMR (101 MHz, CDCl_3_): δ 27.95, 28.11 (3C), 30.76, 32.81, 36.74, 51.96, 54.66, 55.63, 82.40, 109.38, 110.07, 119.26, 119.41, 121.97, 128.08, 137.27 (2C), 171.15, 174.92, 193.85. ESI MS: 450.2 ([M + Na]^+^). HR ESI MS: calcd for C_22_H_29_O_4_N_5_Na 450.21118; found 450.21112.

#### *tert*-Butyl (*S*)-2-((*S*)-2-Amino-3-phenylpropanamido)-6-diazo-5-oxohexanoate (**9b**)

Starting material **8b** (1.79 g); reaction time 2 h; mobile phase: DCM/MeOH, 30:1. Product **9b** was isolated as a yellow solid (1.08 g) in 96% yield. ^1^H NMR (401 MHz, CDCl_3_): δ 1.40 (s, 9H), 1.71 (bs, 2H), 1.75–1.85 (m, 1H), 1.90–1.99 (m, 1H), 2.24–2.38 (m, 2H), 2.59 (dd, *J* = 13.4, 8.4, 1H), 2.95 (dd, *J* = 13.4, 4.5, 1H), 3.43 (dd, *J* = 8.4, 4.5, 1H), 4.07–4.17 (m, 1H), 6.05 (bs, 1H), 7.17–7.29 (m, 5H), 8.13 (d, *J* = 7.9, 1H). ^13^C NMR (101 MHz, CDCl_3_): δ 27.8, 28.1 (3C), 36.8, 41.1, 50.8, 54.8, 56.5, 82.5, 127.0, 128.8 (2C), 129.5 (2C), 137.8, 171.0, 174.4, 193.7. ESI MS: 397.2 ([M + Na]^+^). HR ESI MS: calcd for C_19_H_26_O_4_N_4_Na 397.18463; found 397.18427.

#### *tert*-Butyl (*S*)-2-((*R*)-2-Amino-3-phenylpropanamido)-6-diazo-5-oxohexanoate (**9c**)

Starting material **8c** (1.79 g); reaction time 2.5 h; mobile phase: DCM/MeOH, 20:1. Product **9c** was isolated as a yellow solid (1.10 g) in 98% yield. ^1^H NMR (401 MHz, CDCl_3_): δ 1.44 (s, 9H), 1.88–2.00 (m, 1H), 2.10–2.21 (m, 1H), 2.22–2.41 (m, 2H), 2.64 (dd, *J* = 9.6, 13.8, 1H), 3.24 (dd, *J* = 4.3, 13.8, 1H), 3.57 (dd, *J* = 4.3, 9.5, 1H), 4.40–4.48 (m, 1H), 5.28 (bs, 1H), 7.16–7.24 (m, 3H), 7.24–7.31 (m, 2H), 7.74 (d, *J* = 8.4, 1H). ^13^C NMR (101 MHz, CDCl_3_): δ 27.8, 28.0, 36.8, 41.0, 52.0, 54.7, 56.7, 82.4, 126.8, 128.7, 129.3, 138.0, 170.9, 174.4, 193.7. ESI MS: 375.2 ([M + H]^+^). HR ESI MS: calcd for C_19_H_27_O_4_N_4_ 375.20268; found 375.20286.

#### *tert*-Butyl (*S*)-2-((*S*)-2-Amino-2-phenylacetamido)-6-diazo-5-oxohexanoate (**9d**)

Starting material **8d** (1.75 g); reaction time 3 h; mobile phase: DCM/MeOH, 30:1. Product **9d** was isolated as a yellow solid (822 mg) in 76% yield. ^1^H NMR (401 MHz, CDCl_3_): δ 1.45 (s, 9H), 1.64 (bs, 2H), 1.93 (ddt, *J* = 11.7, 8.3, 3.6, 1H), 2.10–2.35 (m, 3H), 4.44 (td, *J* = 8.5, 4.0, 1H), 4.55 (s, 1H), 5.04 (bs, 1H), 7.27–7.45 (m, 5H), 7.80 (d, *J* = 8.3, 1H). ^13^C NMR (101 MHz, CDCl_3_): δ 27.9, 28.0 (3C), 36.5, 51.9, 54.6, 60.0, 82.4, 126.7 (2C), 128.0, 128.8 (2C), 140.9, 170.9, 173.2, 193.7. ESI MS: 361.2 ([M + H]^+^). HR ESI MS: calcd for C_18_H_25_O_4_N_4_ 361.18703; found 361.18675.

#### *tert*-Butyl (*S*)-2-((*S*)-2-Amino-4-phenylbutanamido)-6-diazo-5-oxohexanoate (**9e**)

Starting material **8e** (1.83 g); reaction time 3 h; mobile phase: DCM/MeOH, 30:1. Product **9e** was isolated as a yellow solid (886 mg) in 76% yield. ^1^H NMR (401 MHz, CDCl_3_): δ 1.47 (s, 9H), 1.62 (bs, 2H), 1.79 (dtd, *J* = 14.3, 8.9, 6.0, 1H), 1.98 (dtd, *J* = 14.5, 8.5, 6.1, 1H), 2.15–2.25 (m, 2H), 2.36 (t, *J* = 21.3, 2H), 2.66–2.81 (m, 2H), 3.38 (dd, *J* = 8.4, 4.4, 1H), 4.46 (td, *J* = 8.4, 4.7, 1H), 5.28 (bs, 1H), 7.16–7.24 (m, 3H), 7.26–7.32 (m, 2H), 7.80 (d, *J* = 8.4, 1H). ^13^C NMR (101 MHz, CDCl_3_): δ 28.0, 28.1 (3C), 32.4, 37.0, 52.0, 53.5, 54.7, 55.0, 82.4, 126.2, 128.5 (2C), 128.6 (2C), 141.2, 171.1, 175.1, 193.7. ESI MS: 389.2 ([M + H]^+^). HR ESI MS: calcd for C_20_H_29_O_4_N_4_ 389.21833; found 389.21798.

#### *tert*-Butyl (*S*)-2-((*S*)-2-Amino-3-(4-fluorophenyl)propanamido)-6-diazo-5-oxohexanoate (**9f**)

Starting material **8f** (1.22 g); reaction time 3 h; mobile phase: DCM/MeOH, 30:1. Product **9f** was isolated as a yellow solid (977 mg) in 83% yield. ^1^H NMR (401 MHz, CDCl_3_): δ 1.41 (bs, 2H), 1.46 (s, 9H), 1.97 (dtd, *J* = 14.3, 8.4, 5.6, 1H), 2.17 (td, *J* = 13.5, 5.7, 1H), 2.30 (d, *J* = 28.2, 2H), 2.75 (dd, *J* = 13.8, 8.9, 1H), 3.17 (dd, *J* = 13.8, 4.0, 1H), 3.61 (dd, *J* = 8.9, 4.1, 1H), 4.45 (td, *J* = 8.2, 4.6, 1H), 5.26 (bs, 1H), 6.96–7.05 (m, 2H), 7.15–7.22 (m, 2H), 7.80 (d, *J* = 8.2, 1H). ^13^C NMR (101 MHz, CDCl_3_): δ 27.8, 28.1 (3C), 36.7, 40.2, 52.0, 54.8, 56.5, 82.5, 115.5, 115.7, 130.9, 131.0, 133.4, 160.8, 171.0, 174.2, 193.7. ESI MS: 393.2 ([M + H]^+^). HR ESI MS: calcd for C_19_H_26_O_4_N_4_F 393.19326; found 393.19330.

#### *tert*-Butyl (*S*)-2-((*S*)-2-Amino-3-(3-fluorophenyl)propanamido)-6-diazo-5-oxohexanoate (**9g**)

Starting material **8g** (1.22 g); reaction time 2 h; mobile phase: DCM/MeOH, 20:1. Product **9g** was isolated as a yellow solid (1.04 mg) in 88% yield. ^1^H NMR (401 MHz, CDCl_3_): δ 1.42 (d, *J* = 1.3, 9H), 1.82–2.00 (m, 1H), 2.06–2.44 (m, 3H), 2.76 (dd, *J* = 13.7, 8.7, 1H), 3.15 (dd, *J* = 13.7, 4.0, 1H), 3.59 (ddd, *J* = 8.7, 4.1, 1.1, 1H), 4.41 (dtd, *J* = 8.3, 4.6, 2.3, 1H), 5.27 (bs, 1H), 6.84–7.04 (m, 3H), 7.18–7.32 (m, 1H), 7.82 (d, *J* = 8.3, 1H). ^13^C NMR (101 MHz, CDCl_3_): δ 28.0, 36.7, 40.7, 40.7, 52.0, 54.7, 56.2, 82.4, 113.7, 113.9, 116.1, 116.3, 125.1, 125.2, 130.1, 130.2, 140.2, 140.3, 161.7, 164.2, 170.9, 174.0, 193.7. ESI MS: 393.2 ([M + H]^+^). HR ESI MS: calcd for C_19_H_26_O_4_N_4_F 393.19326; found 393.19334.

#### *tert*-Butyl (*S*)-2-((*S*)-2-Amino-3-(4-(trifluoromethyl)phenyl)propanamido)-6-diazo-5-oxohexanoate (**9h**)

Starting material **8h** (1.99 g); reaction time 1.5 h; mobile phase: DCM/MeOH, 30:1. Product **9h** was isolated as a yellow solid (1.01 g) in 76% yield (over two steps). ^1^H NMR (401 MHz, CDCl_3_): δ 1.41 (bs, 2H), 1.44 (s, 9H), 1.88–2.05 (m, 1H), 2.11–2.42 (m, 3H), 2.82 (dd, *J* = 13.7, 8.9, 1H), 3.25 (dd, *J* = 13.7, 4.1, 1H), 3.64 (dd, *J* = 8.9, 4.1, 1H), 4.43 (td, *J* = 8.1, 4.4, 1H), 5.24 (bs, 1H), 7.34 (d, *J* = 7.9, 2H), 7.56 (d, *J* = 7.9, 2H), 7.82 (d, *J* = 8.2, 1H). ^13^C NMR (101 MHz, CDCl_3_): δ 27.55, 27.97 (3C), 36.57, 40.77, 52.03, 54.69, 56.20, 82.45, 125.58 (q, *J* = 3.7 Hz, 2C), 129.20 (q, *J* = 32.4 Hz), 129.74 (2C), 141.95 (2C), 170.84, 173.73, 193.50. ESI MS: 443.2 ([M + H]^+^). HR ESI MS: calcd for C_20_H_26_O_4_N_4_F_3_ 443.19007; found 443.19016.

#### *tert*-Butyl (*S*)-2-(2-Aminoacetamido)-6-diazo-5-oxohexanoate (**9i**)

Starting material **8i** (1.52 g); reaction time 3 h; mobile phase: DCM/MeOH, 10:1. Product **9i** was isolated as a yellow-orange amorphous compound (768 mg) in 90% yield. ^1^H NMR (401 MHz, CDCl_3_): δ 1.43 (s, 9H), 1.73 (bs, 2H), 1.95 (dtd, *J* = 14.5, 8.6, 6.1, 1H), 2.17 (dddd, *J* = 13.4, 8.5, 6.7, 4.7, 1H), 2.26–2.48 (m, 2H), 3.34 (s, 2H), 4.47 (td, *J* = 8.4, 4.7, 1H), 5.31 (bs, 1H), 7.75 (d, *J* = 8.4, 1H). ^13^C NMR (101 MHz, CDCl_3_): δ 27.97, 28.04 (3C), 36.82, 44.75, 51.87, 54.82, 82.48, 171.04, 172.92, 193.85. ESI MS: 307.1 ([M + Na]^+^). HR ESI MS: calcd for C_12_H_20_O_4_N_4_Na 307.13768; found 307.13744.

#### *tert*-Butyl (*S*)-2-(2-Aminopropanamido)-6-diazo-5-oxohexanoate (**9j**)

Starting material **8j** (1.56 g); reaction time 3 h; mobile phase: DCM/MeOH, 10:1 to 5:1. Product **9j** was isolated as a yellow amorphous compound (841 mg) in 94% yield. ^1^H NMR (401 MHz, CDCl_3_): δ 1.36 (d, *J* = 7.0, 3H), 1.46 (s, 9H), 1.94–2.02 (m, 1H), 2.14–2.17 (m, 2H), 2.18–2.23 (m, 1H), 2.30–2.47 (m, 2H), 3.56 (q, *J* = 7.0, 1H), 4.40–4.47 (m, 1H), 5.34 (bs, 1H), 7.73–7.77 (m, 1H). ^13^C NMR (101 MHz, CDCl_3_): δ 21.08, 27.23, 27.68 (3C), 36.30, 50.27, 51.77, 54.70, 77.16, 81.98, 170.63, 175.25, 193.96. ESI MS: 299.2 ([M + H]^+^). HR ESI MS: calcd for C_13_H_22_O_4_N_4_Na 321.15387; found 321.15392.

#### *tert*-Butyl (*S*)-2-((*S*)-2-Amino-4-methylpentanamido)-6-diazo-5-oxohexanoate (**9k**)

Starting material **8k** (1.69 g); reaction time 2 h; mobile phase: DCM/MeOH, 15:1. Product **9k** was isolated as a yellow solid (950 mg) in 93% yield. ^1^H NMR (401 MHz, CDCl_3_): δ 0.91 (d, *J* = 6.3, 3H), 0.95 (d, *J* = 6.3, 3H), 1.27–1.36 (m, 1H), 1.44 (s, 9H), 1.47 (bs, 2H), 1.58–1.80 (m, 2H), 1.95 (dtd, *J* = 14.5, 8.6, 6.1, 1H), 2.10–2.24 (m, 1H), 2.25–2.47 (m, 2H), 3.37 (dd, *J* = 10.0, 3.8, 1H), 4.43 (td, *J* = 8.5, 4.7, 1H), 5.30 (bs, 1H), 7.82 (d, *J* = 8.4, 1H). ^13^C NMR (101 MHz, CDCl_3_): δ 21.36, 23.58, 24.99, 27.90, 28.09 (3C), 36.91, 44.31, 51.91, 53.64, 54.81, 82.39, 171.17, 175.92, 193.84. ESI MS: 341.2 ([M + H]^+^). HR ESI MS: calcd for C_16_H_29_O_4_N_4_ 341.21833; found 341.21816.

#### *tert*-Butyl (*S*)-2-((*S*)-2-Amino-4,4-dimethylpentanamido)-6-diazo-5-oxohexanoate (**9l**)

Starting material **8l** (1.73 g); reaction time 3 h; mobile phase: DCM/MeOH, 15:1. Product **9f** was isolated as a yellow amorphous compound (1.01 g) in 95% yield. ^1^H NMR (401 MHz, CDCl_3_): δ 0.96 (s, 9H), 1.18 (dd, *J* = 14.3, 8.7, 1H), 1.43 (s, 9H), 1.87 (dd, *J* = 14.3, 2.5, 1H), 1.90–2.01 (m, 1H), 2.09–2.22 (m, 1H), 2.24–2.47 (m, 2H), 3.37 (dd, *J* = 8.6, 2.5, 1H), 4.40 (td, *J* = 8.5, 4.7, 1H), 5.30 (bs, 1H), 7.88 (d, *J* = 8.4, 1H). ^13^C NMR (101 MHz, CDCl_3_): δ 28.1, 30.1, 30.8, 36.9, 49.6, 52.0, 53.1, 54.8, 82.3, 171.1, 176.4, 193.8. ESI MS: 355.3 ([M + H]^+^). HR ESI MS: calcd for C_17_H_31_O_4_N_4_ 355.23398; found 355.23361.

### General Method A for Synthesis of Prodrugs **P10**, **P11**, **P18**, and **P19**

Dimethylglycine (113 mg, 1.10 mmol, 1.1 equiv) and HATU (456 mg, 1.20 mmol, 1.2 equiv) were dissolved in anhydrous DMF or DCM (15 mL) under inert atmosphere, the mixture was cooled to 0 °C, and DIPEA (388 mg, 523 μL, 3.00 mmol, 3 equiv) was added. After 5 min of stirring, a solution of amines **9a**, **9b**, **9i**, and **9j** (1.00 mmol, 1 equiv) in anhydrous DMF or DCM (10 mL) was added. The resulting mixture was stirred for 30 min at 0 °C and 1–2 h at rt. The solvent was evaporated, EtOAc (100 mL) was added, and the organic phase was washed with saturated NaHCO_3_ (70 mL), distilled H_2_O (70 mL), and saturated NaCl (50 mL) and was dried over anhydrous MgSO_4_, and solvent was evaporated. The crude product was purified by LC on silica gel (various mobile phases) to afford desired prodrugs **P10**, **P11**, **P18**, and **P19**.

### General Method B for Synthesis of Prodrugs **P12**–**17**, **P20**, and **P21**

Compounds **9c**–**9h**, **9k**, **9l** (1.00 mmol, 1 equiv) and 2,5-dioxopyrrolidin-1-yl dimethylglycinate (Dmg-ONSu)^36^ (220 mg, 1.10 mmol, 1.1 equiv) were dissolved in anhydrous DCM (5 mL) under inert atmosphere. The resulting mixture was stirred at rt for 2–20 h. DCM (50 mL) was added, and the organic phase was washed with saturated NaHCO_3_ (30 mL), distilled H_2_O (30 mL), and saturated NaCl (20 mL) and was dried over anhydrous MgSO_4_, and the solvent was evaporated. The crude product was purified by LC on silica gel (various mobile phases) to obtain prodrugs **P12**–**P17**, **P20**, and **P21**.

#### *tert*-Butyl (*S*)-6-Diazo-2-((*S*)-2-(2-(dimethylamino)acetamido)-3-(1-methyl-1*H*-indol-3-yl)propanamido)-5-oxohexanoate (**P10**)

General method A, starting material **9a** (428 mg); solvent: DMF; reaction time 2 h; mobile phase: DCM/MeOH, 12:1. Prodrug **P10** was isolated as a light-yellow solid (375 mg) in 73% yield. ^1^H NMR (401 MHz, CDCl_3_): δ 1.39 (s, 9H), 1.85 (dtd, *J* = 14.3, 8.4, 5.8, 1H), 2.01–2.30 (m, 3H), 2.09 (s, 6H), 2.80 (d, *J* = 16.2, 1H), 2.92 (d, *J* = 16.2, 1H), 3.21 (d, *J* = 6.8, 2H), 3.69 (s, 3H), 4.30 (td, *J* = 7.9, 4.7, 1H), 4.61–4.67 (m, 1H), 5.22 (bs, 1H), 6.92 (s, 1H), 7.00 (d, *J* = 7.6, 1H), 7.06 (ddd, *J* = 8.0, 6.9, 1.1, 1H), 7.16 (ddd, *J* = 8.2, 6.9, 1.2, 1H), 7.22 (dt, *J* = 8.3, 1.0, 1H), 7.60 (dt, *J* = 8.0, 1.0, 1H), 7.70 (d, *J* = 7.9, 1H). ^13^C NMR (101 MHz, CDCl_3_): δ 27.18, 27.66, 27.91 (3C), 32.61, 36.26, 45.71 (2C), 52.36, 53.51, 54.83, 62.78, 82.29, 108.83, 109.21, 118.87, 119.06, 121.72, 127.95, 136.97 (2C), 170.46, 171.26, 171.42, 194.28. ESI MS: 535.3 ([M + Na]^+^). HR ESI MS: calcd for C_26_H_36_O_5_N_6_Na 535.26394; found 535.26373.

#### *tert*-Butyl (*S*)-6-Diazo-2-((*S*)-2-(2-(dimethylamino)acetamido)-3-phenylpropanamido)-5-oxohexanoate (**P11**)

General method A, starting material **9b** (374 mg); solvent: DCM; reaction time 2.5 h; mobile phase: DCM/MeOH, 15:1. Prodrug **P11** was isolated as a yellow amorphous compound (307 mg) in 67% yield. ^1^H NMR (401 MHz, CDCl_3_): δ 1.40 (s, 9H), 1.82–1.97 (m, 1H), 2.06–2.17 (m, 1H), 2.10 (s, 6H), 2.17–2.39 (m, 2H), 2.76 (d, *J* = 16.3, 1H), 2.93 (d, *J* = 16.3, 1H), 2.97 (dd, *J* = 14.0, 8.5, 1H), 3.15 (dd, *J* = 14.0, 5.9, 1H), 4.32 (td, *J* = 7.9, 4.7, 1H), 4.66 (td, *J* = 8.3, 5.9, 1H), 5.30 (bs, 1H), 7.02 (d, *J* = 8.6, 1H), 7.13–7.19 (m, 3H), 7.19–7.25 (m, 2H), 7.55 (d, *J* = 8.1, 1H). ^13^C NMR (101 MHz, CDCl_3_): δ 27.22, 27.95 (3C), 36.34, 37.82, 45.83 (2C), 52.45, 53.93, 54.72, 62.86, 82.29, 126.92, 128.57 (2C), 129.21 (2C), 136.58, 170.39, 170.98, 171.10, 193.84. ESI MS: 460.3 ([M + H]^+^). HR ESI MS: calcd for C_23_H_34_O_5_N_5_ 460.25545; found 460.25482.

#### *tert*-Butyl (*S*)-6-Diazo-2-((*R*)-2-(2-(dimethylamino)acetamido)-3-phenylpropanamido)-5-oxohexanoate (**P12**)

General method B, starting material **9c** (374 mg); solvent: DCM; reaction time 16 h; mobile phase: DCM/MeOH, 20:1. Prodrug **P12** was isolated as yellow solid (348 mg) in 76% yield. ^1^H NMR (401 MHz, CDCl_3_): δ 1.44 (s, 9H), 1.81–1.94 (m, 1H), 2.04–2.33 (m, 9H), 2.78 (d, *J* = 16.3, 1H), 2.95–3.09 (m, 2H), 3.24 (dd, *J* = 6.6, 14.0, 1H), 4.38 (td, *J* = 4.5, 8.0, 1H), 4.62–4.71 (td, *J* = 6.6, 8.3, 1H), 5.24 (bs, 1H), 6.79 (d, *J* = 7.6, 1H), 7.16–7.34 (m, 6H), 7.57 (d, *J* = 7.9, 1H). ^13^C NMR (101 MHz, CDCl_3_): δ 27.1, 28.1, 36.4, 37.8, 46.0, 52.5, 54.2, 54.8, 63.0, 82.5, 127.1, 128.8, 129.3, 136.9, 170.6, 170.9, 171.5, 193.9. ESI MS: 460.3 ([M + H]^+^). HR ESI MS: calcd for C_23_H_34_O_5_N_5_ 460.25545; found 460.25491.

#### *tert*-Butyl (*S*)-6-Diazo-2-((*S*)-2-(2-(dimethylamino)acetamido)-2-phenylacetamido)-5-oxohexanoate (**P13**)

General method B, starting material **9d** (360 mg); reaction time 20 h; mobile phase: DCM/MeOH, 15:1. Prodrug **P13** was isolated as a yellow amorphous compound (303 mg) in 68% yield. ^1^H NMR (401 MHz, CDCl_3_): δ 1.36 (s, 9H), 1.97 (dtd, *J* = 14.4, 8.0, 6.4, 1H), 2.11–2.25 (m, 1H), 2.30 (s, 6H), 2.32–2.51 (m, 2H), 2.92–3.04 (m, 2H), 4.39 (td, *J* = 7.9, 4.7, 1H), 5.31 (bs, 1H), 5.45 (d, *J* = 7.5, 1H), 6.52 (d, *J* = 7.5, 1H), 7.30–7.42 (m, 5H), 8.09 (d, *J* = 7.6, 1H). ^13^C NMR (101 MHz, CDCl_3_): δ 26.1, 27.1 (3C), 28.9, 45.3 (2C), 52.0, 55.2, 62.4, 69.8, 80.9, 126.7 (2C), 127.3, 128.0 (2C), 137.6, 169.3 (2C), 169.6, 193.4. ESI MS: 446.3 ([M + H]^+^). HR ESI MS: calcd for C_22_H_32_O_5_N_5_ 446.23980; found 446.23917.

#### *tert*-Butyl (*S*)-6-Diazo-2-((*S*)-2-(2-(dimethylamino)acetamido)-4-phenylbutanamido)-5-oxohexanoate (**P14**)

General method B, starting material **9e** (388 mg); reaction time 2 h; mobile phase: DCM/MeOH, 30:1 to 15:1. Prodrug **P14** was isolated as a yellow amorphous compound (242 mg) in 51% yield. ^1^H NMR (401 MHz, CDCl_3_): δ 1.46 (s, 9H), 1.67–1.85 (m, 2H), 1.91–2.05 (m, 2H), 2.12–2.27 (m, 2H), 2.31 (s, 6H), 2.70 (t, *J* = 7.8, 2H), 2.98 (d, *J* = 1.2, 2H), 4.37–4.45 (m, 2H), 5.32 (bs, 1H), 6.76 (d, *J* = 7.7, 1H), 7.16–7.22 (m, 3H), 7.27–7.31 (m, 2H), 7.62 (d, *J* = 8.3, 1H). ^13^C NMR (101 MHz, CDCl_3_): δ 27.3, 28.1 (3C), 32.0, 34.0, 46.0 (2C), 52.6, 52.8, 55.1, 63.0, 70.7, 82.5, 126.3, 128.5 (2C), 128.6 (2C), 140.9, 170.6, 171.0, 171.4, 194.0. ESI MS: 474.4 ([M + H]^+^). HR ESI MS: calcd for C_24_H_36_O_5_N_5_ 474.27110; found 474.27011.

#### *tert*-Butyl (*S*)-6-Diazo-2-((*S*)-2-(2-(dimethylamino)acetamido)-3-(4-fluorophenyl)propanamido)-5-oxohexanoate (**P15**)

General method B, starting material **9f** (392 mg); reaction time 16 h; mobile phase: DCM/MeOH, 20:1. Prodrug **P15** was isolated as a yellow solid (349 mg) in 73% yield. ^1^H NMR (401 MHz, CDCl_3_): δ 1.45 (s, 9H), 1.94 (dtd, *J* = 14.2, 8.0, 6.3, 1H), 2.09–2.16 (m, 1H), 2.18 (s, 6H), 2.23–2.43 (m, 2H), 2.80–2.90 (m, 1H), 2.94–3.06 (m, 2H), 3.14 (dd, *J* = 14.1, 6.6, 1H), 4.35 (td, *J* = 7.6, 4.8, 1H), 4.60 (td, *J* = 8.0, 6.6, 1H), 5.29 (bs, 1H), 6.73 (d, *J* = 7.4, 1H), 6.92–7.01 (m, 2H), 7.13–7.23 (m, 2H), 7.57 (d, *J* = 8.1, 1H). ^13^C NMR (101 MHz, CDCl_3_): δ 27.3, 28.1 (3C), 37.2, 46.0 (2C), 52.6, 54.2, 54.9, 63.0, 70.1, 82.6, 115.4, 115.6, 130.9, 130.9, 132.4, 163.2, 170.4, 170.7, 171.2, 193.9. ESI MS: 478.3 ([M + H]^+^). HR ESI MS: calcd for C_23_H_33_O_5_N_5_F 478.24602; found 478.24526.

#### *tert*-Butyl (*S*)-6-Diazo-2-((*S*)-2-(2-(dimethylamino)acetamido)-3-(3-fluorophenyl)propanamido)-5-oxohexanoate (**P16**)

General method B, starting material **9g** (392 mg); reaction time 16 h; mobile phase: DCM/MeOH, 20:1. Prodrug **P16** was isolated as a yellow amorphous compound (391 mg) in 82% yield. ^1^H NMR (401 MHz, CDCl_3_): δ 1.44 (s, 9H), 1.87–2.01 (m, 1H), 2.16 (s, 6H), 2.19–2.42 (m, 2H), 2.82 (d, *J* = 16.3, 1H), 2.98 (d, *J* = 16.3, 1H), 3.00–3.08 (m, 1H), 3.17 (dd, *J* = 14.0, 6.2, 1H), 4.32–4.39 (m, 1H), 4.65 (td, *J* = 8.1, 6.1, 1H), 5.28–5.33 (bs, 1H), 6.85–6.95 (m, 3H), 6.99 (d, *J* = 7.8, 1H), 7.19–7.26 (m, 1H), 7.59 (d, *J* = 8.2, 1H). ^13^C NMR (101 MHz, CDCl_3_): δ 27.3, 28.1, 36.4, 37.5, 37.6, 46.0, 52.6, 53.6, 53.9, 54.9, 63.0, 82.6, 113.9, 114.1, 116.2, 116.5, 125.0, 125.0, 130.1, 130.2, 139.2, 139.3, 161.7, 164.2, 170.4, 170.6, 171.2, 193.9. ESI MS: 478.3 ([M + H]^+^). HR ESI MS: calcd for C_23_H_33_O_5_N_5_F 478.24602; found 478.24527.

#### *tert*-Butyl (*S*)-6-Diazo-2-((*S*)-2-(2-(dimethylamino)acetamido)-3-(4-(trifluoromethyl)phenyl)propanamido)-5-oxohexanoate (**P17**)

General method B, starting material **9h** (442 mg); reaction time 2 h; mobile phase: DCM/MeOH, 30:1. Prodrug **P17** was isolated as a yellow solid (407 mg) in 77% yield. ^1^H NMR (401 MHz, CDCl_3_): δ 1.42 (s, 9H), 1.93 (dtd, *J* = 14.3, 8.0, 6.3, 1H), 2.14 (s, 6H), 2.06–2.18 (m, 1H), 2.20–2.42 (m, 2H), 2.81 (d, *J* = 16.3, 1H), 2.96 (d, *J* = 16.3, 1H), 3.07 (dd, *J* = 14.1, 8.0, 1H), 3.24 (dd, *J* = 14.1, 6.3, 1H), 4.34 (td, *J* = 7.7, 4.8, 1H), 4.72 (td, *J* = 8.1, 6.3, 1H), 5.28 (bs, 1H), 7.03 (d, *J* = 7.4, 1H), 7.32 (d, *J* = 8.4, 2H), 7.51 (d, *J* = 7.4, 2H), 7.59 (d, *J* = 8.3, 1H). ^13^C NMR (101 MHz, CDCl_3_): δ 27.05, 28.01 (3C), 36.35, 37.71, 45.93 (2C), 52.61, 53.61, 54.94, 62.96, 82.53, 125.51 (q, *J* = 3.7 Hz, 2C), 129.35 (q, *J* = 32.6 Hz), 129.78 (2C), 140.92, 140.93, 170.38, 170.52, 171.20, 193.89. ESI MS: 528.3 ([M + H]^+^). HR ESI MS: calcd for C_24_H_33_O_5_N_5_F_3_ 528.24283; found 528.24252.

#### *tert*-Butyl (*S*)-6-Diazo-2-(2-(2-(dimethylamino)acetamido)acetamido)-5-oxohexanoate (**P18**)

General method A, starting material **9i** (284 mg); solvent: DMF; reaction time 1.5 h; mobile phase: DCM/MeOH, 10:1 to 5:1. Prodrug **P18** was isolated after lyophilization (MeCN/H_2_O, 4:1; 50 mL) as light-yellow solid (237 mg) in 64% yield. ^1^H NMR (401 MHz, CDCl_3_): δ 1.46 (s, 9H), 1.99 (dtd, *J* = 14.3, 8.0, 6.5, 1H), 2.19 (dtd, *J* = 15.7, 8.5, 7.9, 5.2, 1H), 2.33 (s, 6H), 2.34–2.46 (m, 2H), 2.99 (d, *J* = 16.3, 1H), 3.06 (d, *J* = 16.3, 1H), 3.93 (dd, *J* = 16.6, 5.9, 1H), 4.02 (dd, *J* = 16.6, 5.9, 1H), 4.44 (td, *J* = 8.0, 4.6, 1H), 5.31 (bs, 1H), 6.76 (d, *J* = 7.6, 1H), 7.72 (bs, 1H). ^13^C NMR (101 MHz, CDCl_3_): δ 27.25, 28.05 (3C), 36.54, 42.75, 46.15 (2C), 52.52, 54.98, 62.99, 82.57, 169.00, 170.74, 171.73, 194.01. ESI MS: 392.2 ([M + Na]^+^). HR ESI MS: calcd for C_16_H_27_O_5_N_5_Na 392.19044; found 392.19016.

#### *tert*-Butyl (*S*)-6-Diazo-2-((*S*)-2-(2-(dimethylamino)acetamido)propanamido)-5-oxohexanoate (**P19**)

General method A, starting material **9j** (298 mg); solvent: DMF; reaction time 2.5 h; mobile phase: DCM/MeOH, 10:1. Prodrug **P19** was isolated as light-yellow amorphous compound (268 mg) in 70% yield. ^1^H NMR (401 MHz, *d*_6_-DMSO): δ 1.24 (d, *J* = 7.0, 3H), 1.39 (s, 9H), 1.78 (ddd, *J* = 14.3, 9.4, 6.0, 1H), 1.96 (dq, *J* = 14.0, 7.1, 1H), 2.21 (s, 6H), 2.33–2.42 (m, 2H), 2.79–2.93 (m, 2H), 4.09 (ddd, *J* = 9.1, 7.3, 5.1, 1H), 4.36 (p, *J* = 7.1, 1H), 6.06 (bs, 1H), 7.73 (d, *J* = 7.9, 1H), 8.26 (d, *J* = 7.6, 1H). ^13^C NMR (101 MHz, CDCl_3_): δ 18.2, 27.3, 28.1 (3C), 36.5, 46.1 (2C), 48.5, 52.6, 63.1, 70.6, 82.5, 170.7, 171.0, 172.2, 194.2. ESI MS: 384.2 ([M + H]^+^). HR ESI MS: calcd for C_17_H_30_O_5_N_5_ 384.22415; found 384.22401.

#### *tert*-Butyl (*S*)-6-Diazo-2-((*S*)-2-(2-(dimethylamino)acetamido)-4-methylpentanamido)-5-oxohexanoate (**P20**)

General method B, starting material **9k** (340 mg); reaction time 5 h; mobile phase: DCM/MeOH, 10:1. Prodrug **P20** was isolated as a yellow amorphous compound (306 mg) in 72% yield. ^1^H NMR (401 MHz, CDCl_3_): δ 0.89 (d, *J* = 6.1, 3H), 0.92 (d, *J* = 6.1, 3H), 1.42 (s, 9H), 1.51–1.71 (m, 3H), 1.91 (dtd, *J* = 14.4, 8.4, 6.1, 1H), 2.08–2.21 (m, 1H), 2.28 (s, 6H), 2.20–2.45 (m, 2H), 2.96 (d, *J* = 3.1, 2H), 4.33–4.46 (m, 2H), 5.34 (bs, 1H), 6.90 (d, *J* = 7.7, 1H), 7.47 (d, *J* = 8.5, 1H). ^13^C NMR (101 MHz, CDCl_3_): δ 21.90, 23.08, 24.87, 27.38, 28.03 (3C), 29.74, 36.46, 41.00, 45.99, 51.39, 52.44, 54.85, 62.98, 82.36, 170.63, 170.93, 172.02, 194.00. ESI MS: 426.3 ([M + H]^+^). HR ESI MS: calcd for C_20_H_36_O_5_N_5_ 426.27110; found 426.27057.

#### *tert*-Butyl (*S*)-6-Diazo-2-((*S*)-2-(2-(dimethylamino)acetamido)-4,4-dimethylpentanamido)-5-oxohexanoate (**P21**)

General method B, starting material **9l** (354 mg); reaction time 2.5 h; mobile phase: DCM/MeOH, 20:1 to 15:1. Prodrug **P21** was isolated as a yellow amorphous compound (404 mg) in 92% yield. ^1^H NMR (401 MHz, CDCl_3_): δ 0.90 (d, *J* = 1.7, 9H), 1.33–1.50 (m, 10H), 1.79–1.95 (m, 2H), 2.05–2.17 (m, 1H), 2.19–2.38 (m, 8H), 2.90 (s, 2H), 4.24–4.46 (m, 2H), 5.33 (bs, 1H), 6.83–6.94 (m, 1H), 7.43 (d, *J* = 8.3, 1H). ^13^C NMR (101 MHz, CDCl_3_): δ 28.0, 29.7, 30.5, 45.4, 46.1, 50.6, 52.4, 53.5, 63.0, 82.2, 82.3, 170.5, 170.8, 172.3, 193.9. ESI MS: 440.3 ([M + H]^+^). HR ESI MS: calcd for C21H_38_O_5_N_5_ 440.28675; found 440.28632.

### Metabolic Stability

Prodrugs were screened for metabolic stability in plasma and intestinal tissue homogenates in compliance with our previously reported methods.^25,46^ Briefly, intestinal tissue was homogenized over ice via probe sonication with 1:9 tissue to potassium phosphate buffer (0.1 M) conditions. Prodrugs were spiked in plasma or tissue homogenate at 10 μM final concentration with 0.2% DMSO v/v and incubated in triplicate for 0 and 60 min time points. At each time point, 100 μL of the sample was precipitated with 300 μL of methanol containing internal standard (IS; losartan, 0.5 μM). Precipitated samples were vortexed (30 s) and centrifuged at 10000*g* for 10 min at 4 °C. After centrifugation, 100 μL of supernatant was aliquoted and submitted for analysis. Prodrug disappearance over time was monitored by liquid chromatography–mass spectrometry (LC–MS) through peak area ratios between the analyte and internal standard.

Chromatographic analysis was performed on a Dionex ultra-high-performance LC system coupled with a Q Exactive Focus Orbitrap mass spectrometer (Thermo Fisher Scientific Inc., Waltham, MA). Separation was achieved using an Agilent Eclipse Plus column (100 × 2.1 mm i.d.; maintained at 35 °C) packed with a 1.8 μm C18 stationary phase. The mobile phase used was composed of 0.1% formic acid in acetonitrile and 0.1% formic acid in water with gradient elution, starting with 2.5% organic phase (from 0 to 0.25 min) and linearly increasing to 99% (from 0.25 to 1.25 min) and re-equilibrating to 2.5% by 4 min. The total run time for each analyte was 5 min. Pumps were operated at a flow rate of 0.4 mL/min. The mass spectrometer controlled by Xcalibur software 4.0.27.13 (Thermo Scientific) was operated with an HESI ion source in positive ionization mode for all compounds. Compounds were identified in the full-scan mode with *m*/*z* ranging from 75 to 1125.

### Pharmacokinetics of Prodrugs in C57BL/6/CES1^–/–^ Mice

C57BL/6/CES1^–/–^ mice were obtained as a gift from the United States Army Medical Research Institute of Chemical Defense, Maryland, USA; breeding was performed in the Johns Hopkins animal facility and was conducted according to protocols reviewed and approved by the Johns Hopkins Institutional Animal Care and Use Committee in compliance with the Association for Assessment and Accreditation of Laboratory Animal Care International (AAALAC) and the Public Health Service Policy on the Humane Care and Use of Laboratory Animals (PHS Policy). Male and female C57BL/6 mice (weighing between 25 and 30 g) 6–8 weeks of age were used for the study. The animals were maintained on a 12 h light–dark cycle with *ad libitum* access to food (certified laboratory food: Teklad 18% Protein Extruded Rodent Diet) and water. EL4 mouse lymphoma cells were obtained as a gift from the laboratory of Dr. Jonathan Powell (Johns Hopkins University, Baltimore, MD) and maintained in RPMI 1640 medium 1X (Corning, cat. no. 10-040-CV) with 10% (v/v) fetal bovine serum (Corning, cat. no. 35-011-CV), 1% (v/v) antimycotic/antibiotic (Corning, cat. no. 30-004-CI), 2 mM l-glutamine (Corning, cat. no. 25-005-CI), and 10 mM HEPES (Corning, cat. no. 25-060-CI) in a 5% (v/v) CO_2_ and 95% (v/v) air incubator prior to subcutaneous (SC) injection (1 × 10^6^ cells in 0.2 mL of phosphate-buffered saline) on the flank of each mouse.

Pharmacokinetic study was performed after tumors grew to a mean volume of around 400 mm^3^. Prior to dosing, the interscapular region was wiped with alcohol gauze. For single time point PK, prodrugs were dissolved immediately prior to dosing in ethanol:Tween 80:saline (5:10:85 v/v/v) and administered to mice as a single SC dose of 1 mg/kg DON equivalent. The mice were euthanized with carbon dioxide at 30 min post drug administration, blood samples (∼0.8 mL) were collected in heparinized microtubes by cardiac puncture, and jejunum as well as tumors were removed and flash frozen on dry ice. Blood samples were centrifuged at a temperature of 4 °C at 3000*g* for 10 min. All samples were maintained chilled throughout processing. Plasma samples (∼300 μL) were collected in polypropylene tubes and stored at −80 °C until bioanalysis. Flash-frozen jejunum and tumor samples were also stored at −80 °C until bioanalysis.

For complete pharmacokinetic evaluation of **P11**, similar methods (including vehicle) as described above were utilized except plasma, tumor, and GI sample collection for pharmacokinetics was conducted at 0–6 h post dose.

#### Bioanalysis of DON and Intact Prodrug **P11**

Plasma concentration levels of **P11** were measured by precipitating 20 μL of plasma sample with 100 μL of methanol containing internal standard (losartan, 0.5 μM), followed by vortex mixing for 30 s and then centrifugation at 16000*g* for 5 min at 4 °C. Jejunum and tumor tissues were diluted 1:5 w/v with methanol containing losartan (0.5 μM) and homogenized, followed by vortex mixing and centrifugation at 16000*g* for 5 min at 4 °C.

Chromatographic analysis was performed on an Agilent ultra-high-performance LC system coupled with an Agilent 6520 QTOF mass spectrometer (Agilent, Santa Clara, CA). Separation was achieved using an Agilent Eclipse Plus column (100 × 2.1 mm i.d.; maintained at 35 °C) packed with a 1.8 μm C18 stationary phase. The mobile phase used was composed of 0.1% formic acid in acetonitrile and 0.1% formic acid in water with gradient elution, starting with 2.5% organic phase (from 0 to 0.5 min) and linearly increasing to 95% (from 0.5 to 5 min), maintaining for 1 min, and re-equilibrating to 2.5% by 7 min. Pumps were operated at a flow rate of 0.3 mL/min. Standards and QCs were prepared (0.01–100 nmol/mL) in naïve matched matrixes. **P11** concentrations were determined by the AUC of high-resolution extracted chromatograms of **P11** divided by internal standard (460.2554 *m*/*z*/423.1695 *m*/*z*).

Bioanalysis of DON in pharmacokinetic samples, plasma, and tissue homogenates was conducted as we have previously described.^19,25^ Briefly, standards and QCs were prepared in respective matrixes. Standards, QCs, and plasma samples (20 μL) were precipitated with 100 μL of methanol containing internal standard (5 μM glutamate-*d*_5_) in low-retention microcentrifuge tubes. Jejunum and tumor samples were processed by adding 5 μL of methanol containing internal standard per each milligram of the tissue sample and mechanically homogenized with three Spex 2150 stainless-steel beads operated in a Geno/Grinder for 3 min at 1500 rpm. For plasma, jejunum, and tumor, the mixture was vortex mixed and centrifuged at 16000*g* for 5 min at 4 °C. The supernatant (100 μL) was evaporated to dryness under vacuum at 45 °C for 1 h. Dried samples were derivatized using dabsyl chloride, as described above. Calibration curves were constructed over the range 0.03–100 nmol/mL for DON in plasma, jejunum, and tumor tissues.

Pharmacokinetic parameters of **P11** and DON were calculated using noncompartmental analysis by PKanalix (PKanalix, Monolix Suite 2023R1, Lixoft, France). Parameters reported include maximum plasma and tissue concentration (*C*_max_), time to *C*_max_ (*T*_max_), and area under the plasma and tissue concentration time curve (AUC). AUC was calculated to the last quantifiable sample (AUC_0–last_) by use of the log–linear trapezoidal rule.

#### Glutamine and FGAR Quantification

Glutamine and FGAR were extracted from the tumor by protein precipitation. Per milligram of tissue, 5 μL of methanol containing 10 μmol/L deuterated *N*-acetyl aspartic acid and deuterated glutamate (internal standards) was added. Tissue samples were homogenized as described above and centrifuged (16000*g*, 5 min). Standard concentration curves of glutamine and FGAR in untreated plasma and tumor tissues were prepared (separately). For FGAR quantification, supernatants (2 μL) were injected and separated on an UltiMate 3000 UHPLC coupled to a Q Exactive Focus Orbitrap mass spectrometer. Samples were separated on an Agilent Eclipse Plus C18 RRHD (1.8 μm) 2.1 × 100 mm column. The mobile phase consisted of 8 mmol/L dimethylhexylamine (DMHA) + 0.005% formic acid in water, pH 9 (A), and 8 mmol/L DMHA in acetonitrile (B). Separation was achieved at a flow rate of 0.4 mL/min using a gradient run. Quantification was performed in full MS negative mode. Data were acquired and quantified with Xcalibur software.

Glutamine analysis took place on an Agilent 1290 UPLC coupled to an Agilent 6520 quadrupole time-of-flight mass spectrometer. Samples (2 μL) were injected and separated on a Waters Acquity UPLC BEH Amide 1.7 μm 2.1 × 100 mm HILIC column with a flow rate of 0.3 mL/min. The mobile phases consisted of A (water +0.1% formic acid) and B (acetonitrile + 0.1% formic acid). The mass spectrometer was run in positive ion mode. Standard curves for FGAR and glutamine were fitted using a blank subtraction method^47^ to compensate for the presence of endogenous analyte levels in naïve matrixes.

### Human Tumor Cell to Plasma Partitioning Assay

Human tumor cell to plasma partitioning assays were conducted as we have previously described.^25^ Briefly, P493B lymphoma cells were grown at 37 °C, in a humidified atmosphere with 5% CO_2_. Cell confluency was estimated, and cells were harvested after achieving >80% confluency and centrifuged at 200*g* for 5 min at 25 °C. The obtained cell pellet was resuspended in 20 mL of Dulbecco’s phosphate-buffered saline (DPBS; Gibco, USA, cat. no. 14-190-144) maintained at 37 °C, and cell count was determined using an automated cell counter (Bio-Rad, USA). Cell suspension in DPBS was further centrifuged at 200*g* for 5 min at 25 °C, and the cell pellet was resuspended in human plasma (Innovative Research, USA) for partitioning assessment. The final cell density after resuspending in plasma was 10 million cells/mL of plasma. Partitioning assessment was performed in triplicate. Preincubated (37 °C for 5 min) cell–plasma suspension was spiked with DRP-104 or **P11** at a final concentration of 20 μM and incubated at 37 °C for 1 h. Following incubation, a 1 mL aliquot of cell–plasma suspension was centrifuged at 1000*g* for 5 min at 4 °C, and supernatant plasma as well as the cell pellet was collected and stored at −80 °C until bioanalysis. Both plasma and cell pellet fractions were analyzed for intact prodrug and DON levels.

For bioanalysis, cell pellets were resuspended in water and the total weight of cells was noted. The calibration curves (0.03–100 nmol/mL) were prepared in both human plasma and untreated P493B cells. A 50 μL volume of cell suspension/plasma was precipitated with 250 μL of methanol containing internal standards (glutamate-*d*_5_, 5 μM, and losartan, 0.5 μM). Samples were briefly vortexed for 30 s and centrifuged at 10000*g* for 10 min at 4 °C. DON, **P11**, and DRP-104 analyses were conducted as described above and previously.^25^

### Cell Viability Assay

Cell proliferation assays were performed as we have previously described.^19^ Briefly, P493B lymphoma cells were plated in 96-well plates at a density of 20 000 cells/well in a final volume of 100 μL of growth media. **P11**, or DRP-104, was added to cells in half-log serial dilutions with a final concentration of 0.2% DMSO. Cells were allowed to proliferate for 72 h, and thereafter, 20 μL of CellTiter 96 AQueous (Promega no. 3580) was added per well and incubated for 2 h. Absorbance was measured at 490 nm. Relative cell viability was calculated from the difference between untreated cell wells (100%) and media well without cells (0%).

### Solubility Assay

The aqueous solubility was determined as previously reported with minor modifications.^48^ Briefly, an excess amount of **P11** or DRP-104 was added to PBS buffer (pH 7.4) and sonicated for 1 h at 37 °C to ensure saturation. After 1 h, the solution was filtered (0.45 μm PTFE), and the filtrate was diluted appropriately, and the concentration of each analyte was determined via LC–MS. Chemical stabilities of DRP-104 and **P11** were done as previously described^18^ with modifications. For chemical stability, prodrugs were spiked (10 μM) in buffer at pH 7.4 and incubated at 37 °C for 24 h. Prodrug disappearance was monitored using the developed HRMS methods.

## Supporting Information Available

^1^H and ^13^C NMR spectra, HPLC chromatograms of all final prodrugs, table of clogD_7.4_ values for all prodrugs, aqueous stability of DRP 104 and **P11** (PDF)
Molecular formula strings and associated biological data (CSV)

## Abbreviations Used

AUC: area under the curve
BLQ: below limit of quantification
CES1: carboxylesterase 1
CES1^–/–^: carboxylesterase 1 knockout
DCC: *N*,*N*′-dicyclohexylcarbodiimide
DIPEA: *N*,*N*-diisopropylethylamine
DMAP: 4-dimethylaminopyridine
DON: 6-diazo-5-oxo-l-norleucine
FDA: Food and Drug Administration
FGAR: formylglycinamide ribonucleotide
Fmoc: 9-fluorenylmethyloxycarbonyl
GI: gastrointestinal
HATU: 1-[bis(dimethylamino)methylene]-1*H*-1,2,3-triazolo[4,5-*b*]pyridinium 3-oxid hexafluorophosphate
HRMS: high resolution mass spectrometry
LiHMDS: lithium bis(trimethylsilyl)amide
MET ID: metabolite identification
WT: wild type
