# Supplementary material for: Discovery of tert-Butyl Ester Based 6-Diazo-5-oxo-l-norleucine Prodrugs for Enhanced Metabolic Stability and Tumor Delivery
Source: J Med Chem. 2023 Nov 10;66(22):15493–510. doi: 10.1021/acs.jmedchem.3c01681 (PMC10683027; doi:10.1021/acs.jmedchem.3c01681)
Supplement: Supplementary file 1 — jm3c01681_si_001.pdf [file jm3c01681_si_001.pdf]

Supporting information

Discovery of tert-butyl ester-based DON prodrugs for enhanced metabolic stability and tumor delivery

Authors: Kateřina Novotná[°1,8,9], Lukáš Tenora[°1,8], Eva Prchalová[1,8], James Paule[1], Jesse Alt[1], Vijay Veeravalli[1], Jenny Lam[1], Ying Wu[1], Ivan Šnajdr[8], Sadakatali Gori[1], Vijaya Saradhi Mettu[1], Takashi Tsukamoto[1], Pavel Majer[8*], Barbara S. Slusher[1,2,3,4,5,6.7*] and Rana Rais[1,2,4*]

[1]Johns Hopkins Drug Discovery, Departments of [2]Neurology, [3]Psychiatry and Behavioral Sciences, [4]Pharmacology and Molecular Sciences, [5]Neuroscience, [6]Medicine, [7]Oncology, Johns Hopkins School of Medicine, Baltimore, Maryland 21205, U.S.A.

[8]Institute of Organic Chemistry and Biochemistry, Academy of Sciences of the Czech Republic v.v.i., Prague, 16600, Czech Republic

[9]Department of Organic Chemistry, Charles University, Faculty of Science, Prague, 12800, Czech Republic

°*these authors have equally contributed to this manuscript*

Address Correspondence to:

*Rana Rais, PhD, Johns Hopkins Drug Discovery, 855 North Wolfe Street, Baltimore, Maryland, USA 21205, Phone: 410-502-0497, Fax: 410-614-0659, E-mail: rrais2@jhmi.edu

*Barbara S. Slusher, PhD, Johns Hopkins Drug Discovery, 855 North Wolfe Street, Baltimore, Maryland, USA 21205, Phone: 410-960-6162, Fax: 410-614-0659, E-mail: bslusher@jhmi.edu

*Pavel Majer, CSc, Institute of Organic Chemistry and Biochemistry, Academy of Sciences of the Czech Republic vvi, Flemingovo nam. 2, 166 10 Prague, Czech Republic, Phone: +420-220-183-125, E-mail: majer@uochb.cas.cz

Table of contents

1. ^{1}H and ^{13}C NMR spectra of prodrugs **P1-P21** S2 – S23
2. HPLC traces of prodrugs **P1-P21** S24 – S30
3. Table of clog$D_{7.4}$ values for prodrugs **P1-P21** S31
4. Aqueous stability of DRP 104 and **P11** S32

^{1}H and ^{13}C NMR spectra of prodrugs P1-P21:

^{1}H and ^{13}C NMR Spectra of compound **P1**.

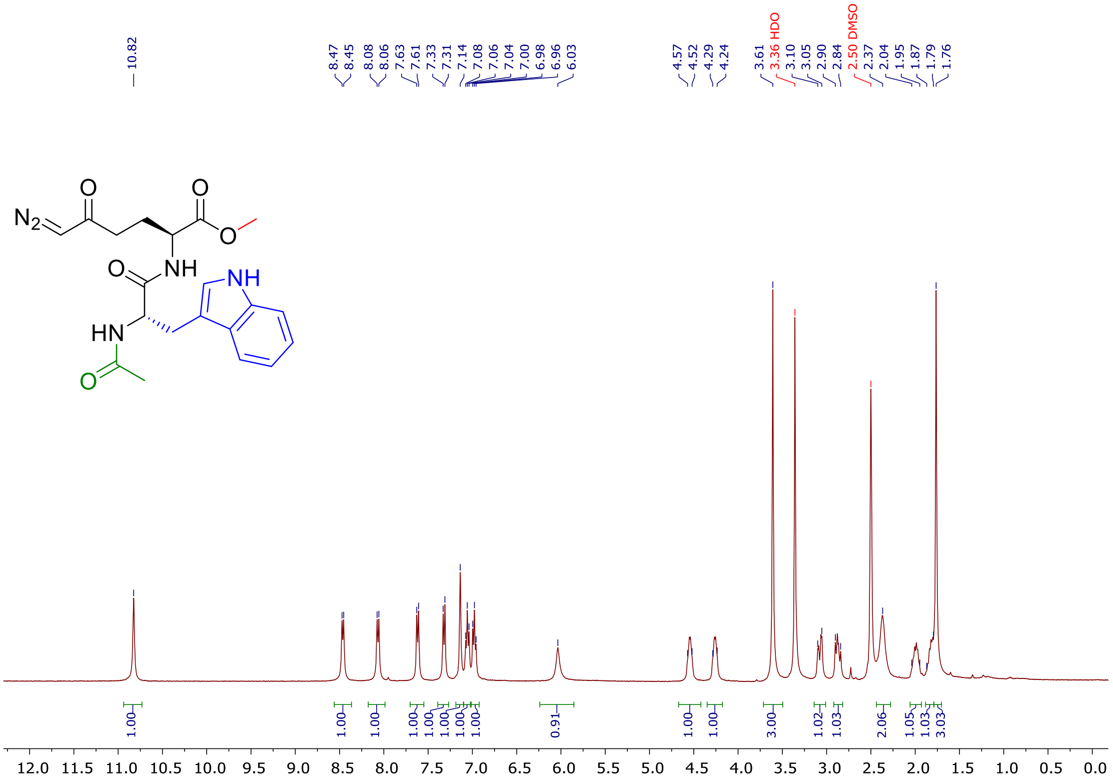

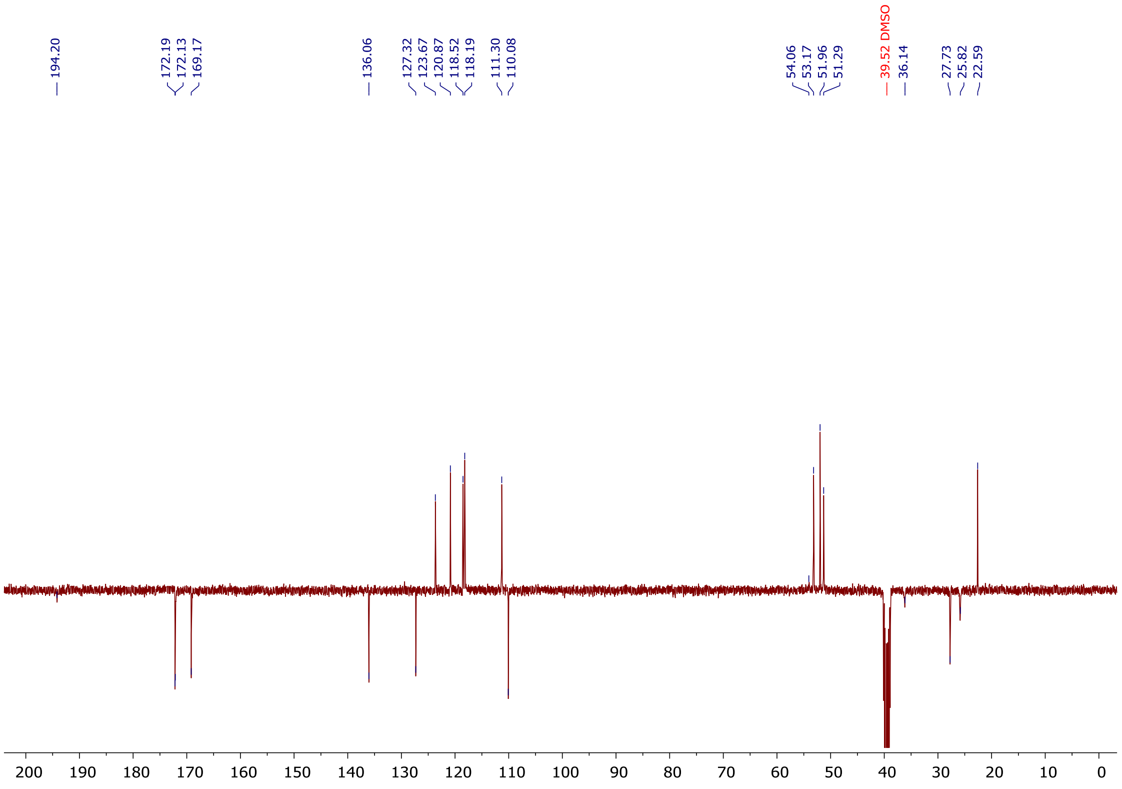

^{1}H and ^{13}C NMR Spectra of compound **P2**.

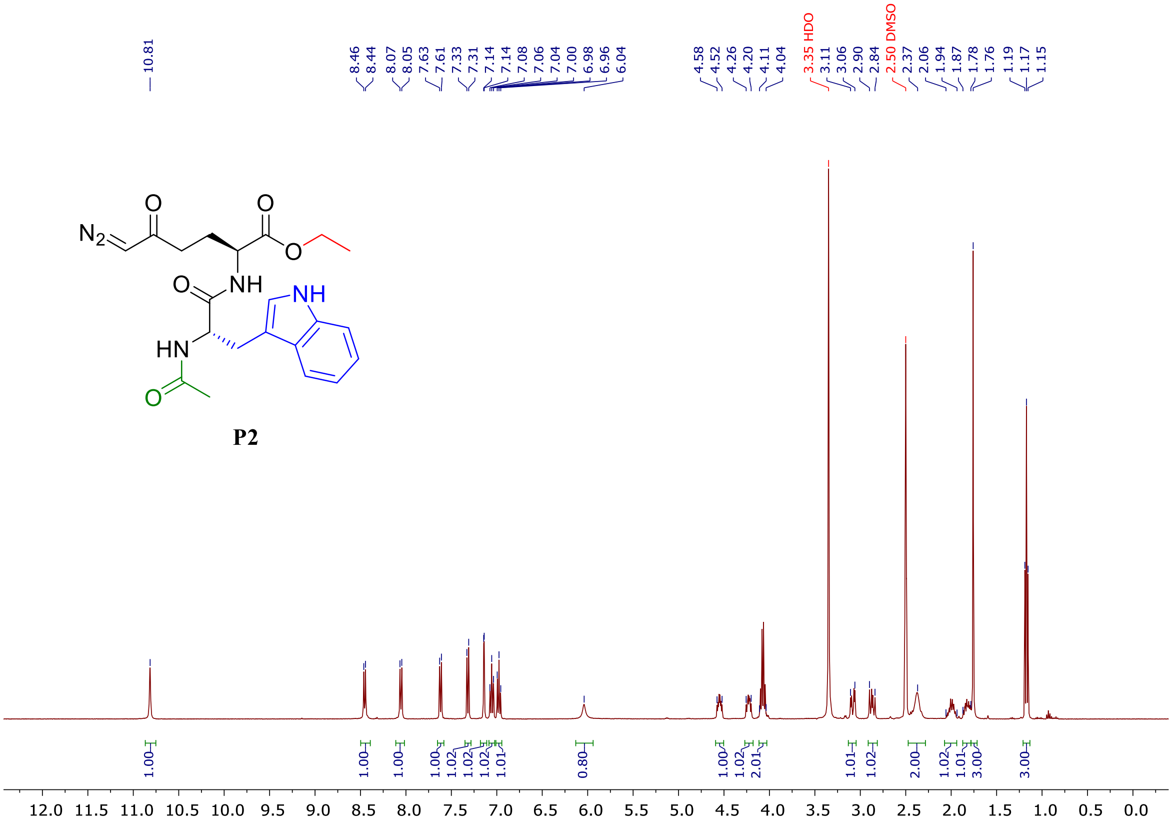

P2

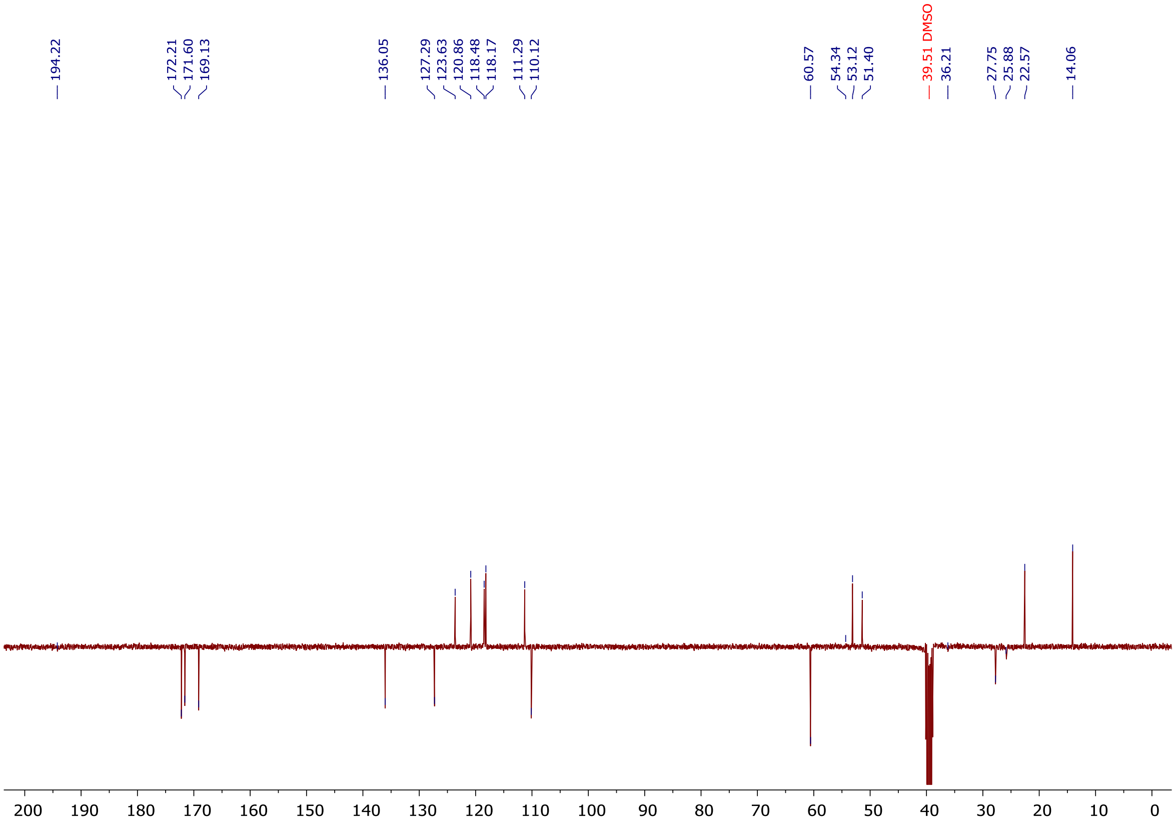

^{1}H and ^{13}C NMR Spectra of compound **P3**.

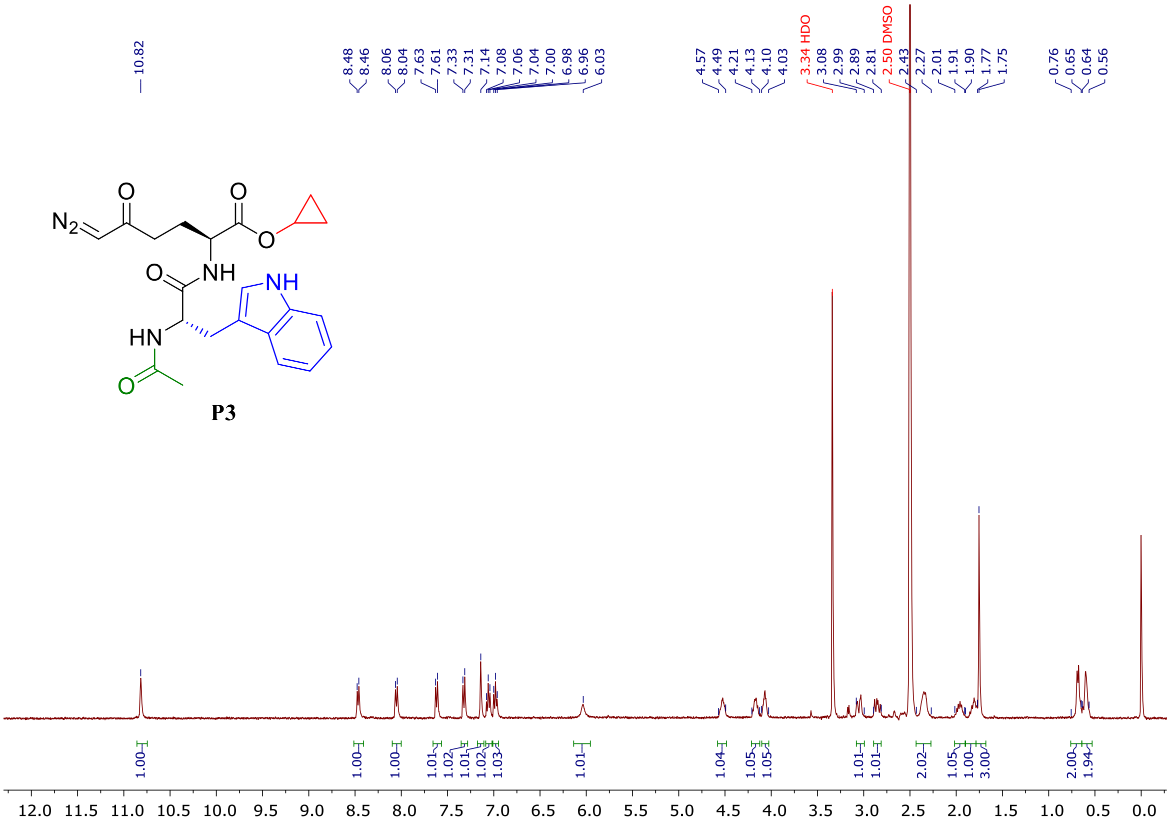

P3

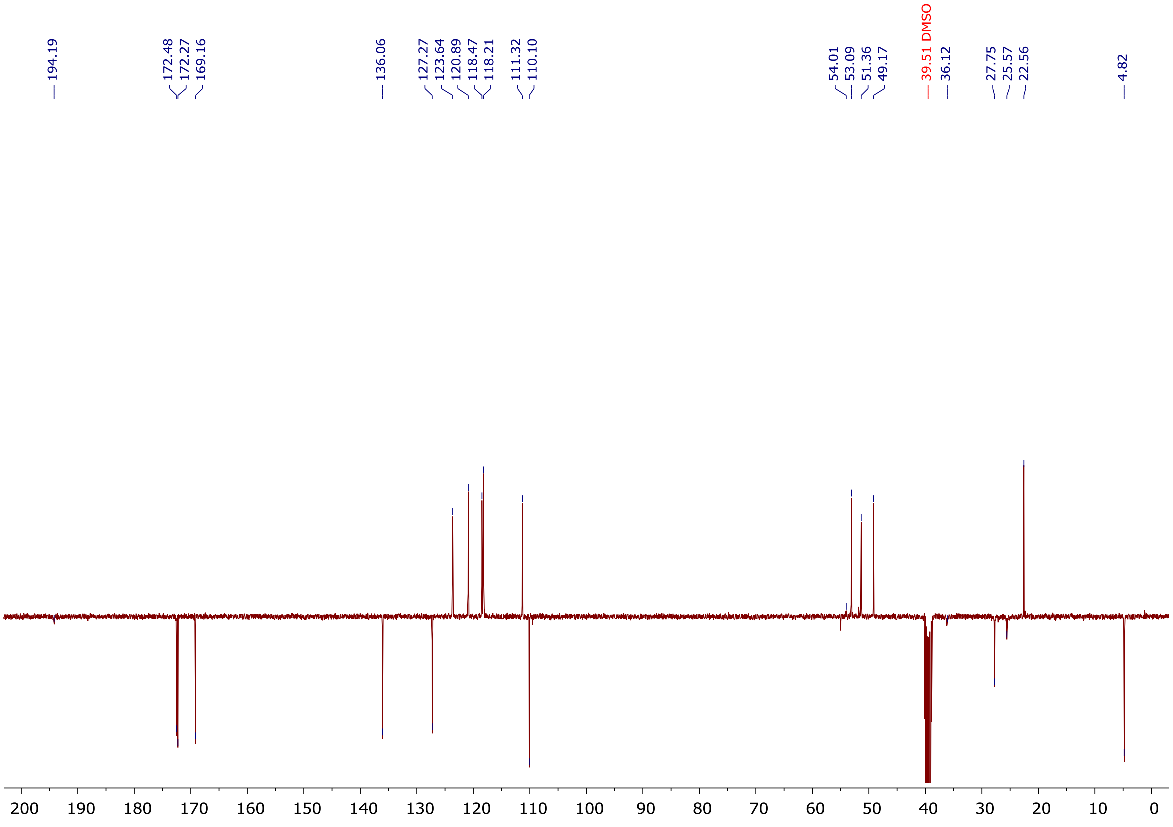

^{1}H and ^{13}C NMR Spectra of compound **P4**.

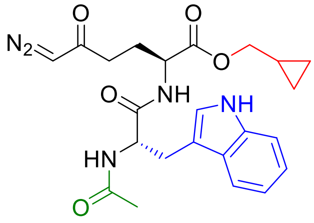

P4

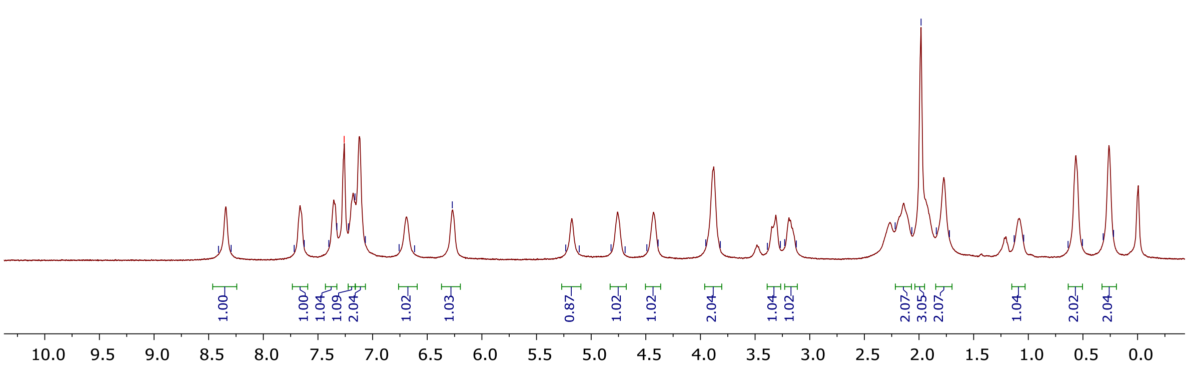

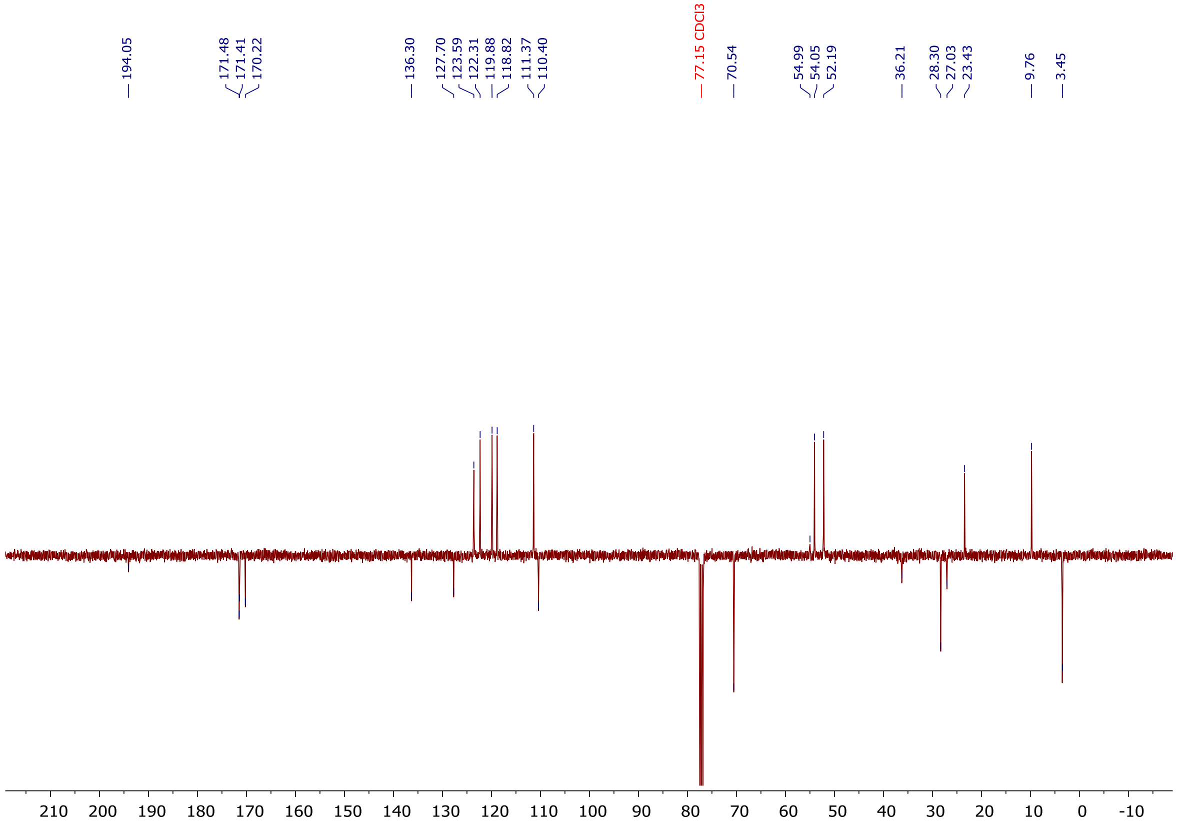

^{1}H and ^{13}C NMR Spectra of compound **P5**.

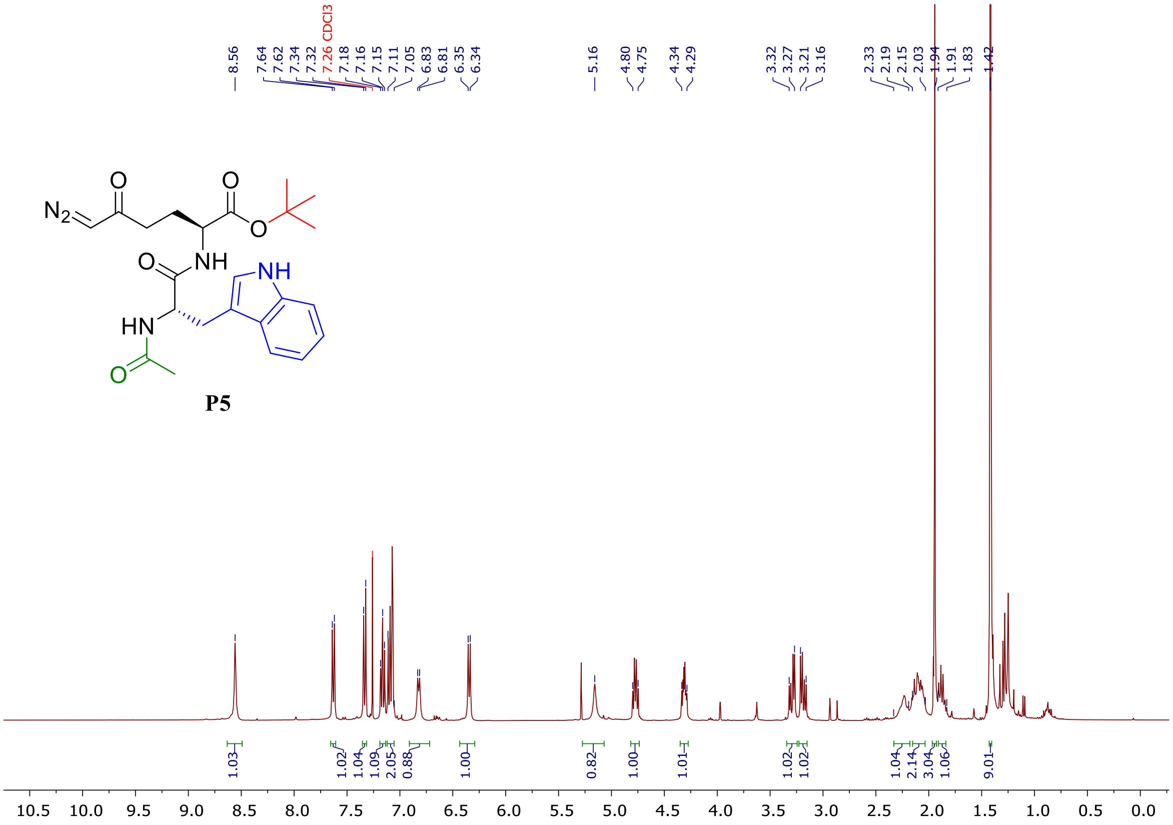

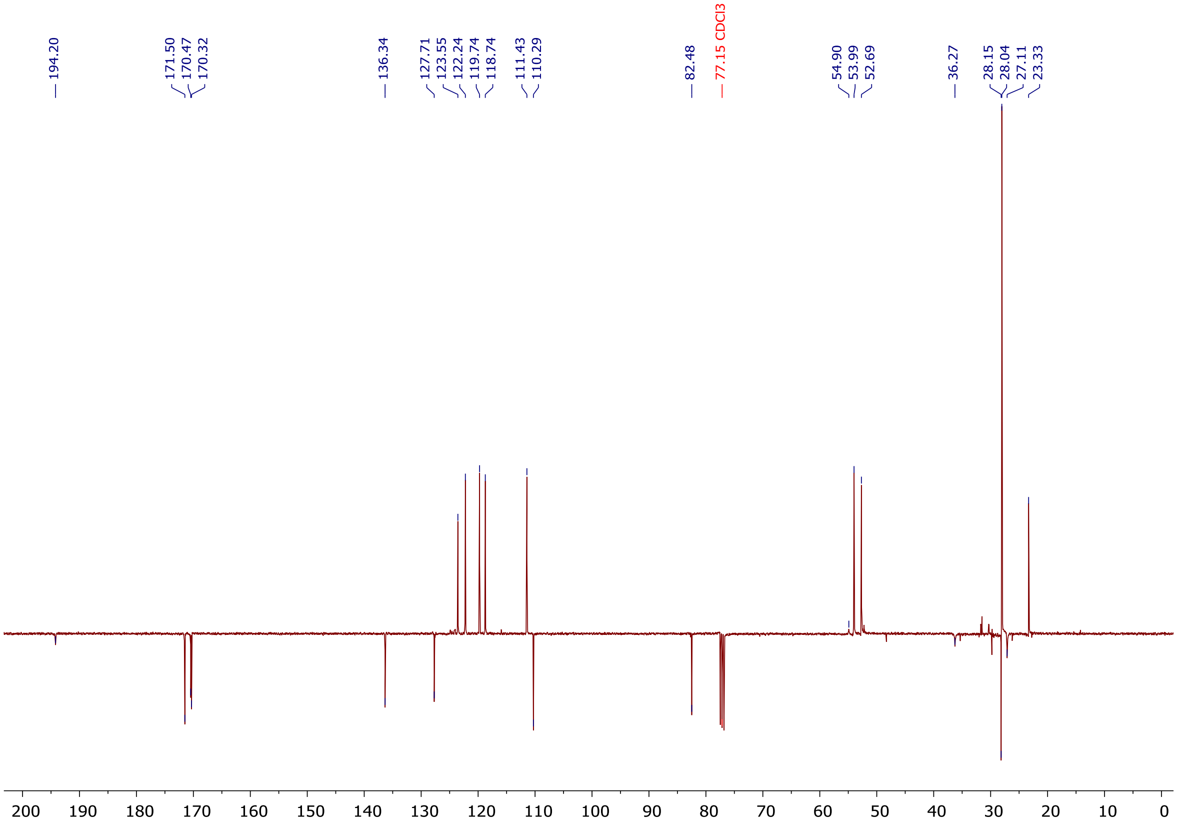

^{1}H and ^{13}C NMR Spectra of compound **P6**.

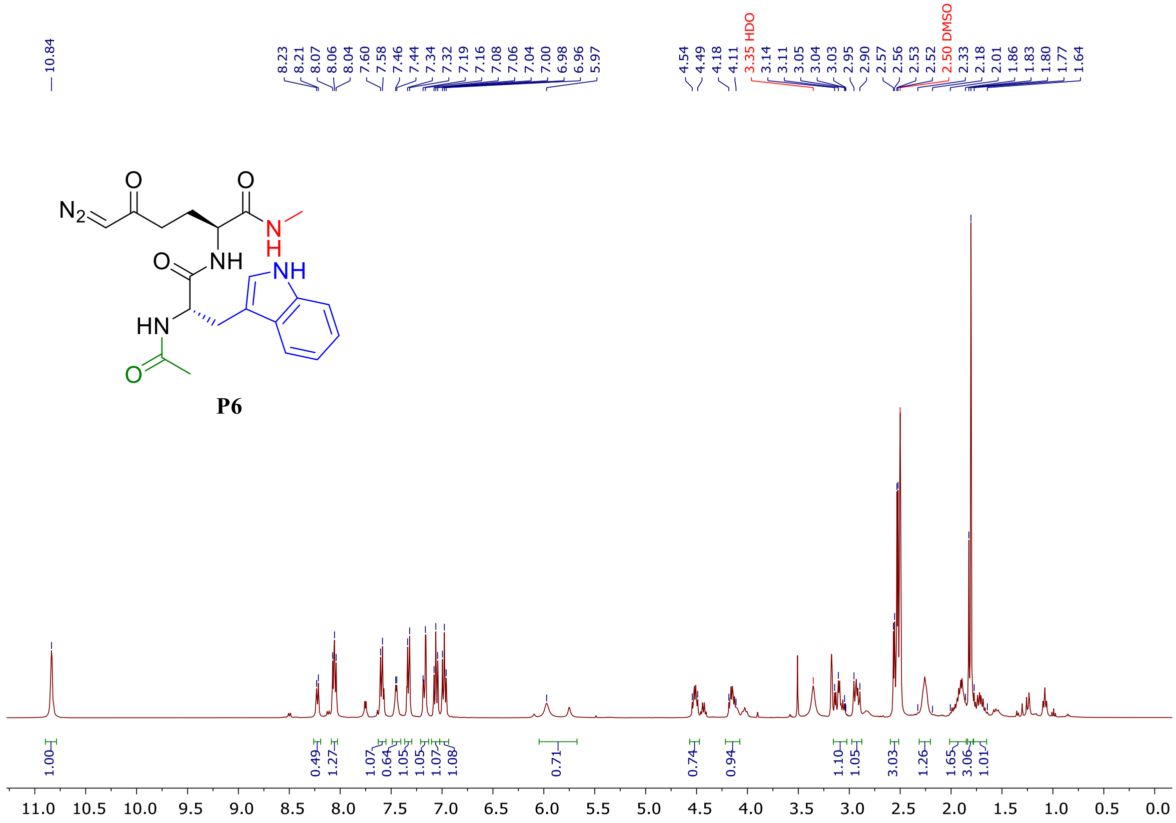

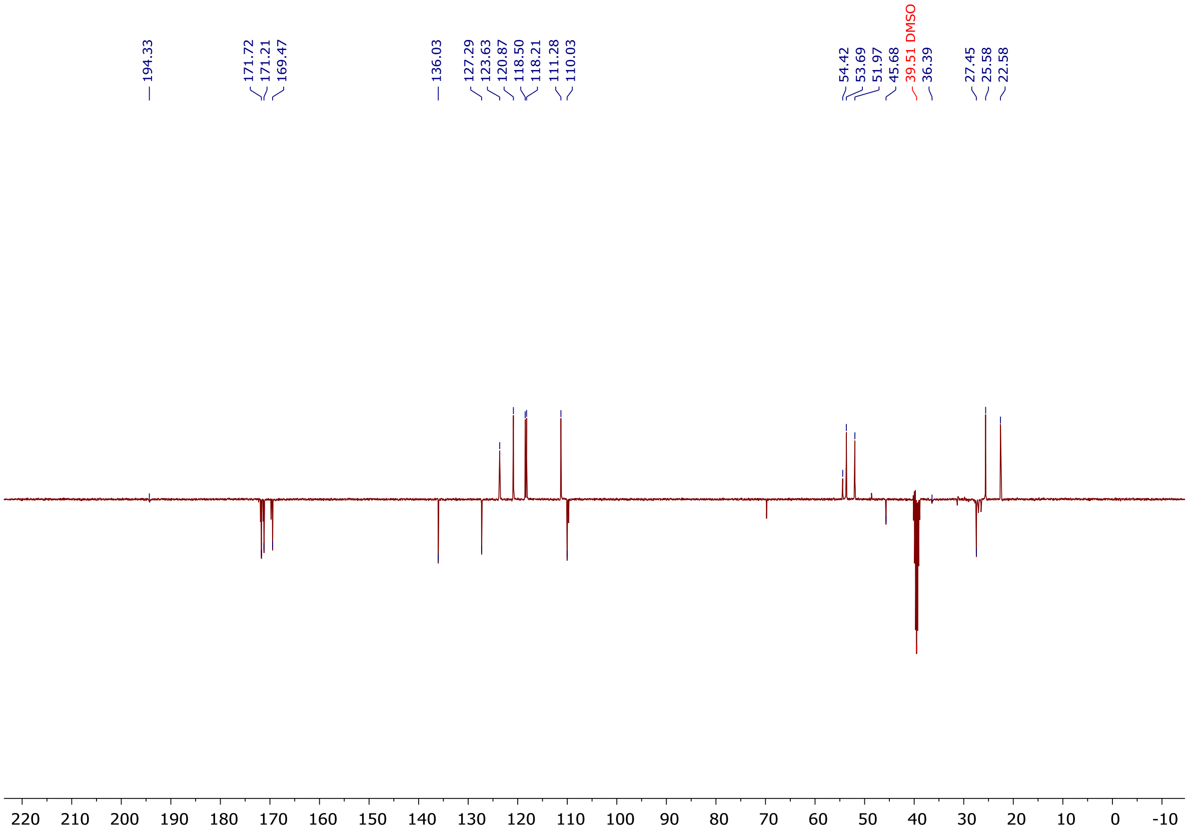

^{1}H and ^{13}C NMR Spectra of compound **P7**.

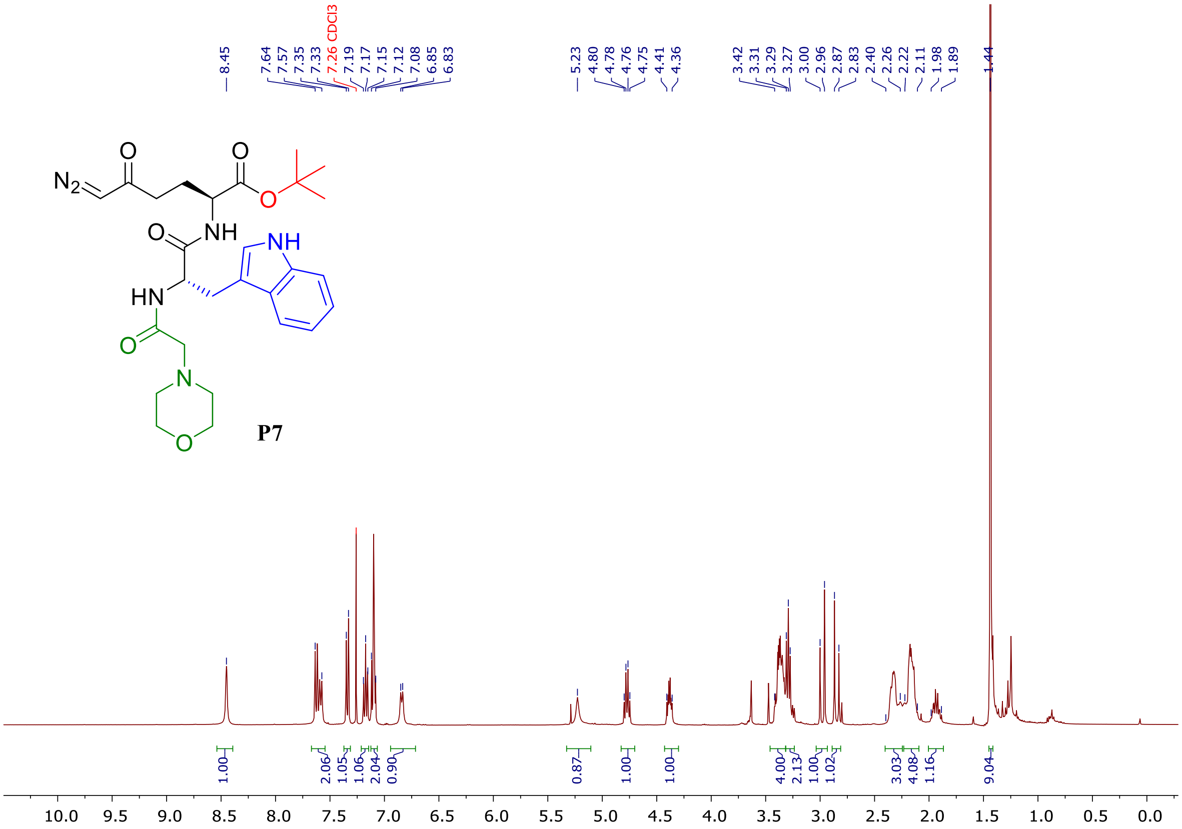

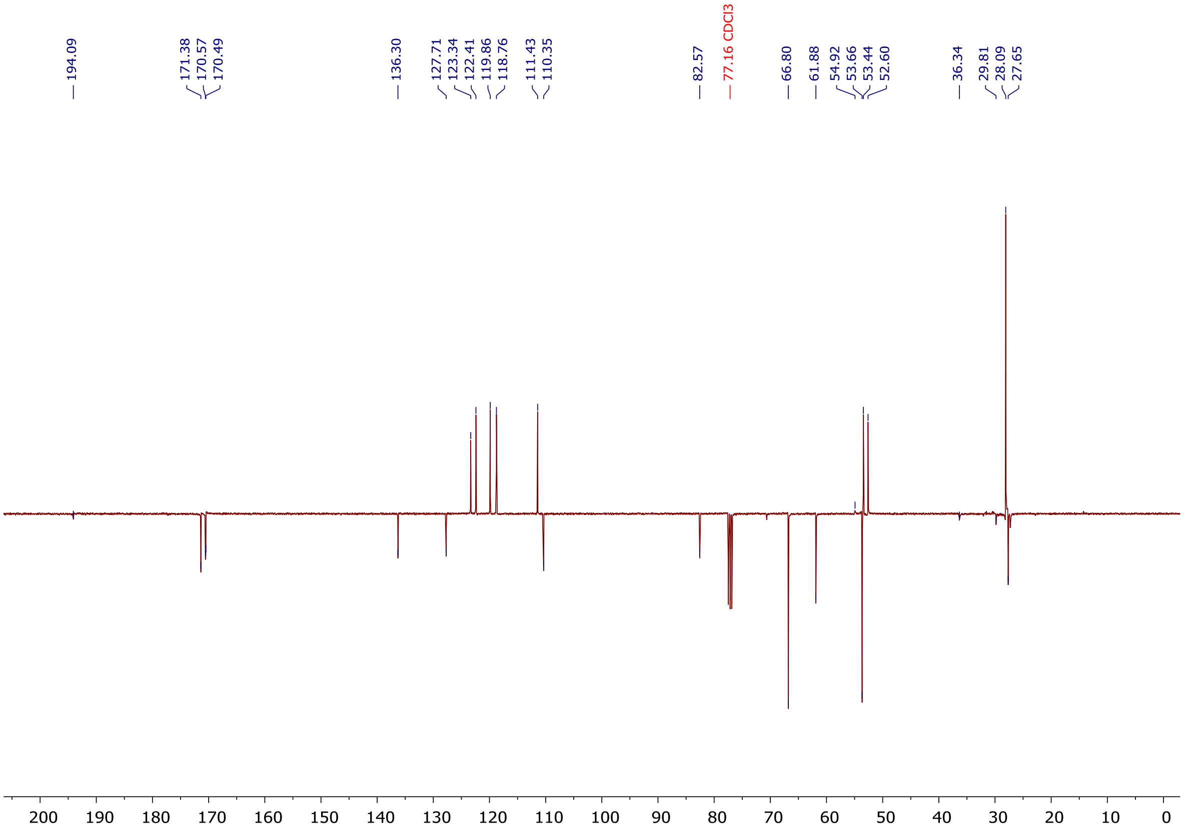

^{1}H and ^{13}C NMR Spectra of compound **P8**.

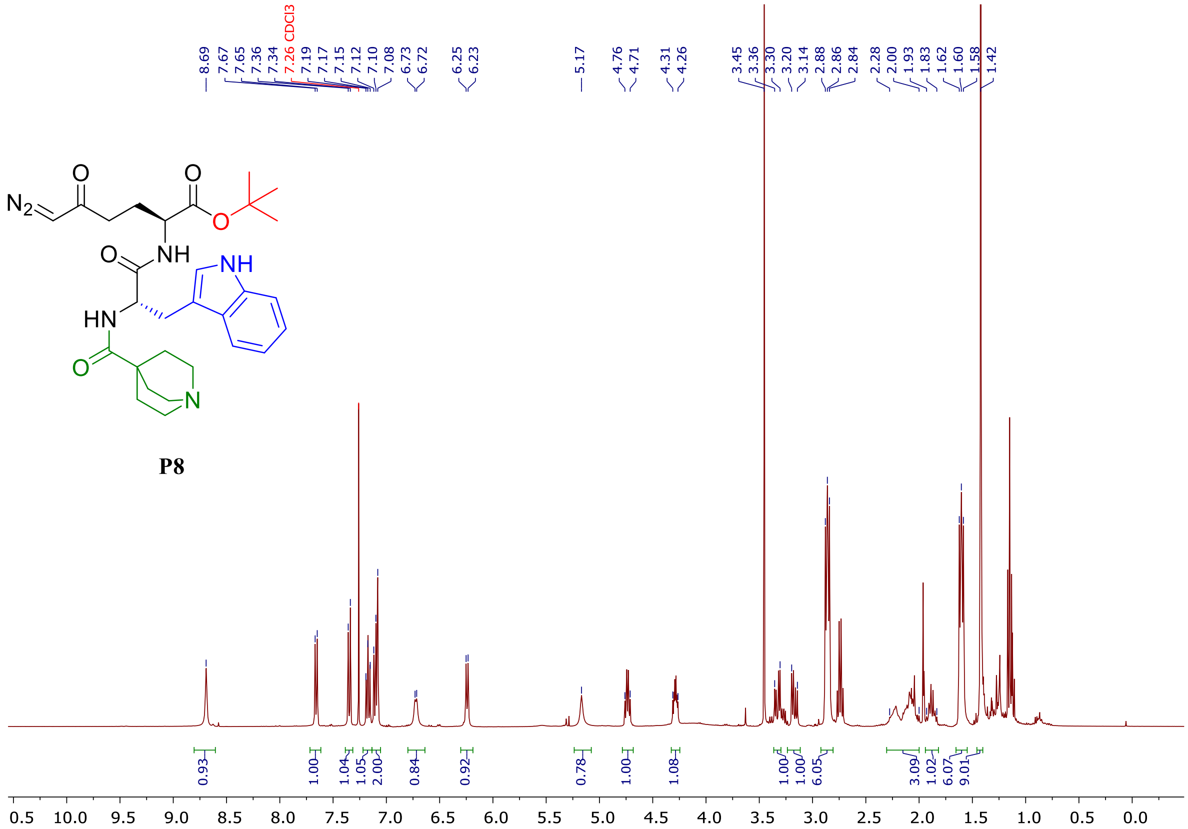

P8

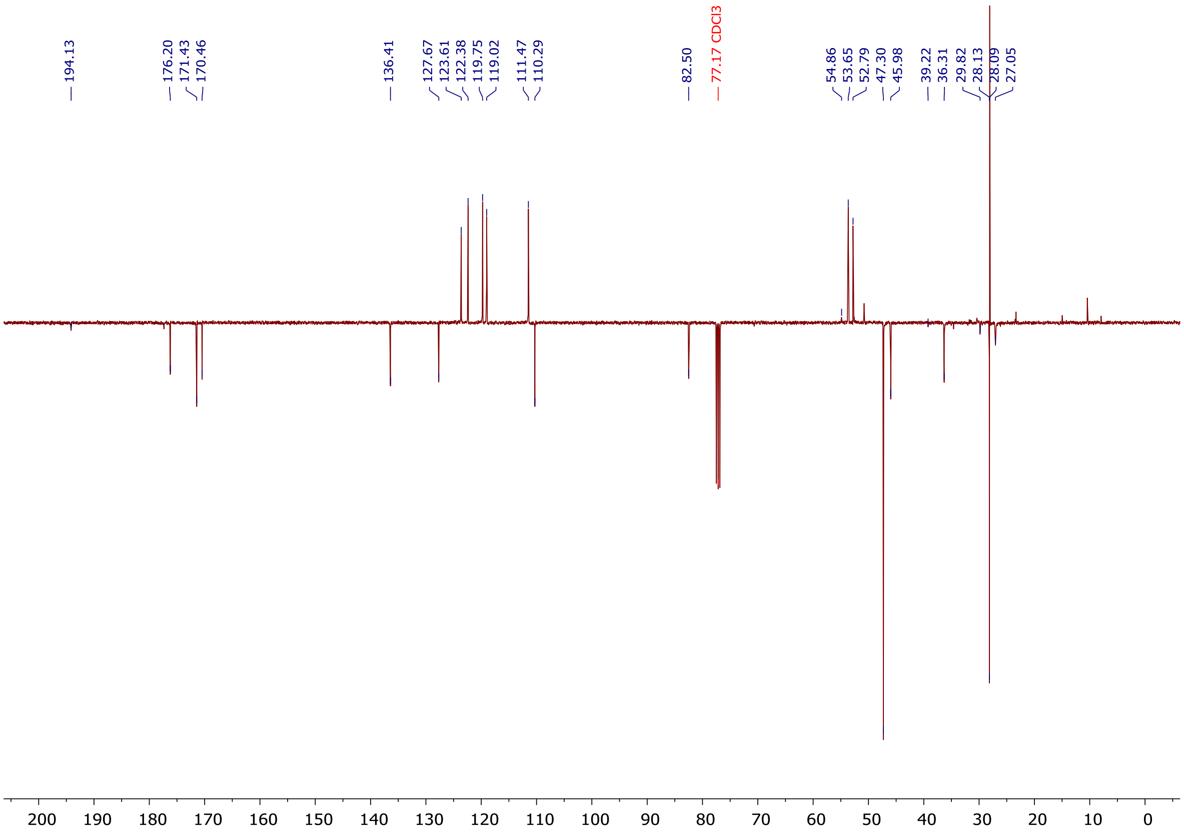

^{1}H and ^{13}C NMR Spectra of compound **P9**.

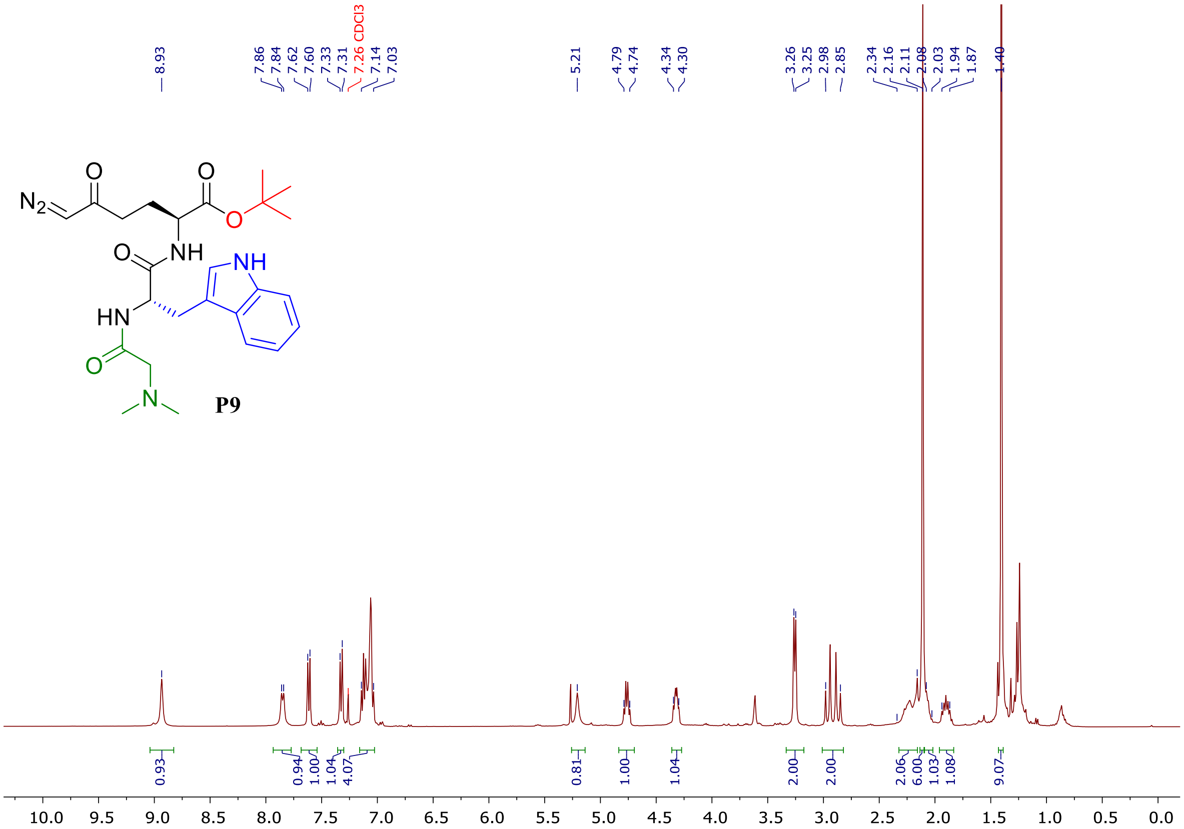

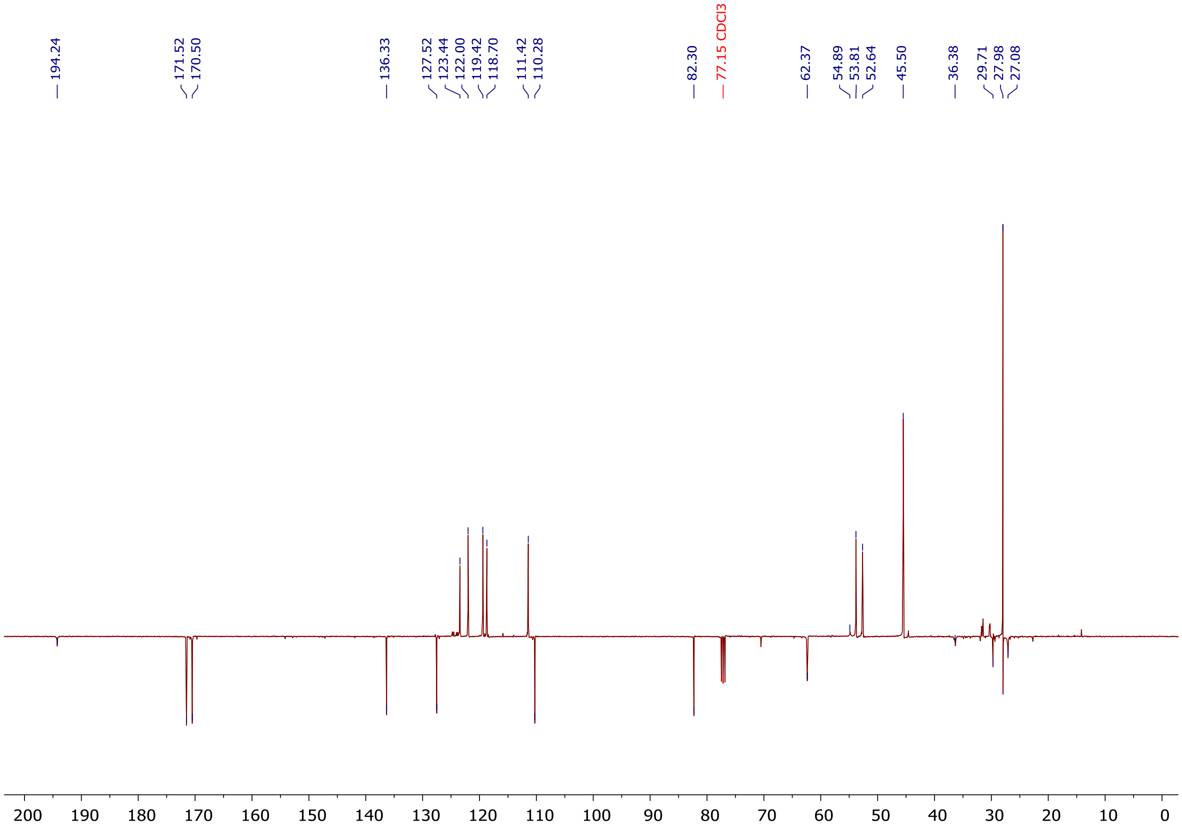

^{1}H and ^{13}C NMR Spectra of compound **P10**.

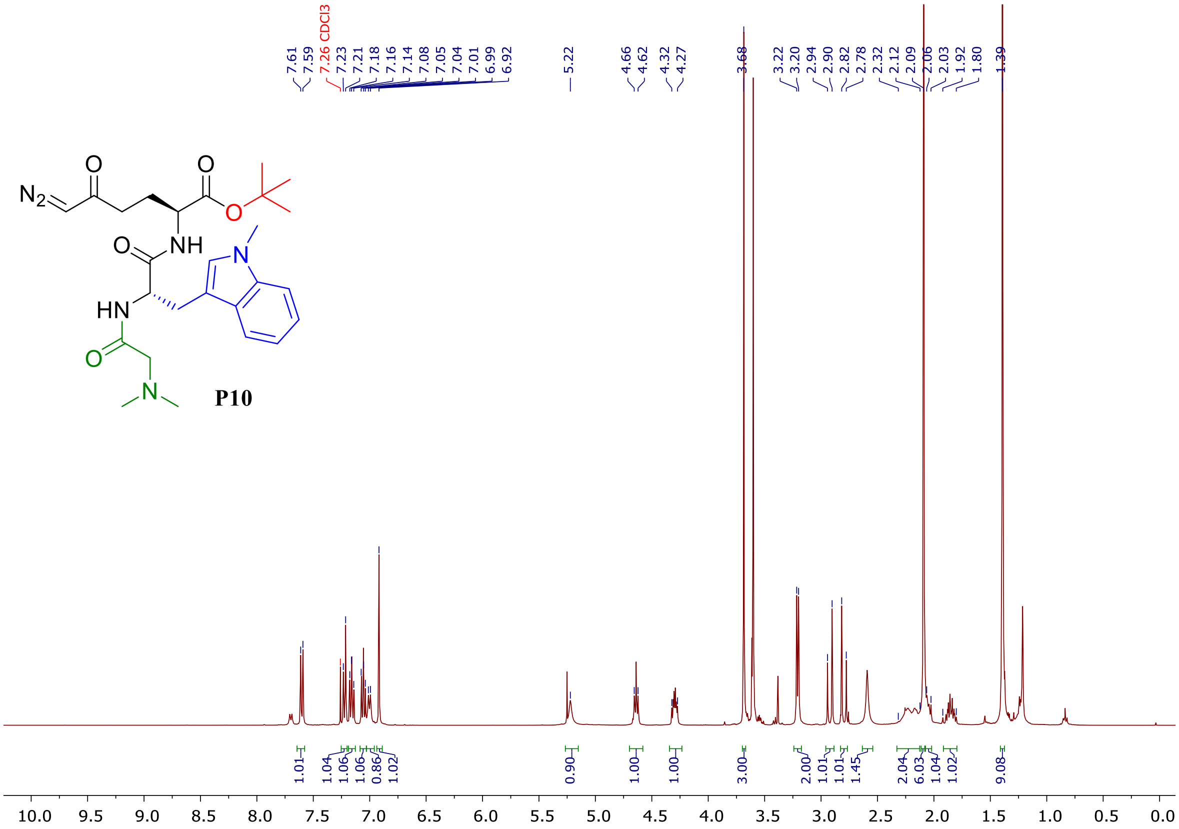

P10

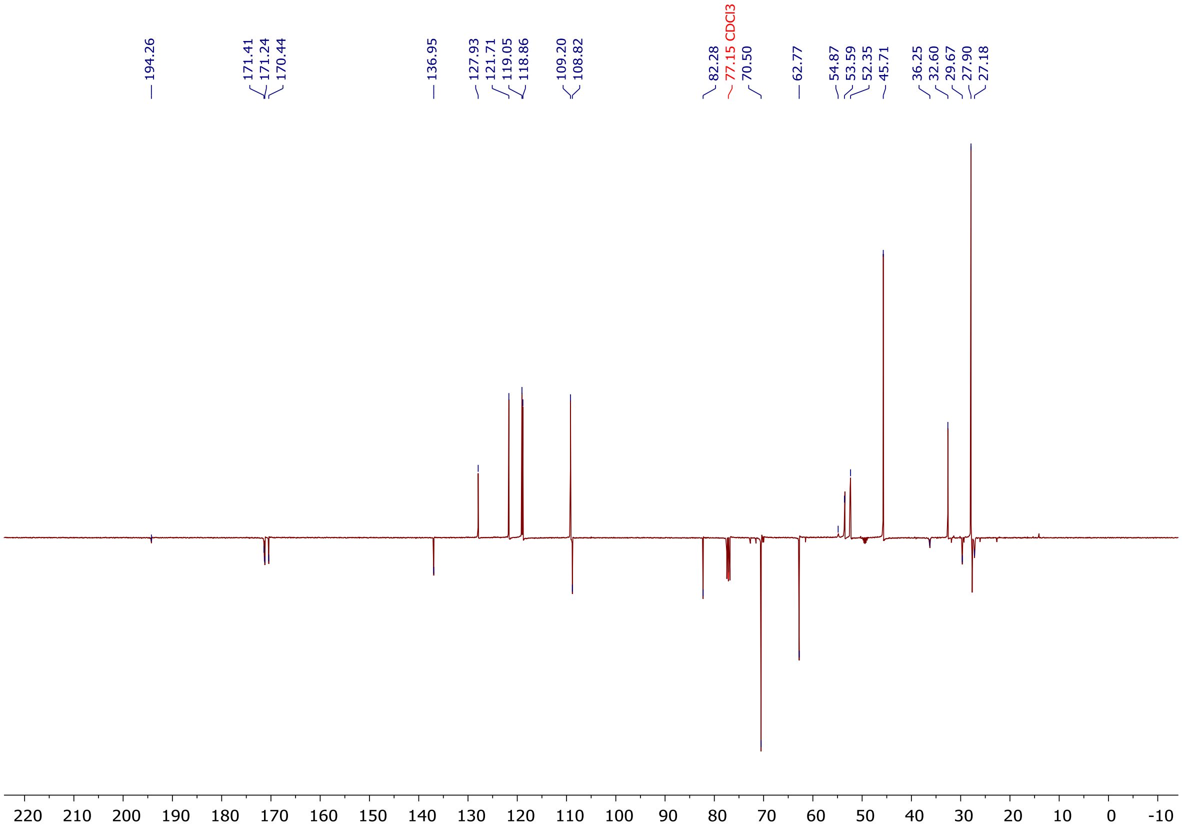

^1H and ^{13}C NMR Spectra of compound **P11**.

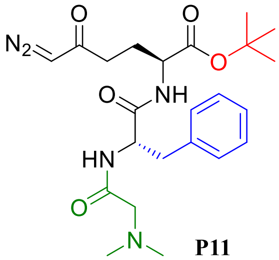

P11

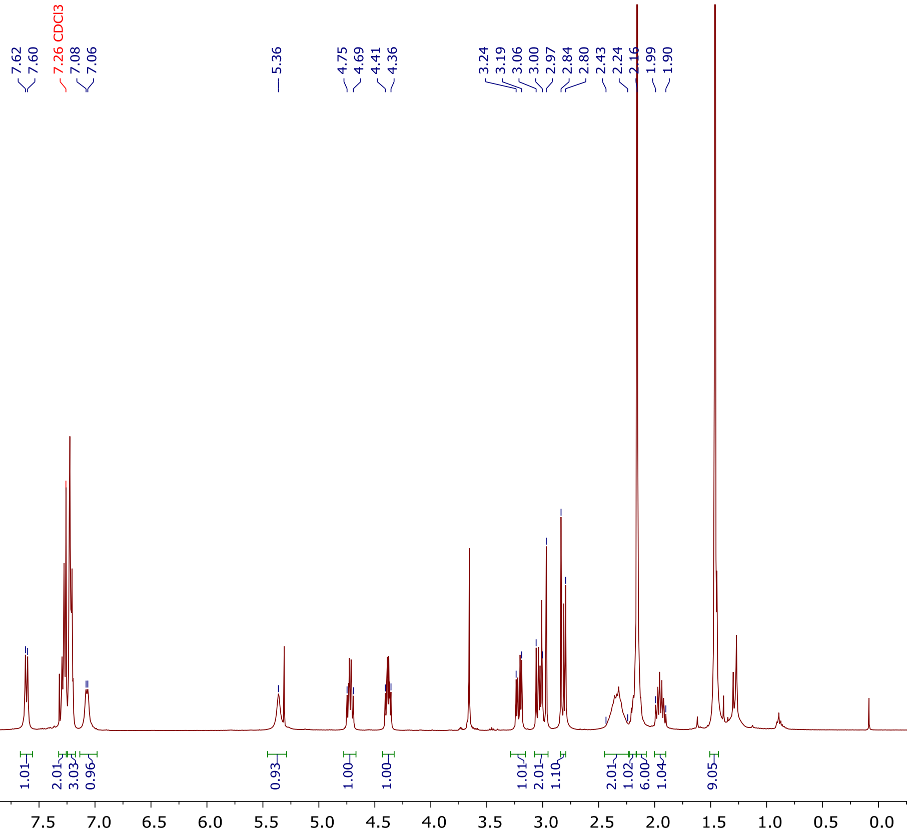

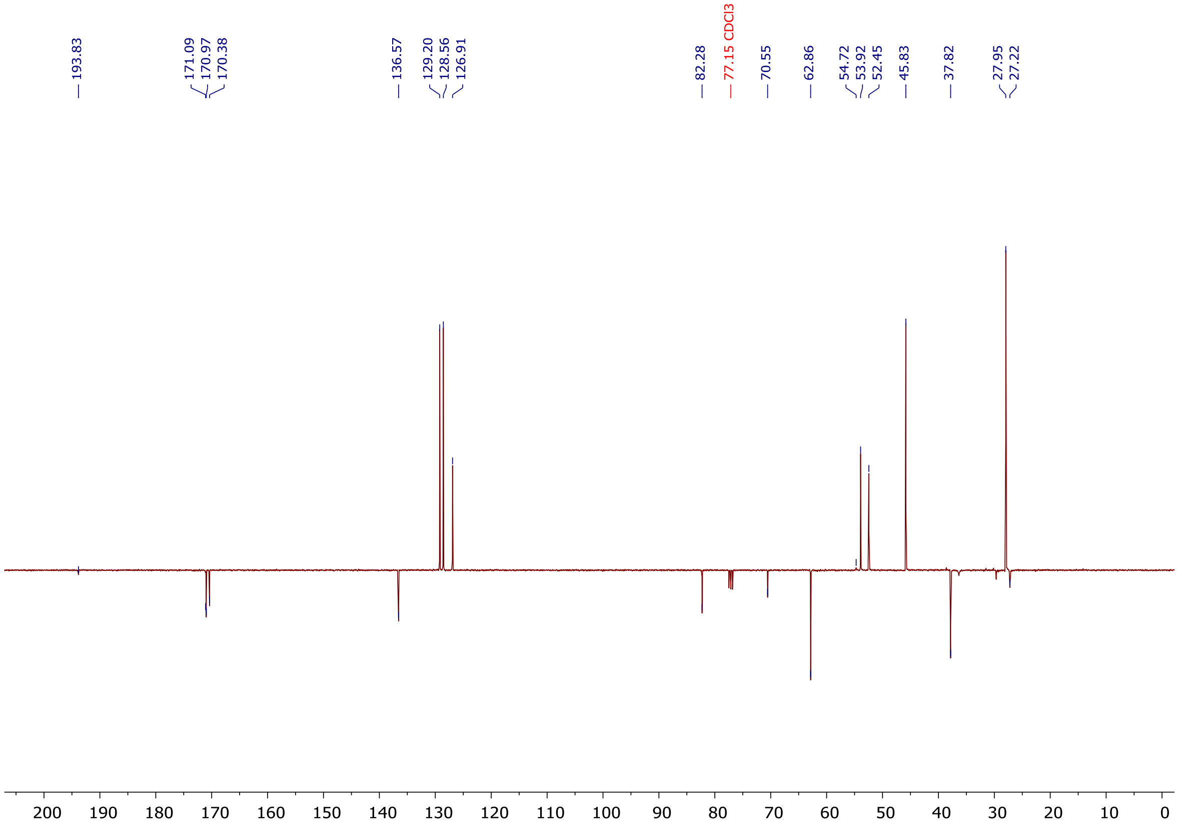

^{1}H and ^{13}C NMR Spectra of compound **P12**.

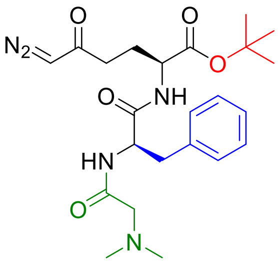

P12

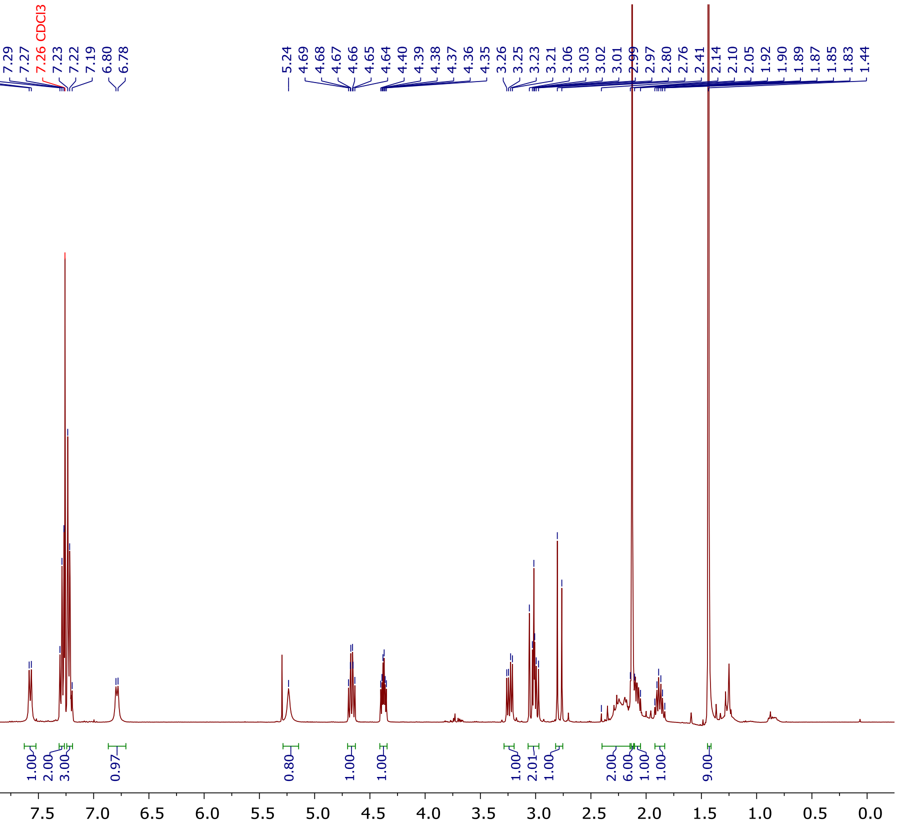

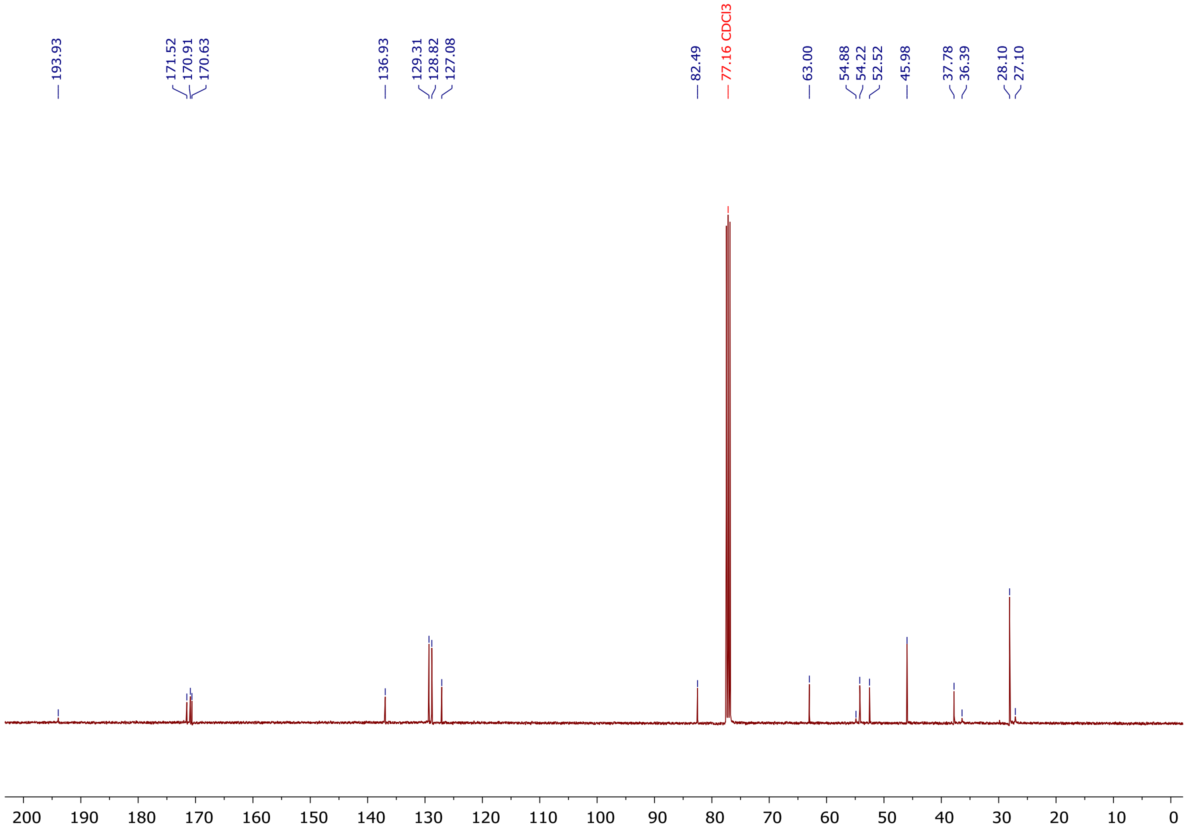

^{1}H and ^{13}C NMR Spectra of compound **P13**.

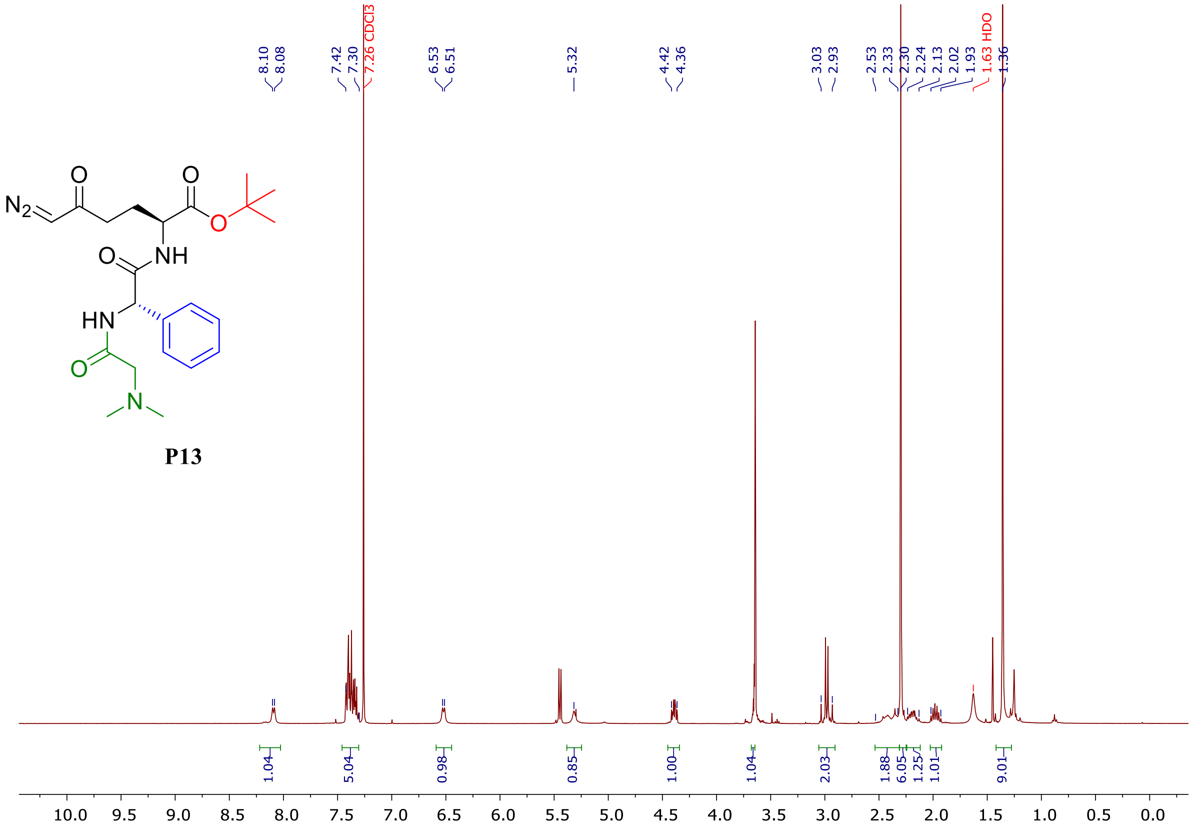

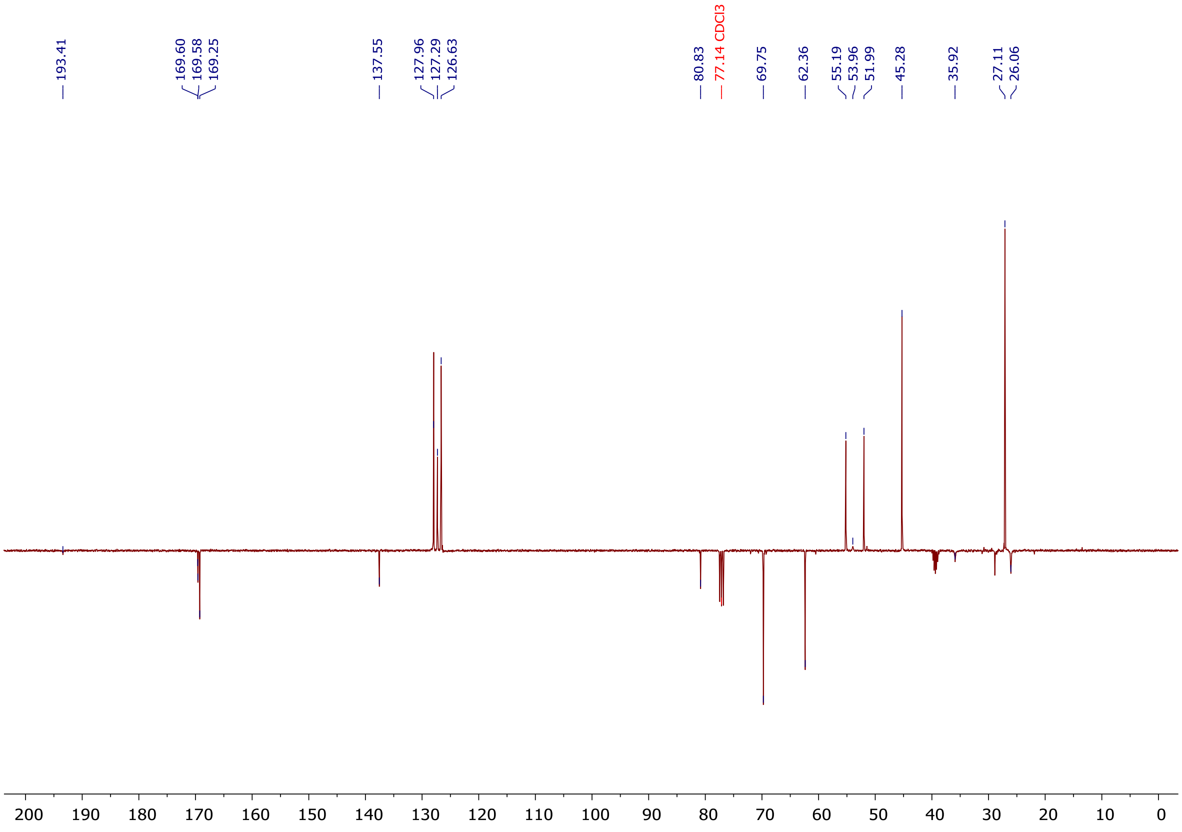

^{1}H and ^{13}C NMR Spectra of compound **P14**.

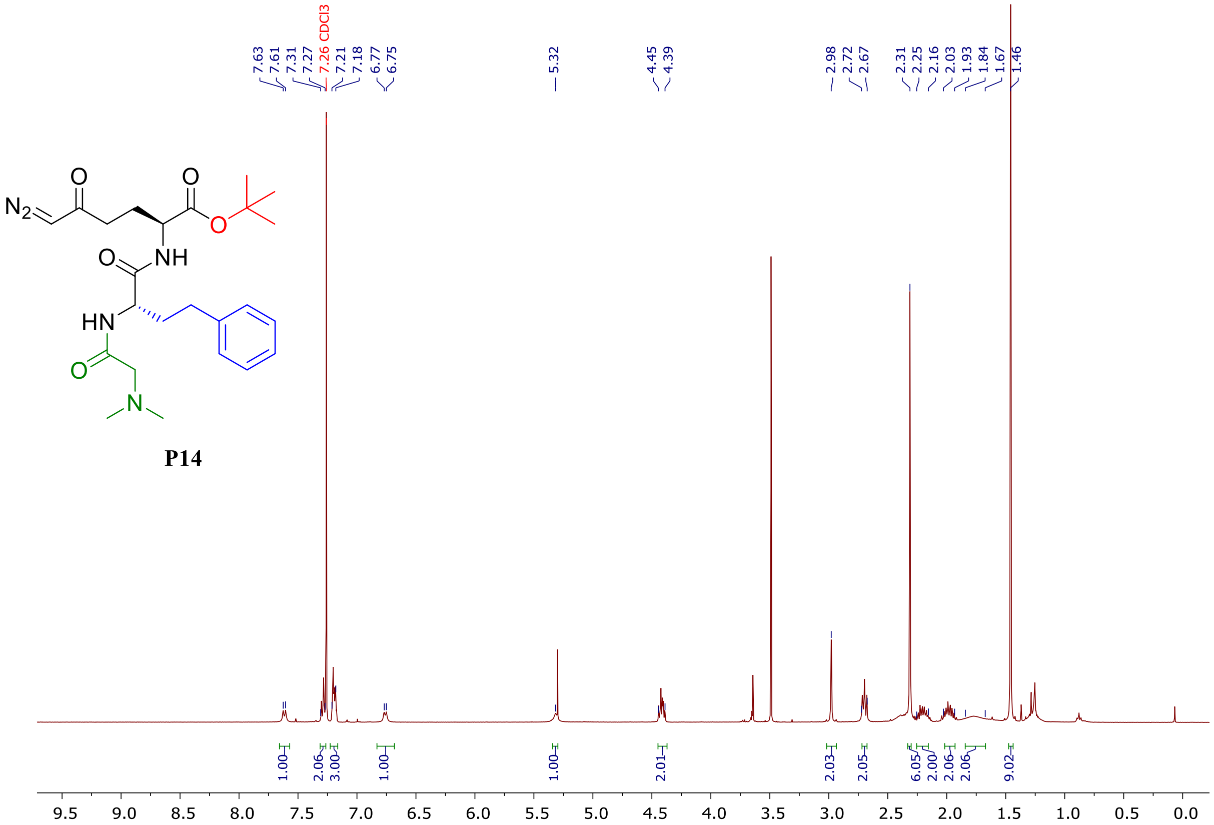

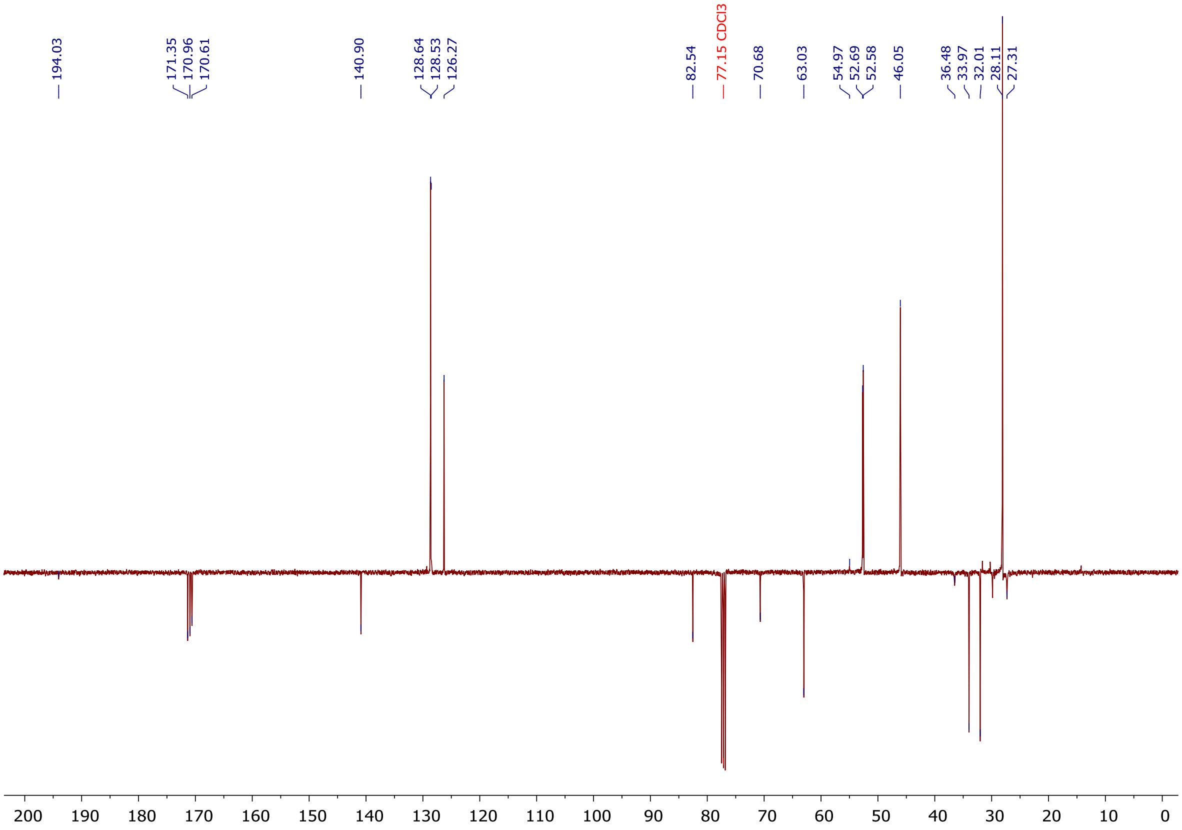

^{1}H and ^{13}C NMR Spectra of compound **P15**.

P15

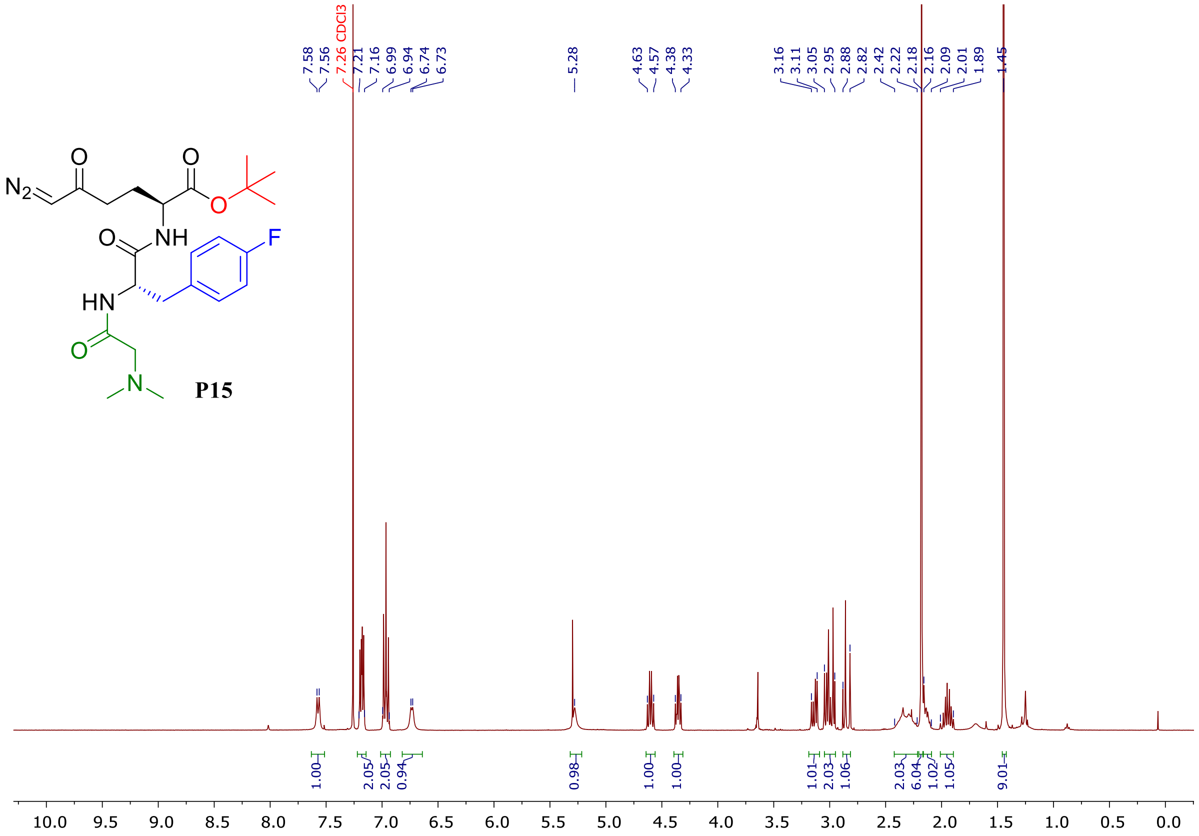

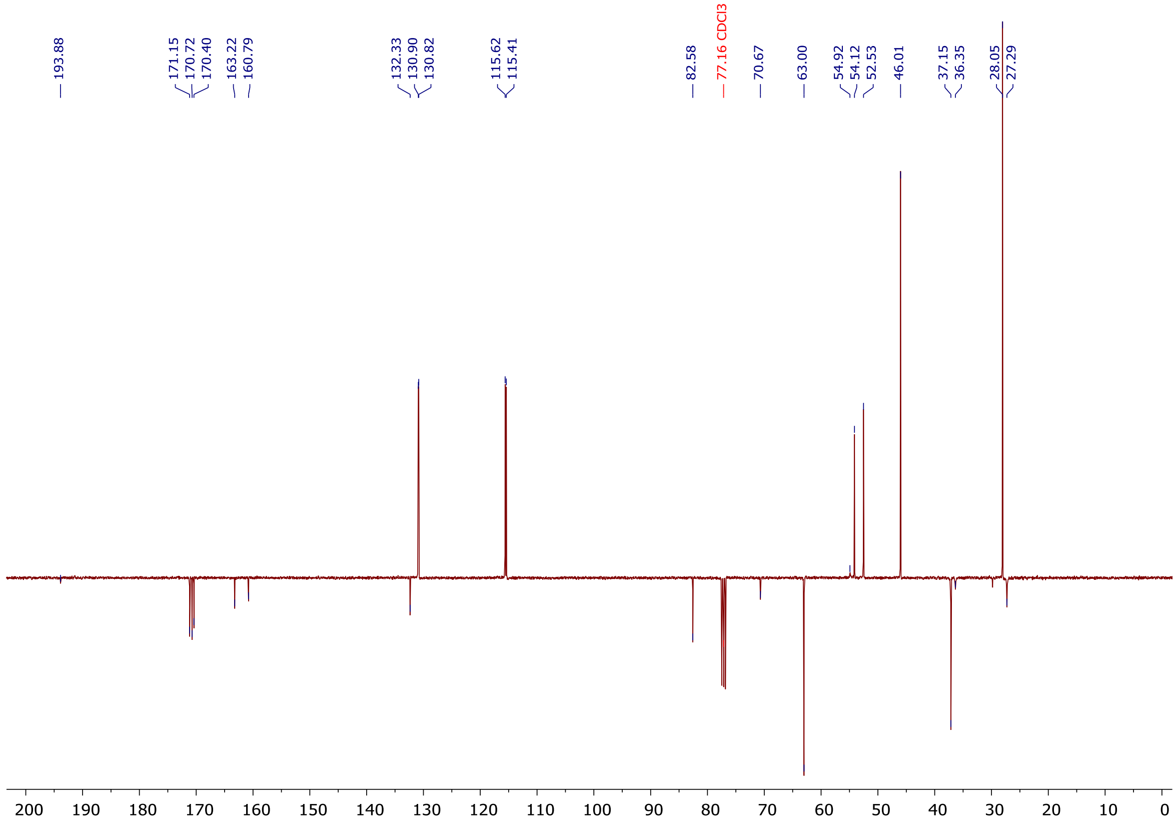

^{1}H and ^{13}C NMR Spectra of compound **P16**.

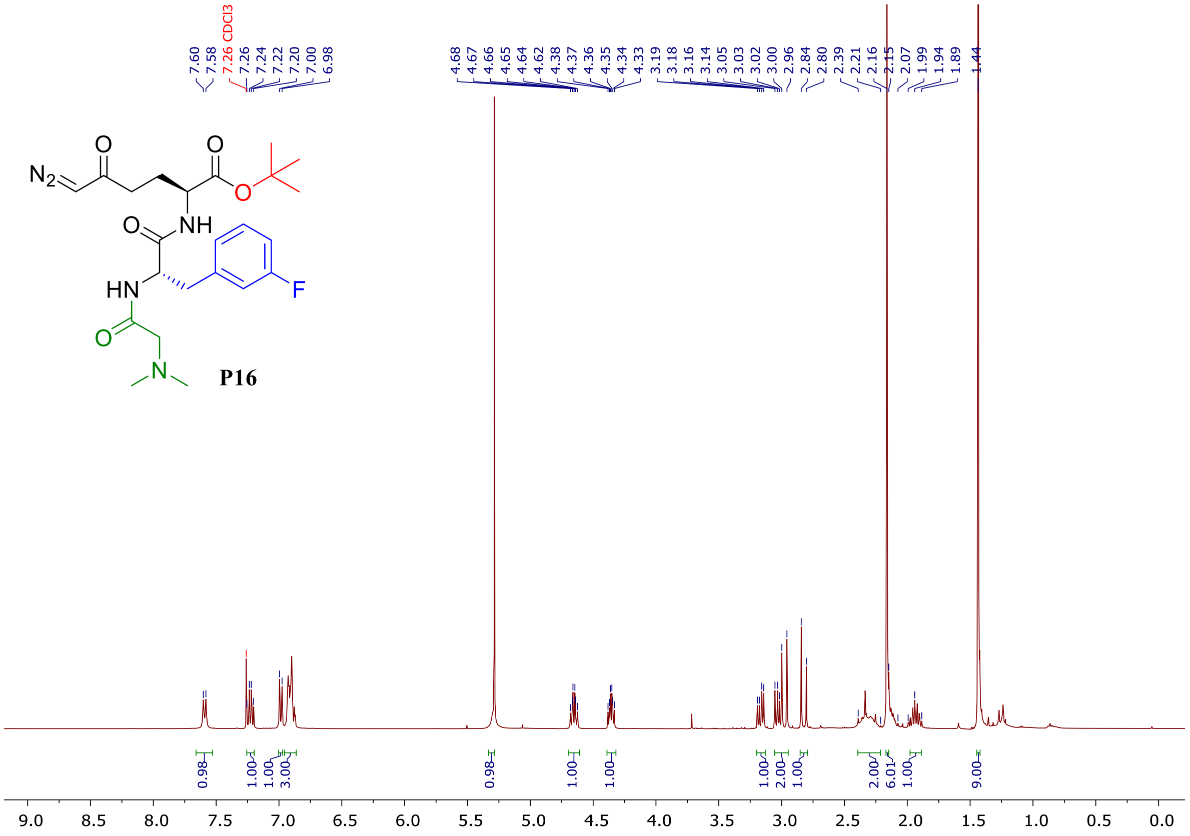

P16

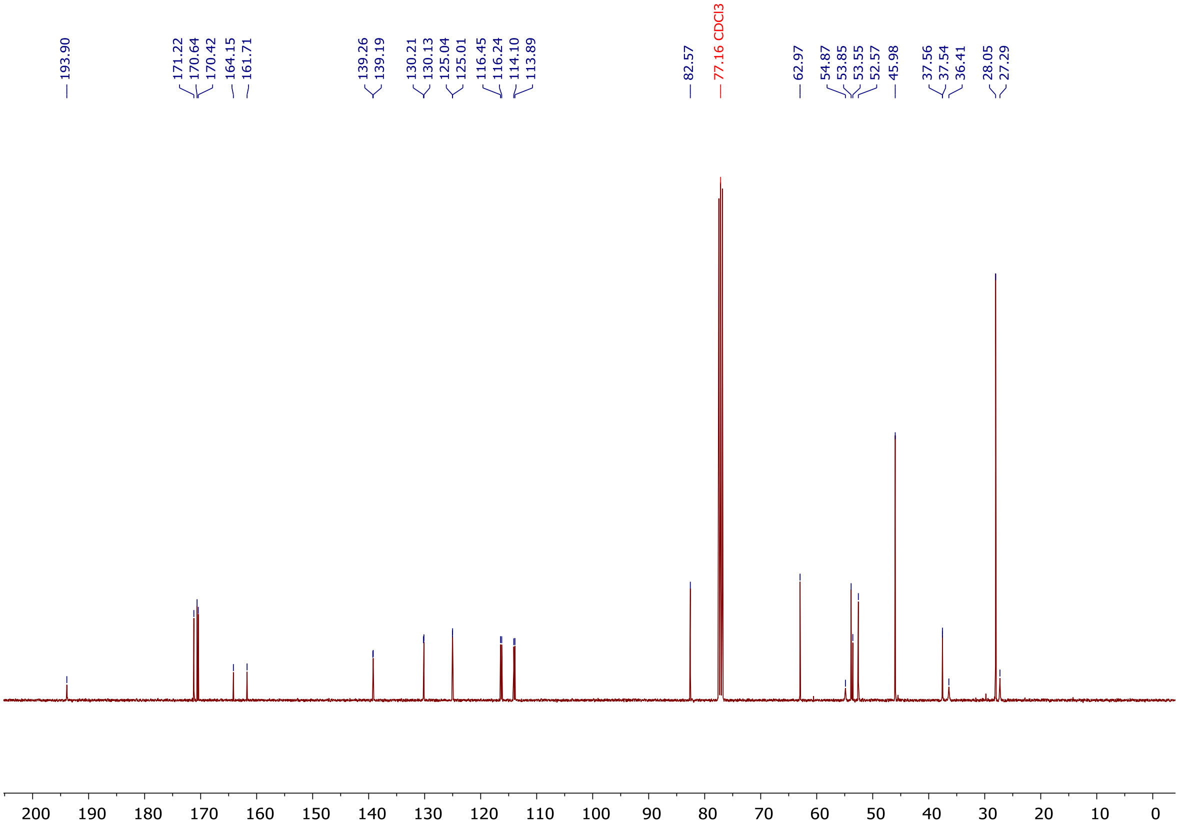

^{1}H and ^{13}C NMR Spectra of compound **P17**.

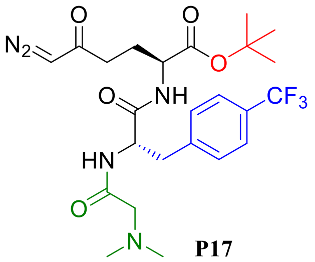

P17

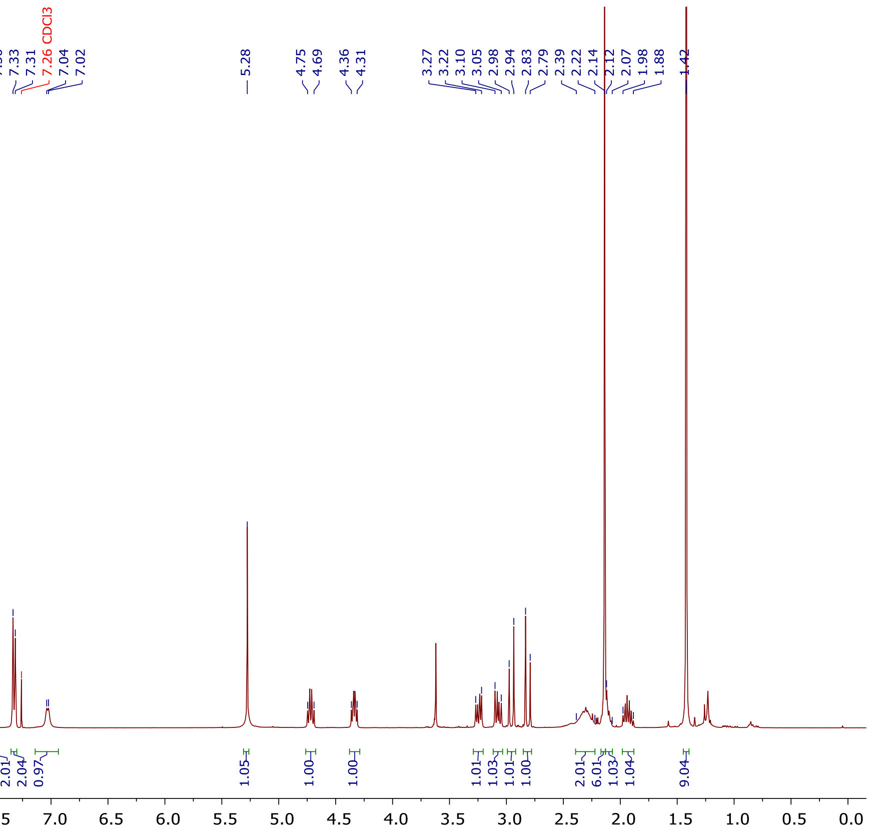

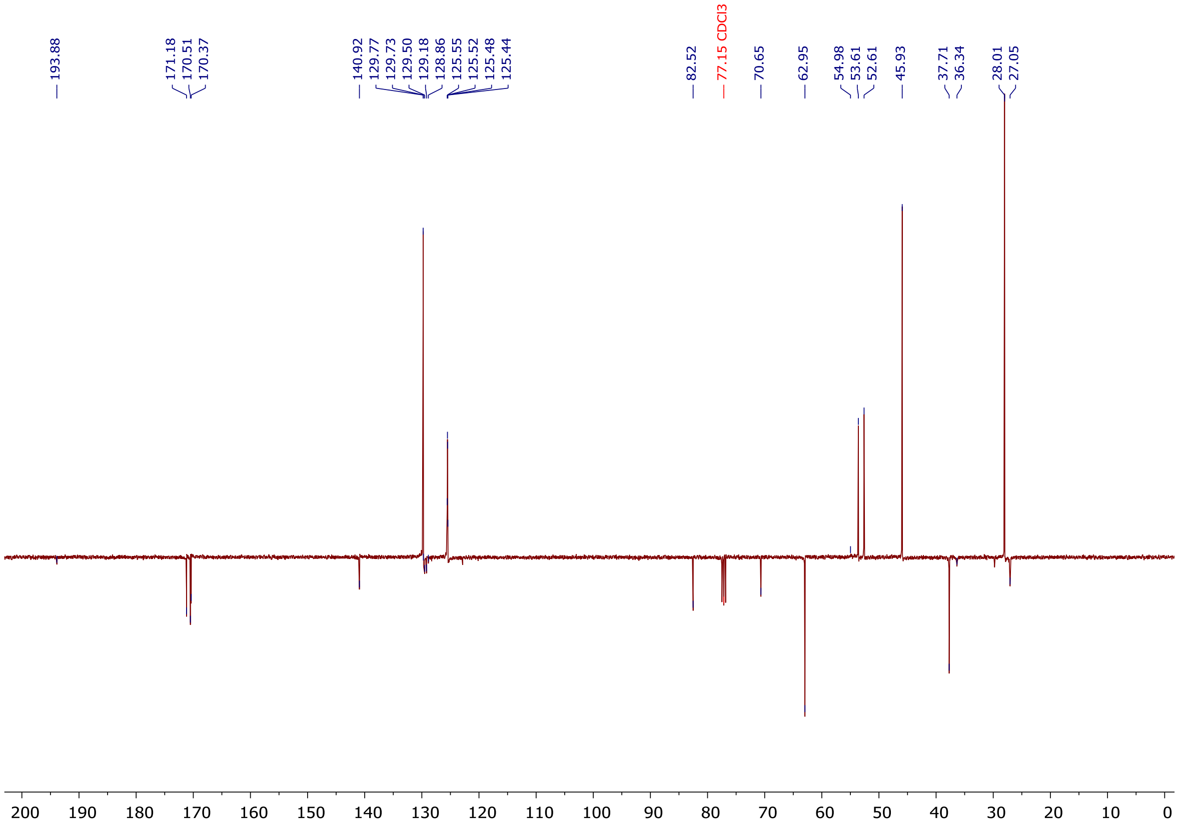

^{1}H and ^{13}C NMR Spectra of compound **P18**.

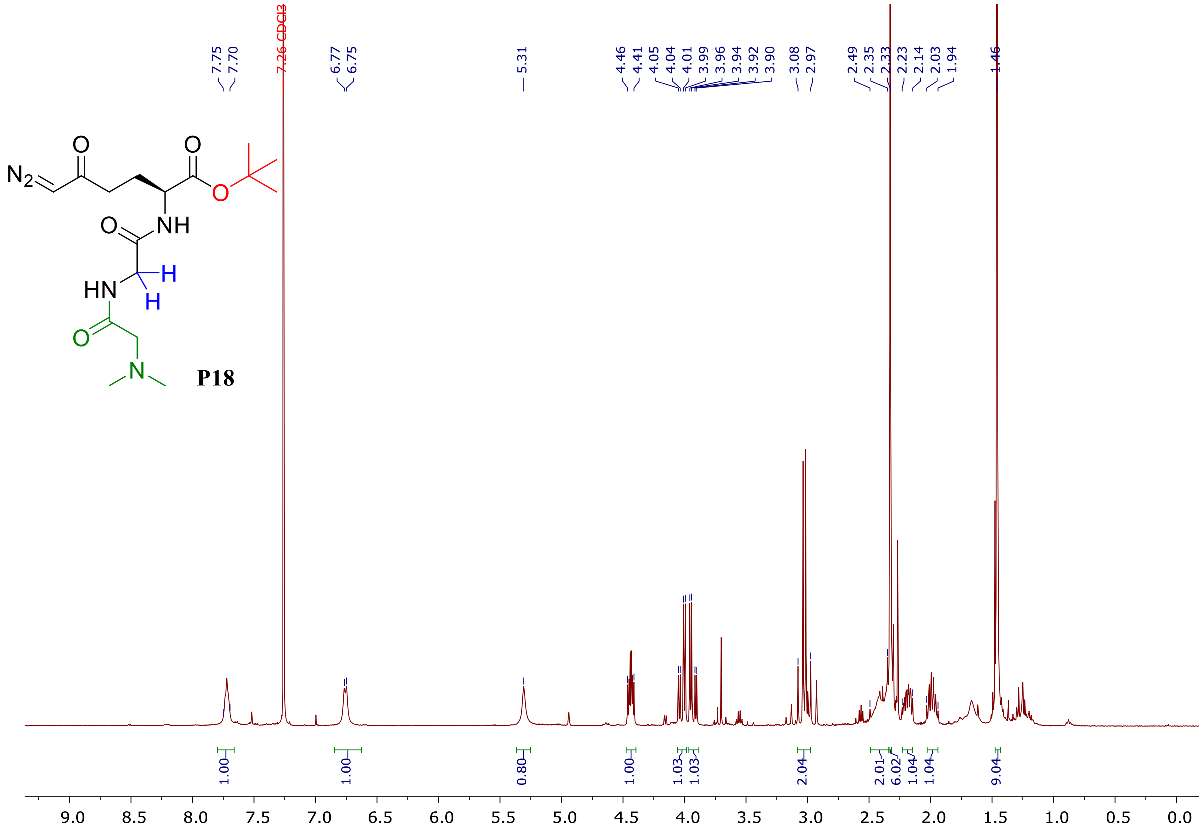

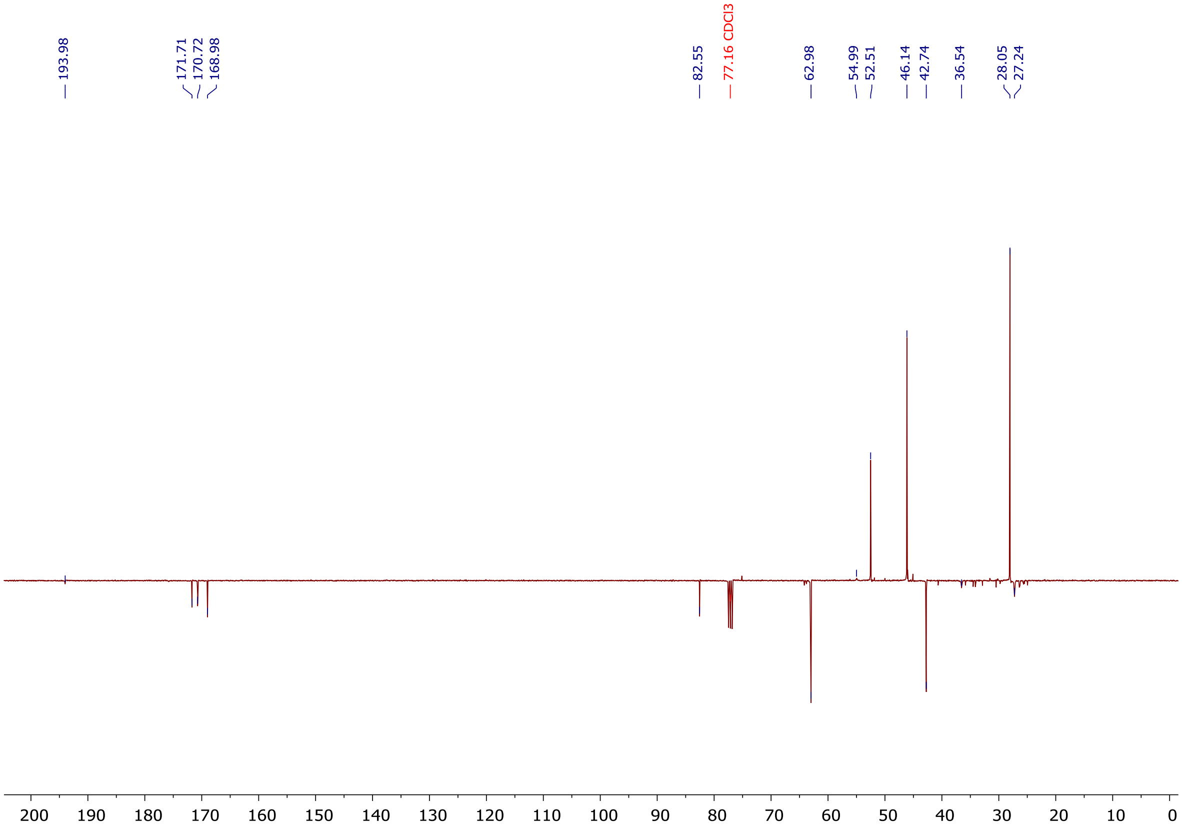

^{1}H and ^{13}C NMR Spectra of compound **P19**.

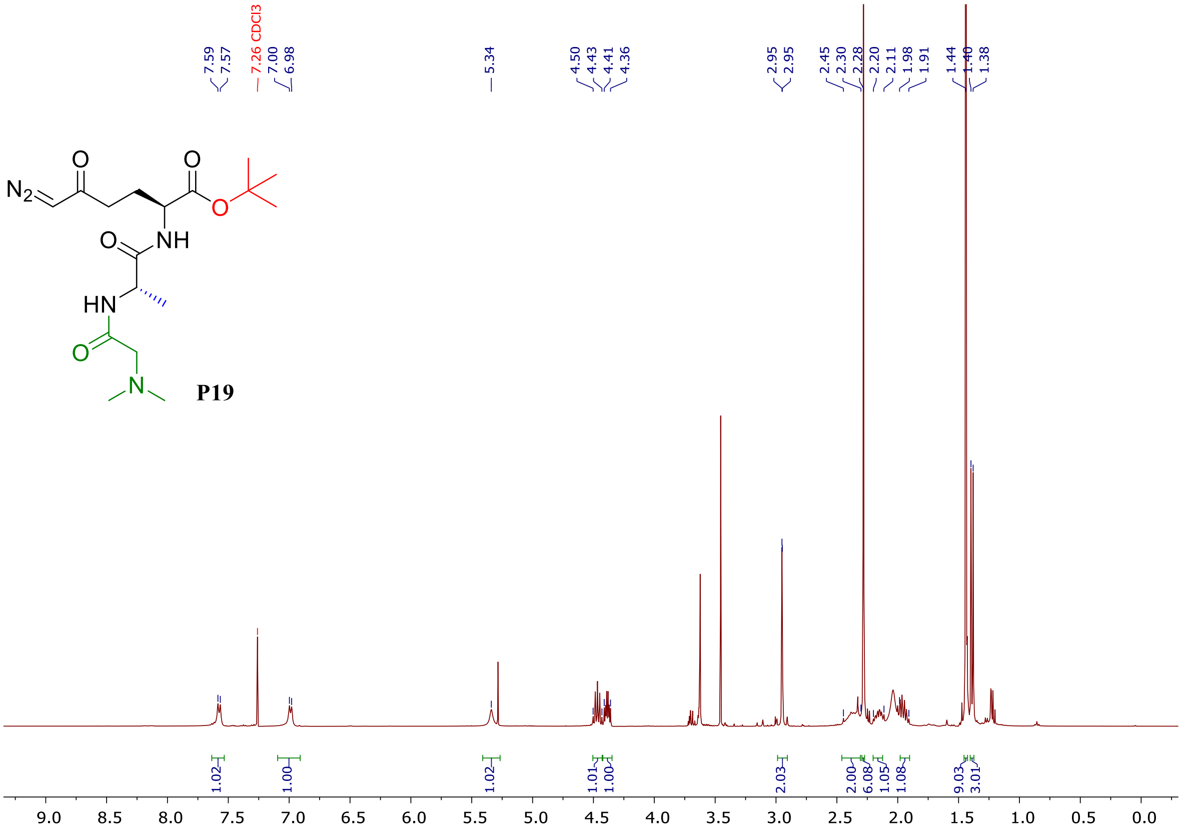

P19

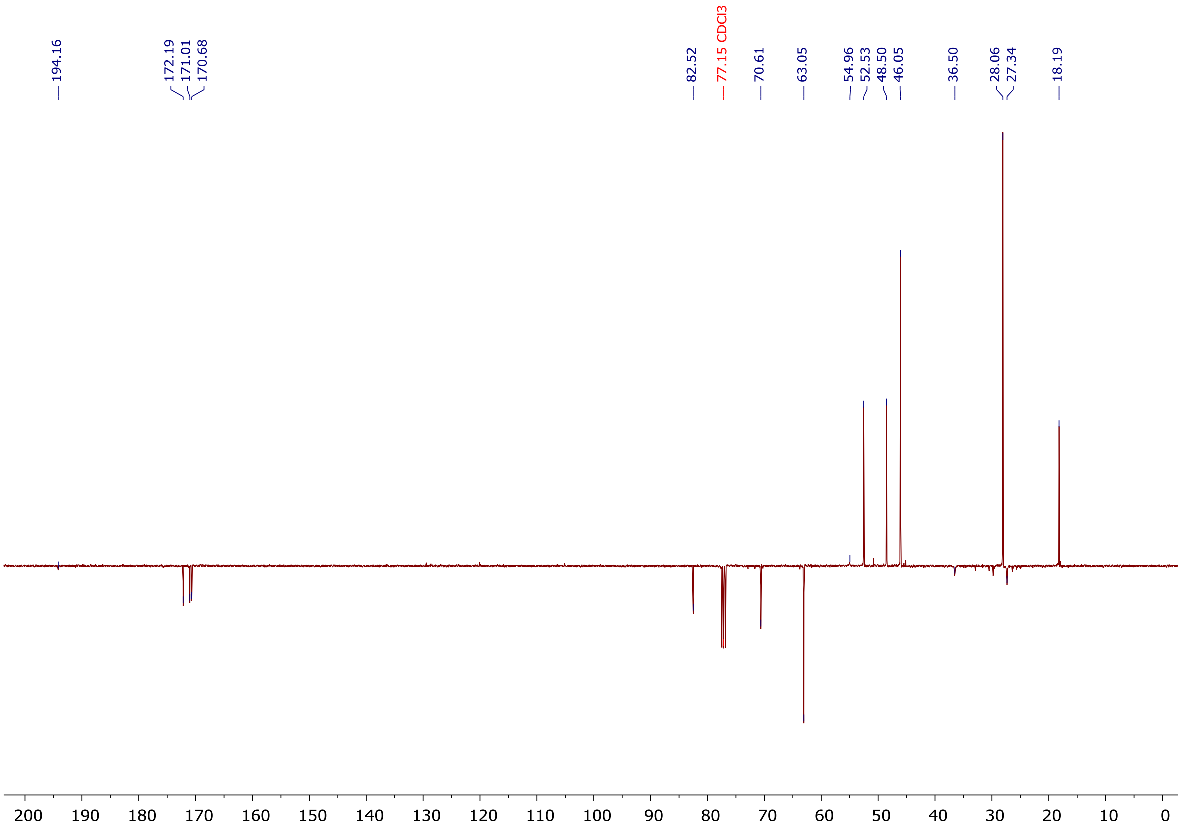

^{1}H and ^{13}C NMR Spectra of compound **P20**.

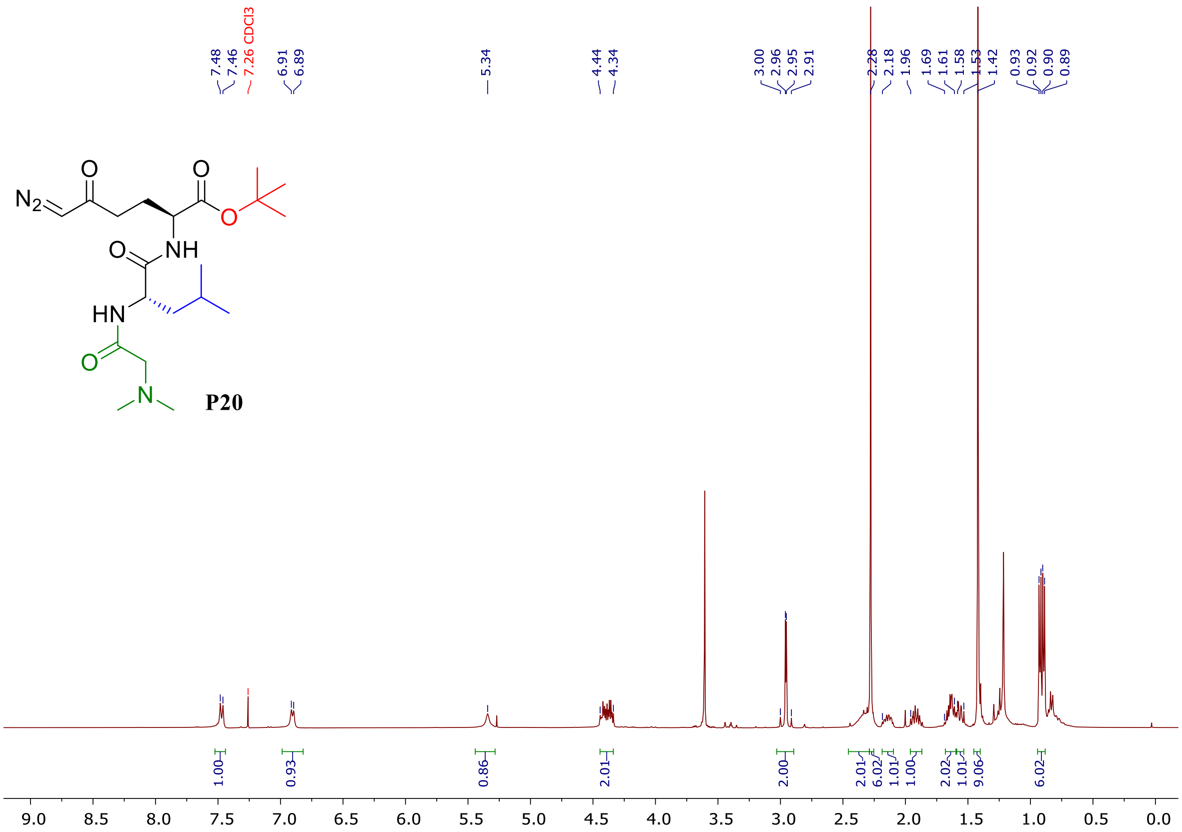

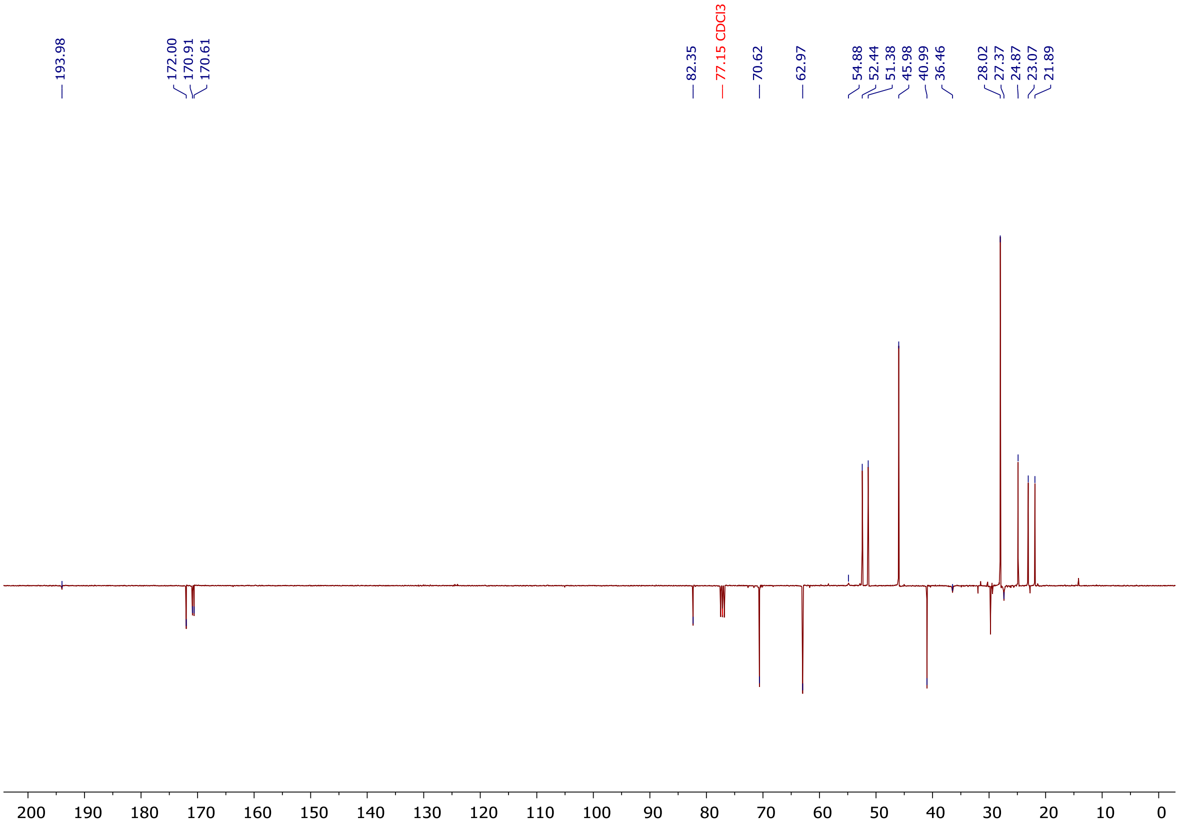

^{1}H and ^{13}C NMR Spectra of compound **P21**.

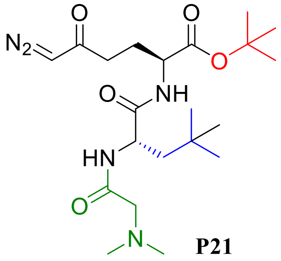

P21

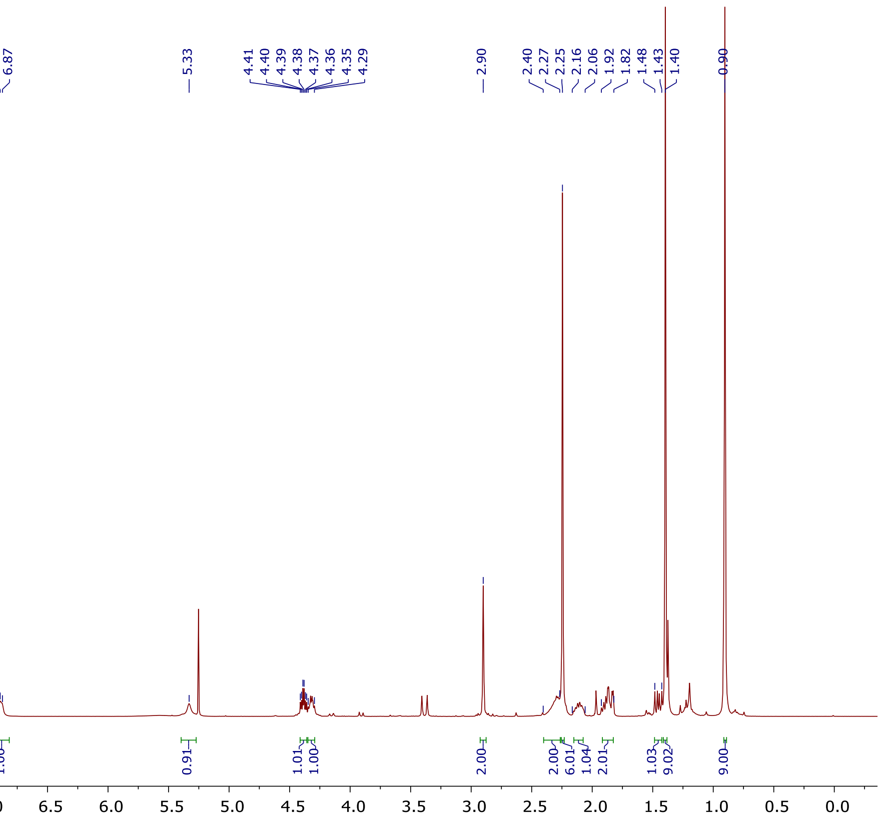

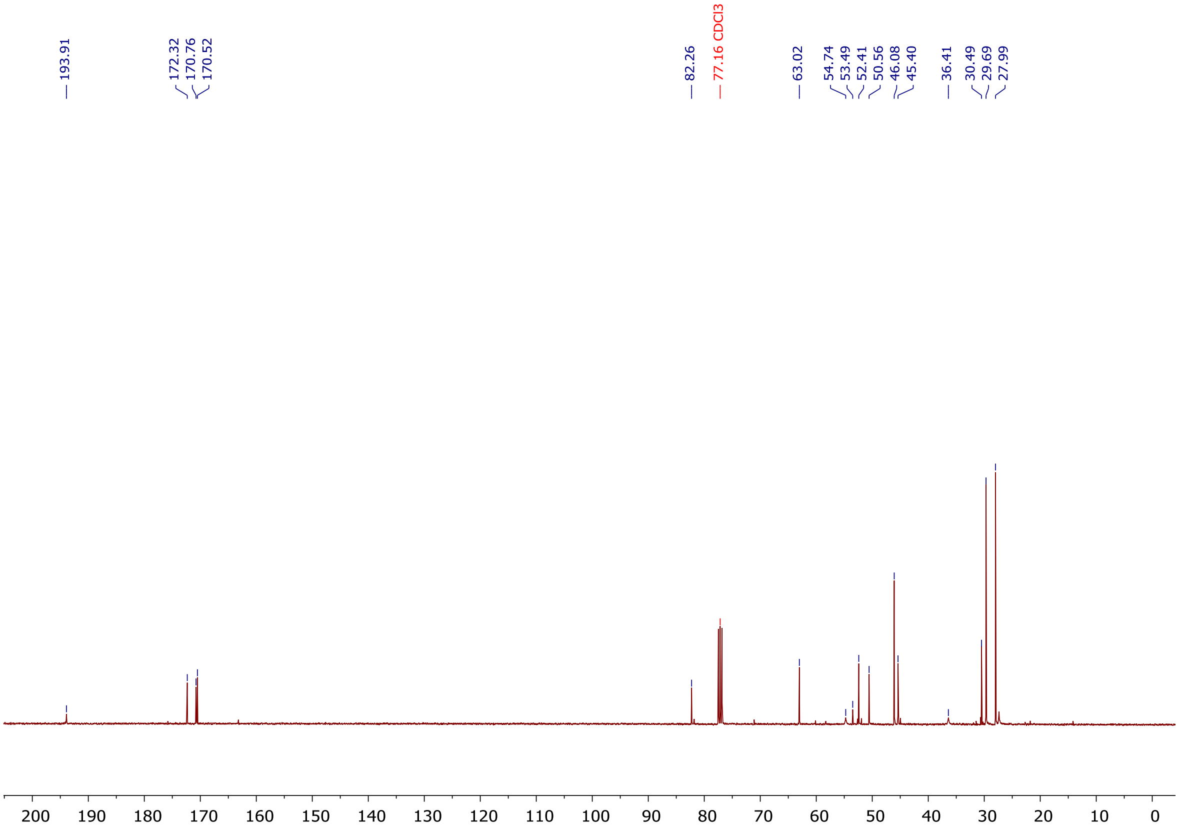

HPLC spectra of final prodrugs P1-P21

HPLC spectrum of prodrug **P1**

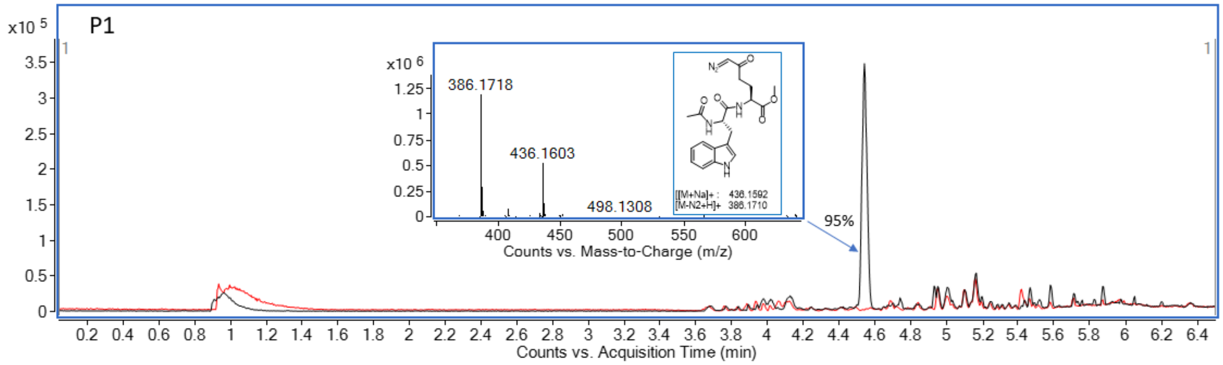

HPLC spectrum of prodrug **P2**

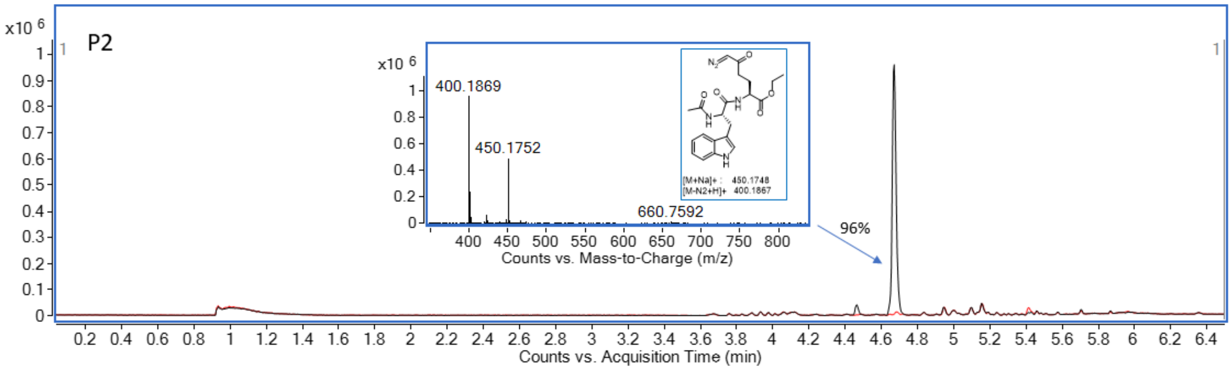

HPLC spectrum of prodrug **P3**

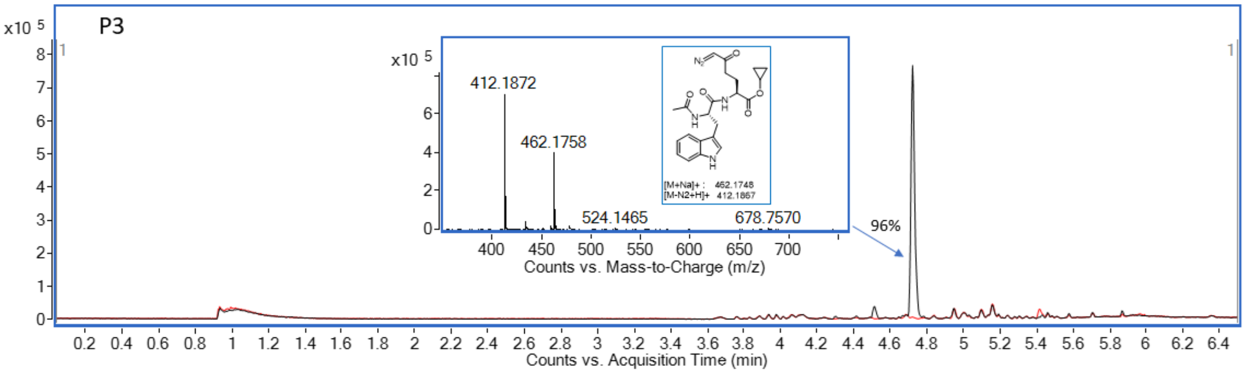

HPLC spectrum of prodrug **P4**

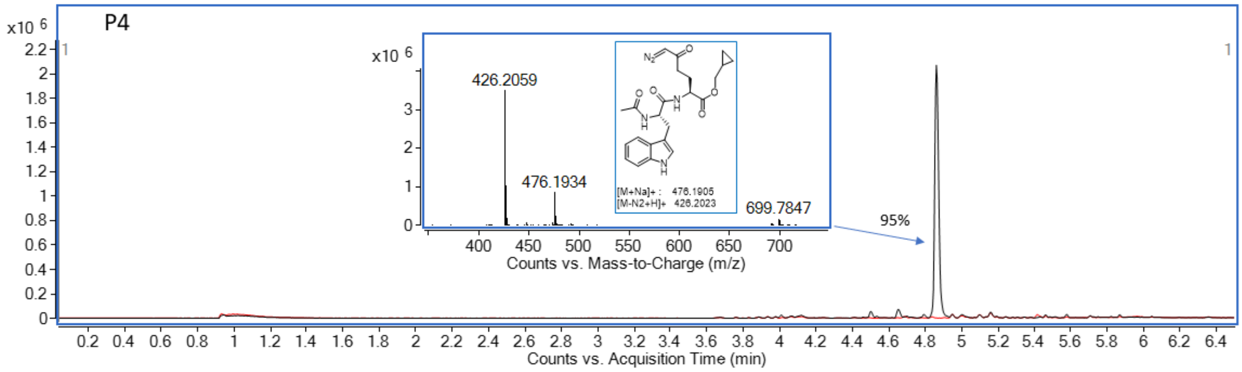

HPLC spectrum of prodrug **P5**

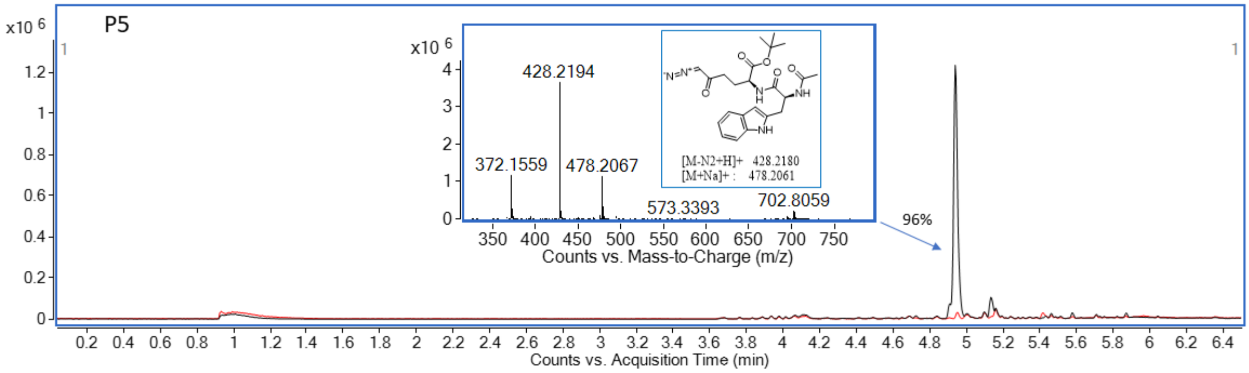

HPLC spectrum of prodrug **P6**

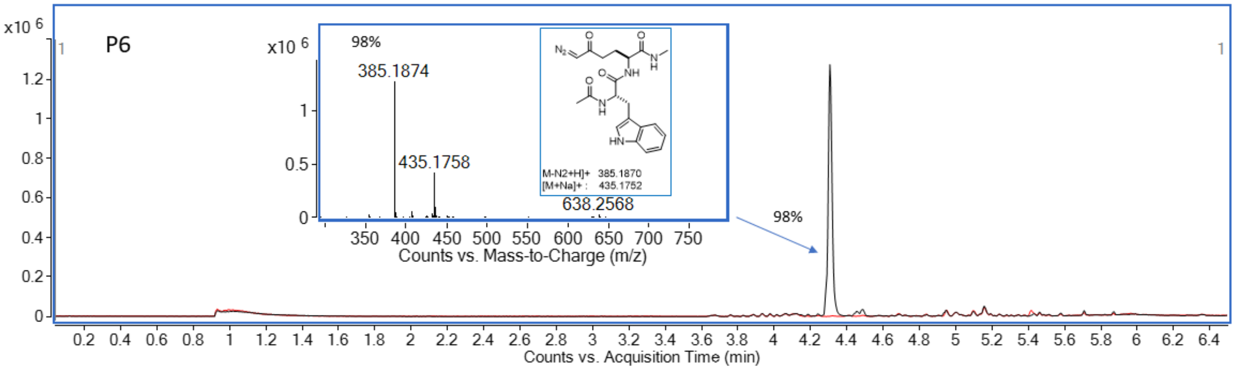

HPLC spectrum of prodrug **P7**

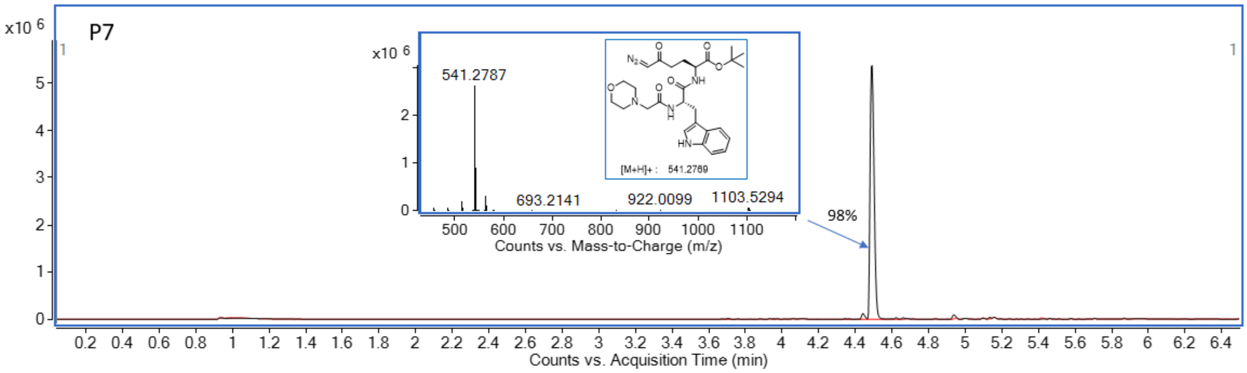

HPLC spectrum of prodrug **P8**

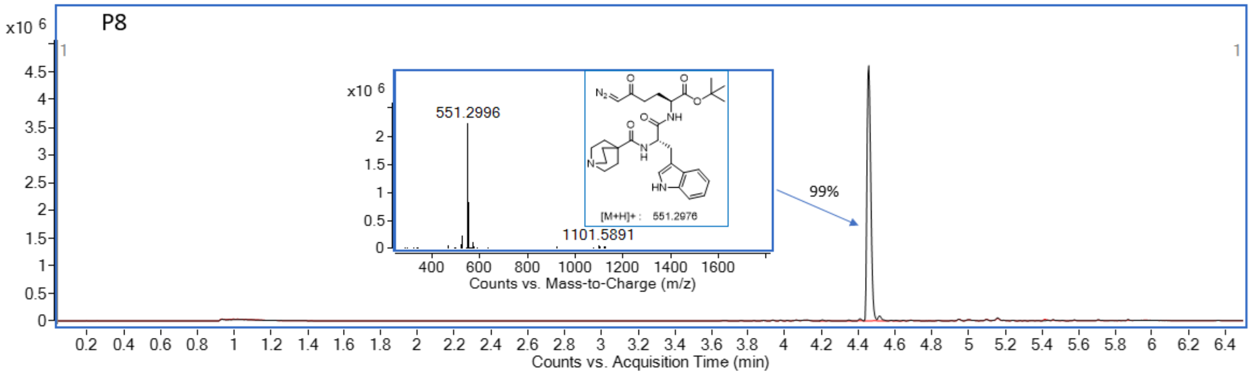

HPLC spectrum of prodrug **P9**

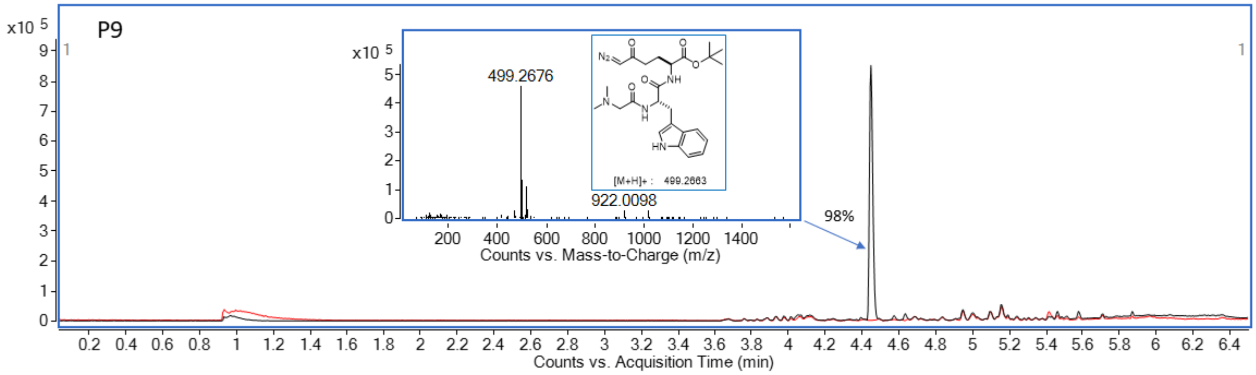

HPLC spectrum of prodrug **P10**

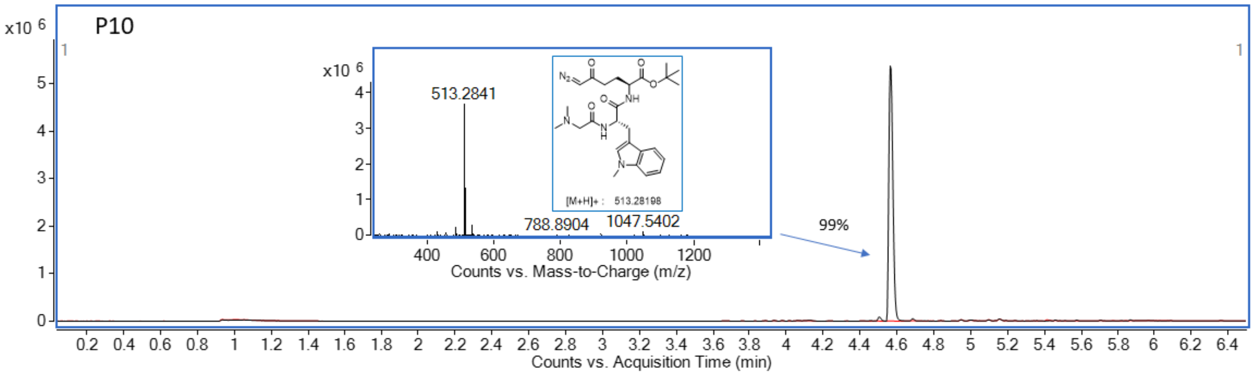

HPLC spectrum of prodrug **P11**

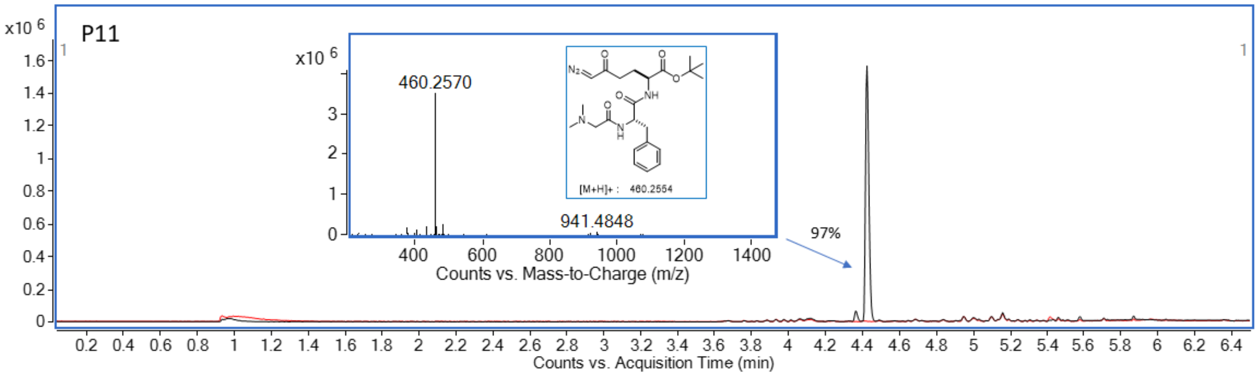

HPLC spectrum of prodrug **P12**

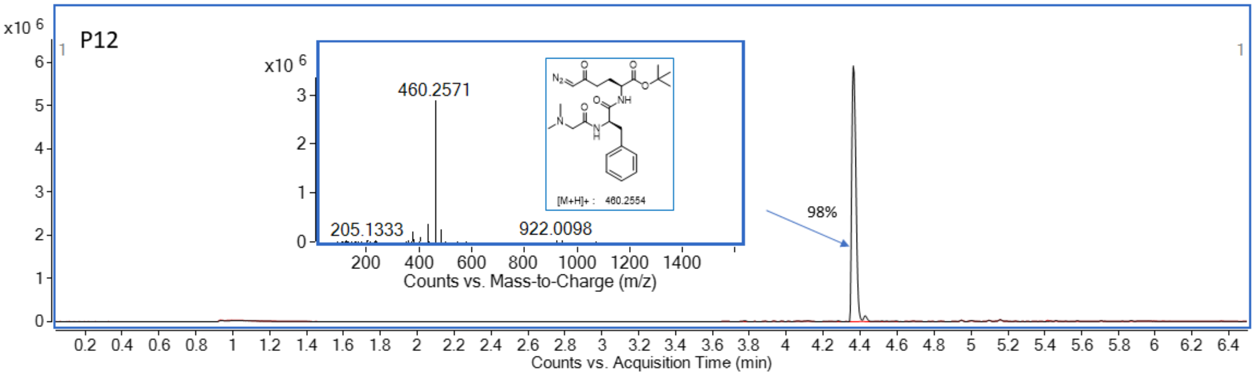

HPLC spectrum of prodrug **P13**

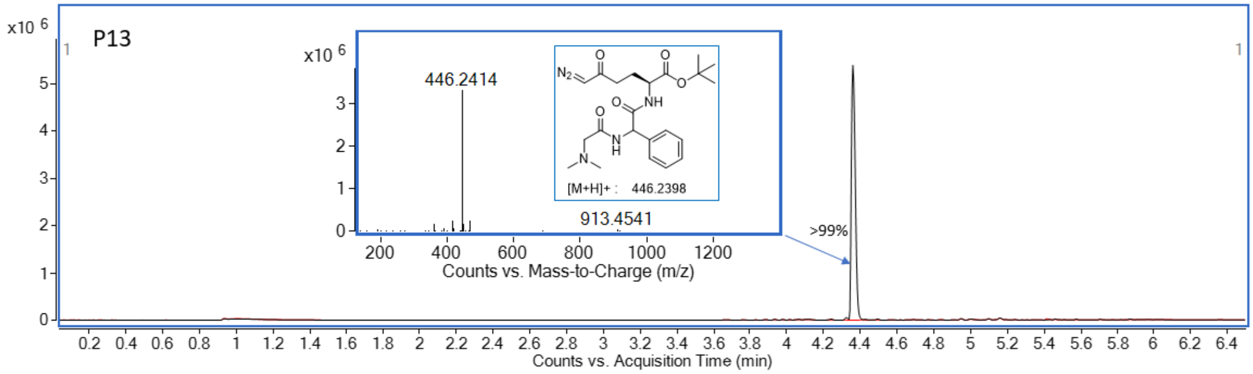

HPLC spectrum of prodrug **P14**

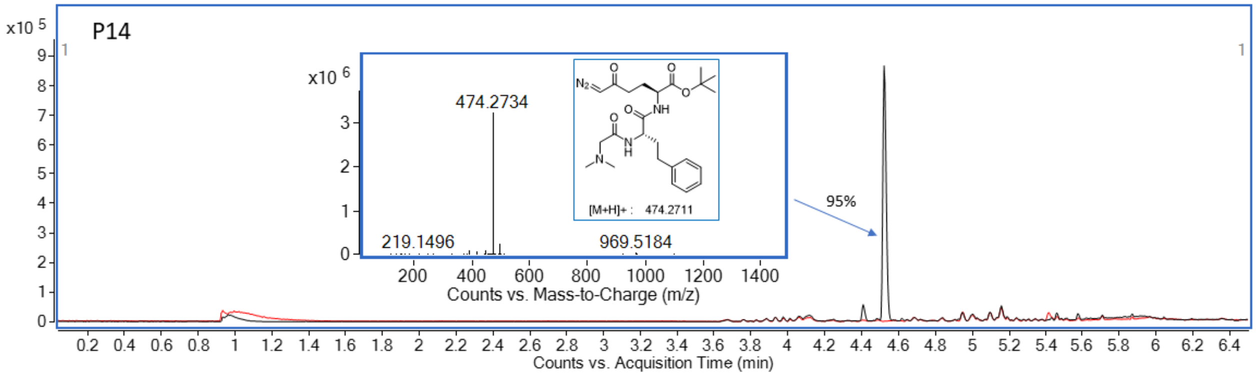

HPLC spectrum of prodrug **P15**

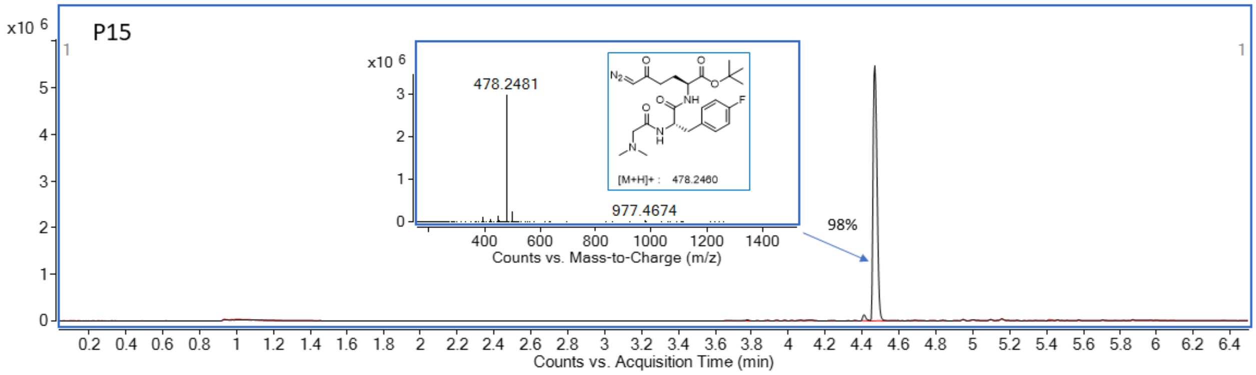

HPLC spectrum of prodrug **P16**

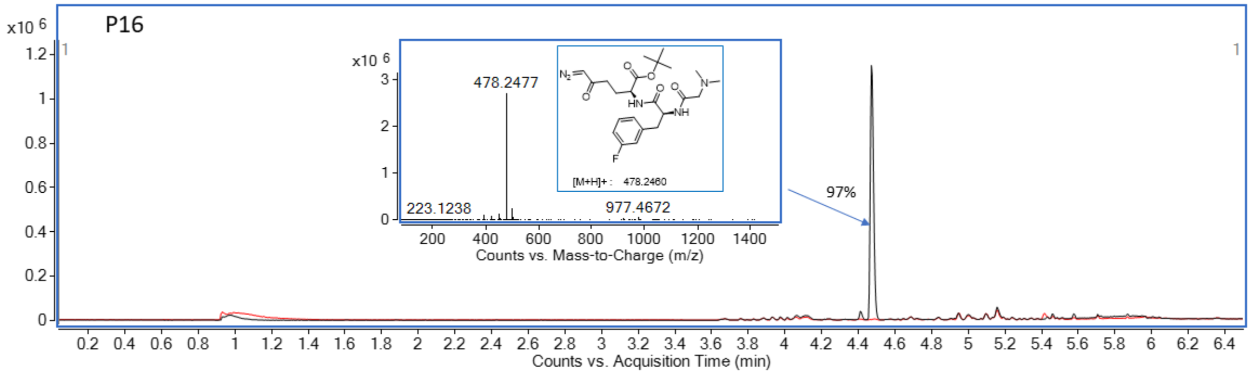

HPLC spectrum of prodrug **P17**

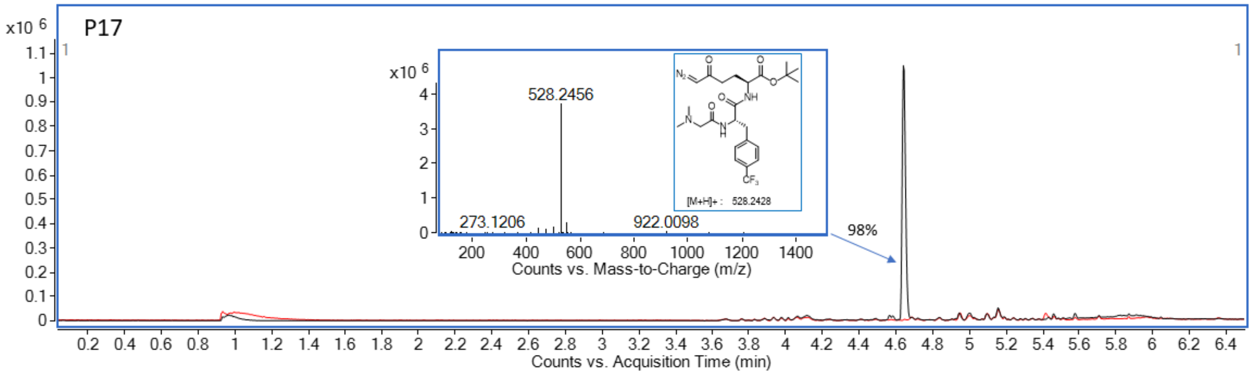

HPLC spectrum of prodrug **P18**

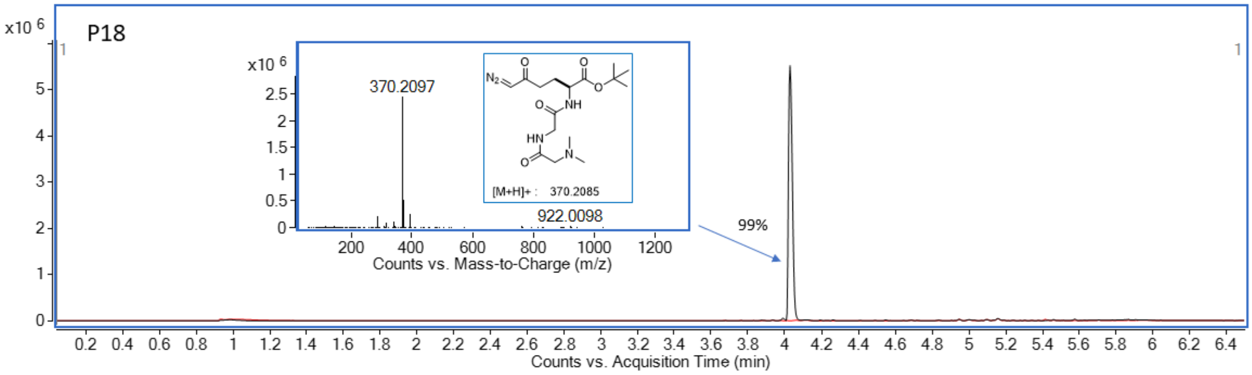

HPLC spectrum of prodrug **P19**

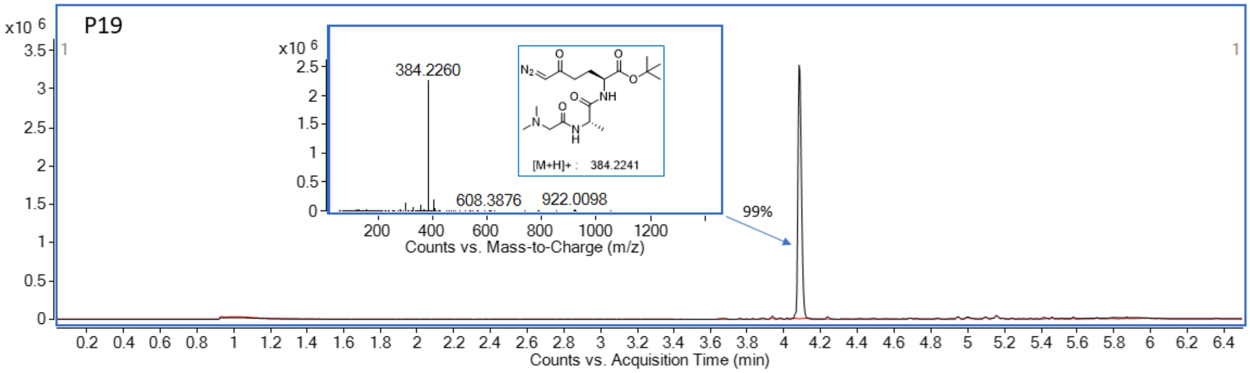

HPLC spectrum of prodrug **P20**

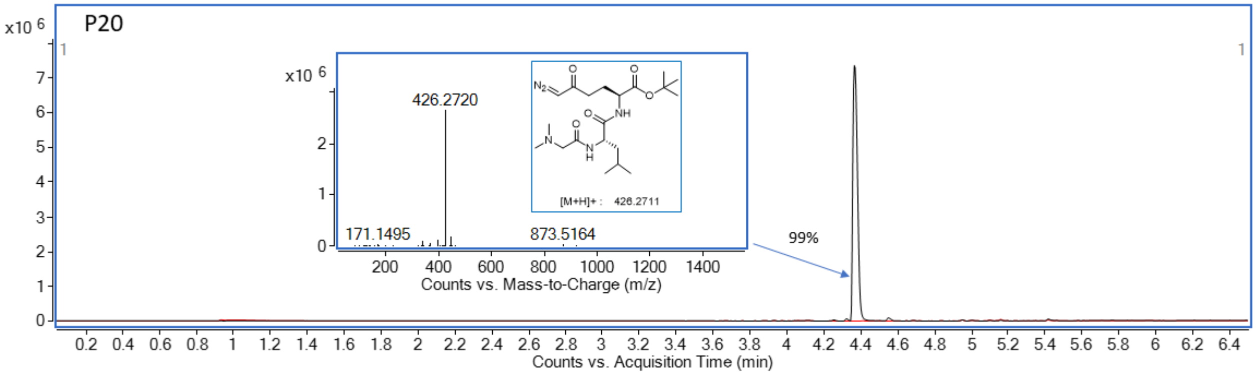

HPLC spectrum of prodrug **P21**

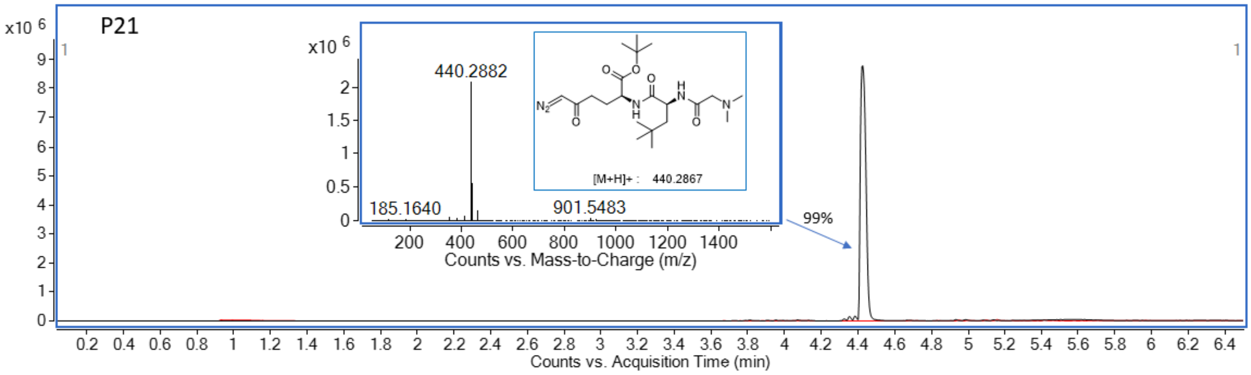

Table S1: clogD$_{7.4}$*values of prodrugs P1-P21 and DRP-104

Cmpd #	clogD
**DRP-104**	-0.07
**P1**	-0.84
**P2**	-0.48
**P3**	-0.37
**P4**	-0.06
**P5**	0.21
**P6**	-1.57
**P7**	-0.11
**P8**	0.41
**P9**	-0.01
**P10**	0.21
**P11**	-0.11
**P12**	-0.11
**P13**	-0.40
**P14**	0.34
**P15**	0.03
**P16**	0.03
**P17**	0.77
**P18**	-2.33
**P19**	-1.77
**P20**	-0.51
**P21**	-0.21

**Calculated using Chemaxon online calculator*

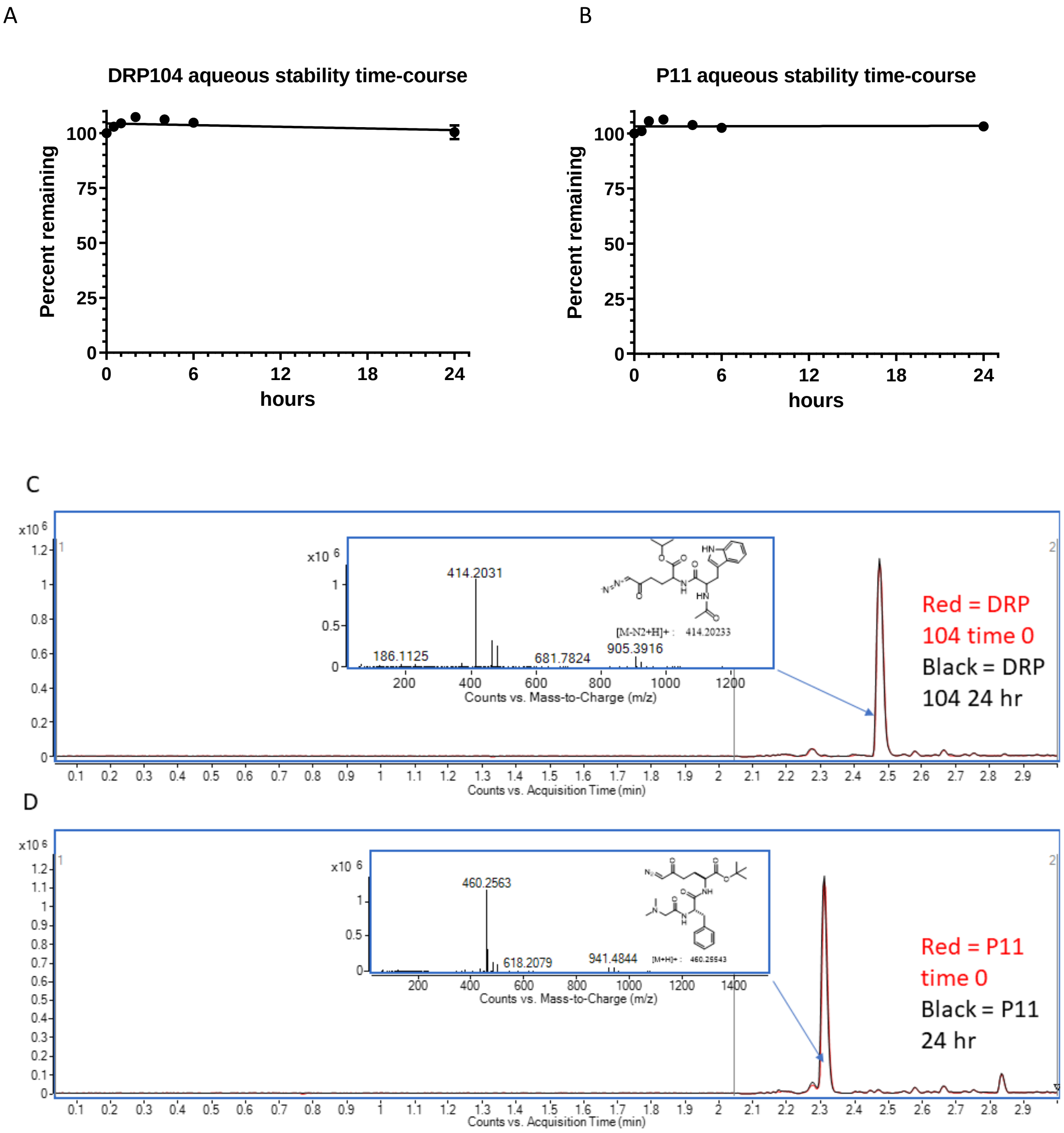

Figure S1. Aqueous stability of DRP 104 and P11. DRP 104 (**A**) and **P11** (**B**) were spiked into PBS (n = 3) and incubated at 37°C. Aliquots were removed at each time-point and percent remaining was measured over 24hr by LCMS; Chromatographic traces of DRP 104 (**C**) and **P11** (**D**) at time 0 vs 24 h showed complete stability with no degradation.
